# Theoretical Investigation of Stacked Two-Dimensional Transition-Metal Dichalcogenide Materials: The Role of Chemical Species and Number of Monolayers

**DOI:** 10.1021/acsomega.4c05423

**Published:** 2025-02-25

**Authors:** Jean M. Bracht, Mateus B. P. Querne, Juarez L. F. Da Silva, Matheus P. Lima

**Affiliations:** † Department of Physics, Federal University of São Carlos, São Carlos, São Paulo 13565-905, Brazil; ‡ São Carlos Institute of Chemistry, University of São Paulo, P.O. Box 780, São Carlos, São Paulo 13560-970, Brazil

## Abstract

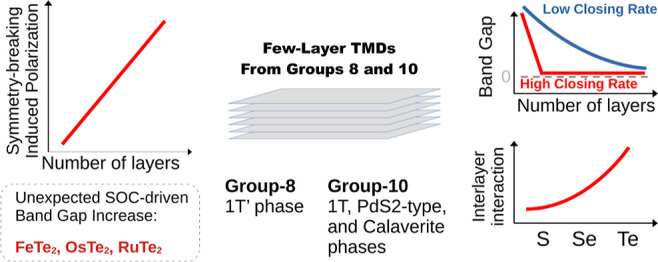

We report a theoretical investigation, based on density functional theory calculations, of the role of chalcogen species and the number of monolayers in the physical–chemical properties of multilayer two-dimensional transition-metal dichalcogenides (TMDs, MQ_2_), where M belongs to groups 8 and 10 of the periodic table, Q = S, Se, or Te, and the multilayer is composed of 1 to 6 layers. From the analysis of structural energetic, and electronic properties, we found significant changes in lattice parameters and exfoliation energies as a function of the number of layers, particularly affected by the chalcogen Q species. The TMDs in group 8 exhibit similar lattice parameters for the same choice of chalcogens, making them suitable for constructing commensurate heterostructures, while the crystal phase and the lattice parameter of the TMDs in group 10 strongly depend on the choice of the transition-metal species. Furthermore, the decreasing trend of electronegativity from S to Te results in stronger exfoliation energies due to lower surface charges, thus governing the structural and electronic characteristics of few-layer TMDs. We find unexpected electronic characteristics, such as band gap increases driven by spin–orbit coupling for certain compositions, the emergence of polarization electric fields due to point inversion symmetry breaking, and semiconductor-to-metal transitions with minimal layer additions to the monolayer. The presence of sulfur improves the sensitivity of the surface properties, enabling precise tuning of band edge positions with the layer number.

## 1 Introduction

The research field of two-dimensional (2D) materials effectively began with the findings of Novoselov et al.,^1^ who not only synthesized graphene but also guided research to demonstrate its unprecedented mechanical,^2^ thermal,^3^ electronic,^4^ and optical^5^ properties. In particular, the atomically thin bipartite honeycomb lattice of graphene results in high sensitivity to stacking patterns, as demonstrated by experiments^6^ and modeled by minimal first-neighbor tight-binding Hamiltonians,^7^ which may have as a unique requirement the capture of the spatial symmetry of those stacks. In fact, few-layer graphene has gained an important subfield in the large area of 2D materials due to its peculiar properties in the scope of fundamental physics and its numerous potential applications, particularly in nanoelectronics.^8^

The unprecedented properties mentioned above found in graphene awaken interest in exploring alternative atomically thin 2D materials, with transition-metal dichalcogenides (TMD)^9^ taking the position of the most investigated materials beyond graphene. These materials also present structures of stacks, such as graphene, in a variety of crystalline structures while keeping the chemical formula MQ_2_, where M is a transition metal and Q = S, Se, or Te. The TMDs also exhibit unprecedented properties, mostly in electronics and optoelectronics,^10^ due to their rich variety of electronic properties, which can be tuned by changing the chemical species or combining several species in the same structure (alloys). Thus, a number of novel applications for TMD-based devices have been proposed, such as sensors,^11^ photovoltaics,^12^ transistors,^13^ and catalysis,^14^ just to cite a few, but not exhausting the possibilities.

The most investigated TMDs are based on W and Mo species;^15−18^ however, recent developments have directed significant attention to group 8 and 10 TMDs, particularly within the realm of experimental investigation. For example, there are reports on those specific TMDs for synthesis/characterization (FeTe_2_,^19^ PtS_2_,^20^ PtSe_2_,^21^ PtTe_2_,^22^ RuS_2_^23^), as well as some applications in photonics (FeS_2_^24^), batteries (FeSe_2_^25^), sensors (NiSe_2_^26^), nanoelectronics (PdS_2_^27^), and hydrogen evolution (PdTe_2_,^28^ RuQ_2_^29,30^).

Along with experimental studies, numerous theoretical studies have been reported so far, either focusing on a specific composition, such as NiS_2_,^31^ PtX_2_ (X = S and Se),^32,33^ OsSe_2_,^34^ OsTe_2_,^35^ and PdSe_2_,^36^ or comparing different compositions between bulk and monolayer forms.^37^ Thus, given the high interest in unveiling the properties of group 8 and 10 TMDs, a deeper understanding of the role of the number of layers for different compositions of these materials is lacking in the literature.

This study investigates 2D structures of TMDs with few layers (from 1 to 6 layers), where M is metals from groups 8 and 10 of the periodic table. In order to obtain the structural and energetic properties, we used density functional theory (DFT) calculations within van der Waals (vdW) corrections, while the electronic properties were obtained considering hybrid functionals and spin–orbit coupling (SOC) effects. Our primary goal is to map the evolution of the structural, energetic, and electronic properties with the number of layers. For example, the exfoliation energy is central to understanding the diverse properties of various compositions, which becomes weaker for heavier chalcogen species.

We found that materials with sulfur in their compositions have a noticeable surface property tuning with the number of layers, making them attractive for catalysis. Moreover, we identify unconventional properties such as (i) electric field polarization for systems without point inversion symmetry, among which FeS_2_, RuS_2_, RuSe_2_, OsS_2_, and OsSe_2_ stand out; (ii) band gap increase driven by spin–orbit coupling, which is larger than 100 meV for FeTe_2_, OsTe_2_, and RuTe_2_; and (iii) band gaps with a high closing rate with the number of layers for PtSe_2_, PdSe_2_, and all Te- and Ni-based materials. Most of them experience a semiconductor-to-metal transition from monolayers to bilayers.

## 2 Theoretical Approach and Computational Details

### 2.1 Total Energy Calculations

Our calculations were based on DFT,^38,39^ as implemented in the Vienna Ab initio Simulation Package (VASP),^40,41^ version 5.4.4, where the frozen-core projector augmented-wave (PAW) method^42,43^ is used to describe the interactions between the core and the valence electrons.^44^ It has been widely known that the generalized gradient approximation (GGA) cannot provide an accurate description of the long-range vdW interactions, which affects the interlayer distances and binding energies between monolayers stacked in van der Waals structures.^45,46^ Furthermore, plain DFT–GGA faces challenges in providing accurate electronic energy band gaps for semiconductor materials.^47^ Therefore, to minimize these problems, the following framework was used:

- The geometric optimizations and energetic properties were calculated using the semilocal formulation proposed by Perdew–Burke–Ernzerhof (PBE),^48^ which is complemented by the addition of the semiempirical vdW D3 correction for the DFT–PBE framework.^49^ It provides a substantial improvement in the description of the geometric and energetic properties of layered systems.^46,50,51^
- DFT–PBE + D3 underestimates the band gap due to the self-interaction errors inherent to the semilocal GGA functionals. Thus, to minimize this problem, the electronic band gaps were calculated using the hybrid Heyd–Scuseria–Ernzerhof (HSE06) functional^52^ using the PBE + D3 equilibrium structures.
- Heavy atoms introduce relativistic effects in the electronic structure of valence electrons, and hence, we also evaluated band gaps with the addition of SOC effects in the second-order approach.^53^

Furthermore, the lack of an inversion symmetry center generates intrinsic polarization dipoles.^54^ Thus, all the results reported in this article include dipole correction.^55^

Our geometry optimizations minimize the stress tensor components in the planar directions and the atomic forces in all directions using the conjugate gradient algorithm.^56^ The equilibrium structures were obtained once the atomic forces on each atom were smaller than 0.010 eV/Å. For stress tensor calculations, we used a plane-wave cutoff energy of 690 eV, which is required to mitigate residual Pulay stress effects, which stem from the incompleteness of the plane wave basis set.^57^ However, a plane wave cutoff energy of 490 eV was used to evaluate the energetic and electronic properties.

Additionally, a vacuum layer of at least 15 Å avoids undesirable interactions between the periodic images of the layered systems. For the integration of the Brillouin zone, we used a sampling of the reciprocal space defined by the following **k**-meshes: 14 × 14 × 1 for NiS_2_; 13 × 13 × 1 for NiSe_2_; 12 × 12 × 1 for PdSe_2_; 11 × 11 × 1 for PtTe_2_; 7 × 7 × 1 for PdS_2_; 8 × 12 × 1 for FeS_2_; 7 × 12 × 1 for FeSe_2_ and RuS_2_; 7 × 11 × 1 for FeTe_2_, RuSe_2_, OsS_2_, and OsSe_2_; 7 × 10 × 1 for RuTe_2_ and OsTe_2_; 6 × 11 × 1 for the PtS_2_ and PtSe_2_; and 6 × 10 × 1 for PdTe_2_. For all calculations, the self-consistency electron density was achieved using an energy criterion of 1 × 10^–6^ eV.

### 2.2 Design of Stacked 2D Structure Models

The few-layer materials investigated here were previously studied by our research groups in their bulk and monolayer forms,^37^ where promising properties were demonstrated for applications in electronics and energy conversion devices. We consider 17 compositions represented by the chemical formula MQ_2_, where M = Fe, Ni, Ru, Pd, Os, and Pt; and Q = S, Se, and Te. Some investigated materials have already been synthesized, which corroborates the theoretical predictions of their stabilities. These include FeS_2_,^24^ FeSe_2_,^25^ FeTe_2_,^19^ NiSe_2_,^26^ PdS_2_,^27^ PdTe_2_,^28^ PtS_2_,^20^ PtSe_2_,^21^ PtTe_2_,^22^ RuS_2_,^23^ RuSe_2_,^29^ and RuTe_2_.^30^

**Figure 1:**
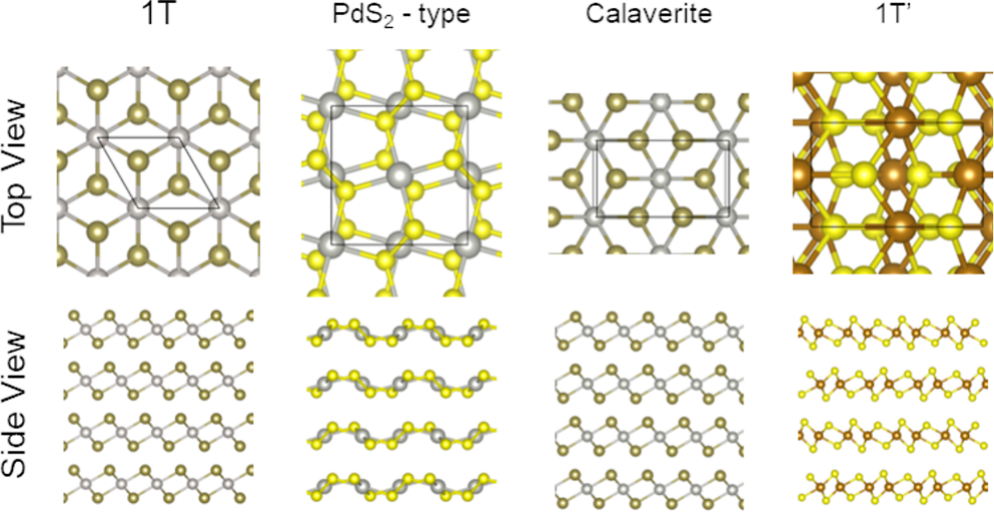
Ball-and-stick representation of crystalline structures: 1*T*′, PdS_2_-type, Calaverite, and 1*T* phases exemplified for FeS_2_, PdS_2_, PdTe_2_, and PtTe_2_, respectively. Top panels depict top views with unit cells outlined as solid lines, while the bottom panels show side views of structures with four layers (*n* = 4). Our investigation encompasses materials of the form MQ_2_, where M (e.g., Fe, Ni, Os, Pd, Pt, Ru) and Q (e.g., S, Se, Te) vary, covering layers 1 < *n* < 6.

Each material is simulated in its lowest energy layered crystalline structure, and for these compositions, there are four possibilities:^37,58^ 1*T*, 1*T*′, Calaverite, and PdS_2_-type, as shown in Figure 1, and described below:

- The 1*T* structure occurs for NiS_2_, NiSe_2_, PdSe_2_, and PtTe_2_.^59^ It has a hexagonal unit cell belonging to the *P*3*m*_1_ space group and is formed by octahedral coordinated transition-metal atoms to six chalcogens.
- The 1*T*′ crystalline structure is observed for FeS_2_, FeSe_2_, FeTe_2_, RuS_2_, RuSe_2_, RuTe_2_, OsS_2_, OsSe_2_, and OsTe_2_ compounds^60,61^ and has an orthorhombic unit cell belonging to the *Pnm*2_1_ space group. This structure can be built through a 2 × 1 1*T* monolayer supercell with the addition of dimerizations of rows of metal atoms.
- The Calaverite structure occurs for compounds PdTe_2_, PtS_2_, and PtSe_2_^62^ and has a monoclinic unit cell centered on the base that belongs to the *C*12/*m*_1_ space group. It is a distorted 1*T* structure.
- The PdS_2_-type structure is adopted solely for PdS_2_^61^ and has a tetragonal unit cell in the *Pbca* space group. This structure has one Q–Q bond and four M–Q bonds forming pentagonal rings that constitute puckered layers.

To construct the initial geometries for the few-layer systems, we first performed geometry optimizations on bulk vdW crystals. Using these optimized bulk structures, we then built few-layer systems with *n* layers, where 1 ≤ *n* ≤ 6, by repeating the unit cell in the *z* direction and introducing a vacuum layer by increasing the lattice vector perpendicular to the monolayers.

Despite investigating a few layers comprising stacked sheets with equivalent chemical compositions and crystalline phases, some of them lack point inversion symmetry because of a combination of the stacking pattern and the internal space group, which generate asymmetric charge transfers between the layers induced by peculiar interlayer interactions. Consequently, charge polarization arises, resulting in different physical properties for the surfaces on each side of the slab such as work function. It is important to stress that unlike 2D Janus materials,^63^ which have polarization electric fields induced by surface compositions, the internal electric fields of few-layer TMDs arise solely from symmetry features.

Moreover, the long-range feature of the polarization electric field adds spurious interactions between the periodic images, generating errors in obtaining structural and electronic properties, such as lattice parameters and band gaps.^54^ Thus, the addition of dipole corrections, consisting of the inclusion of an external electric field in all calculations, solved this issue.^49^ Among the investigated systems, FeS_2_, FeSe_2_, FeTe_2_, OsS_2_, OsSe_2_, OsTe_2_, PdS_2_ (2, 4, and 6 layers), RuS_2_, RuSe_2_, and RuTe_2_ lack point inversion symmetry.

## 3 Results and Discussion

The results and discussion are organized as follows: We first analyze the bulk of the vdW crystals to validate our simulations against comparison with the published work and the reference properties of the state in the bulk limit (Section 3.1). Thus, we investigate the magnetism that hinders monolayers driven by lattice distortions (Section 3.2) as all investigated materials are nonmagnetic. Subsequently, we discuss the role of the number of layers on the structural (Section 3.3), energetic (Section 3.4), and electronic properties (Section 3.5). Then we contrast the band edge alignments among all materials (Section 3.6) and finalize the presentation of our results by evaluating the polarization induced by symmetry breaking with the number of layers (Section 3.7).

### 3.1 Crystalline Phase Analysis

Table 1 identifies the crystalline phase of each structure and presents the planar lattice parameters, namely, *a*_0_ and *b*_0_. These values are in good agreement with previously reported data from available databases^64,65^ and with our previous study.^37^ For group 8 TMDs, an analysis of these lattice parameters shows a great influence of chalcogens rather than metal species. For example, 1*T*′-RuS_2_ has *a*_0_ = 5.54 Å, and this value increases to 6.09 Å when replacing S with Te (forming 1*T*′-RuTe_2_), representing a difference of 9.03%. On the other hand, the metal species have less impact on the lattice parameter, as evident from the particular analysis of OsSe_2_ and RuSe_2_, whose *a*_0_ values are 5.74 and 5.76 Å, respectively (representing a mismatch of only 0.35%), despite these metals belonging to different lines in the periodic table.

**Table 1:** Bulk Properties of the MQ_2_ Systems^a^

MQ_2_	phase	*a*_0_ (Å)	*b*_0_ (Å)	*E*_g_^PBE^ (eV)	*E*_g_ (eV)
FeS_2_	1*T*′	5.296	3.229	–0.004	0.238
FeSe_2_	1*T*′	5.552	3.401	*M*	*M*
FeTe_2_	1*T*′	5.918	3.685	*M*	*M*
RuS_2_	1*T*′	5.541	3.465	0.282	0.723
RuSe_2_	1*T*′	5.762	3.611	0.032	0.367
RuTe_2_	1*T*′	6.093	3.863	*M*	*M*
OsS_2_	1*T*′	5.518	3.527	0.668	1.176
OsSe_2_	1*T*′	5.738	3.679	0.455	0.753
OsTe_2_	1*T*′	6.081	3.918	*M*	*M*
NiS_2_	1*T*	3.389	3.389	*M*	*M*
NiSe_2_	1*T*	3.592	3.592	*M*	*M*
PdS_2_	PdS_2_-type	5.520	5.588	–0.214	0.665
PdSe_2_	1*T*	3.782	3.782	*M*	*M*
PdTe_2_	calaverite	7.041	4.073	*M*	*M*
PtS_2_	calaverite	6.244	3.606	0.049	0.513
PtSe_2_	calaverite	6.567	3.791	*M*	*M*
PtTe_2_	1*T*	4.068	4.068	*M*	*M*

a The column “phase” specifies the structure, whereas *a*_0_ and *b*_0_ are the lattice parameters, *E*_g_^PBE^ represents the band gap at the PBE calculation level, and *E*_g_ is the corrected band gap (our best estimate), both calculated from the difference in energy between the valence band maximum and conduction band minimum. Negative values indicate semi-metal behavior, whereas *M* indicates metallic behavior.

On the other hand, the group 10 TMDs change the crystalline phase for different metals, and a direct comparison between their lattice parameters is suitable only between NiSe_2_ and PdSe_2_ as both assume the 1*T* phase. In this case, we found an increase in the lattice parameter of 5.3%. Thus, group 10 has a stronger influence of the metal species on their structural properties than group 8 TMDs.

Table 1 also presents band gaps calculated within the PBE level (*E*_g_^PBE^) in addition to corrected band gaps (*E*_g_), which include effects from hybrid exchange-correlation energy functional (*E*_xc_) and SOC. First, we obtained metallic behavior for numerous materials confirmed by both the PBE and corrected approaches. However, semimetallic behaviors are predicted for FeS_2_ and PdS_2_ within the PBE approach; but, the band gap corrections revert the semimetallic behavior to semiconductor ones (by opening band gaps in these materials). For semiconductor materials, all band gap values are lower than 1.2 eV, and band gap corrections are shown to be of great importance.

### 3.2 Magnetism Hindering from 1*T* → 1*T*′ Phase Transition

Magnetic 2D materials are a topic of great interest due to the prediction of intriguing physical properties, such as the quantum anomalous Hall effect^66^ and potential spintronics applications.^67^ In the context of TMDs, the magnetic moment should arise from half-filled 3d, 4d, or 5d shells, as in Fe. However, an exploration of the spin-resolved electronic density of our investigated few-layer systems demonstrates nonmagnetic behavior. For instance, this absence of magnetism does not agree with other recent works, such as the investigations of Wang et al.,^68^ which explore complex magnetism in 2D FeS_2_. The origin of this discrepancy is the consideration of distinct structural phases: (i) we adopt the 1*T*′ phase, which can be understood as a 2 × 1 1*T* cell with additional transition metal dimerization;^60^ (ii) Wang et al. adopt the simpler hexagonal 1*T* structure. Thus, the transition from the 1*T* phase to the 1*T*′ phase hinders the magnetism in FeS_2_. We also show here that these results can be generalized for group 8 TMDs.

Table 2 shows some properties for all group 8 TMDs in the metastable 1*T* phase. We first note that our calculated lattice parameter and magnetic moment are close to those predicted by Wang et al., i.e., 3.23–3.28 Å and 1.8–2.0 μ_B_, respectively. Table 2 also shows the relative energy Δ*E*_tot_ (that is, the total energy difference between the 1*T* and 1*T*′ phases), demonstrating that 1*T*′ is more stable than the 1*T* phase. Moreover, the remaining data in Table 2 demonstrate that this magnetism hindering induced by the 1*T* to 1*T*′ phase transition is present in all structures from group 8. Moreover, Δ*E*_tot_ increases with the transition-metal weight and decreases with the chalcogen weight.

**Table 2:** Monolayer MQ_2_ Properties in the Metastable 1*T* (Hexagonal) Phase^a^

MQ_2_	phase	*a*_0_ (Å)	*m* (μ_B_)	Δ*E*_tot_ (meV)
FeS_2_	1*T*	3.18	1.77	202
FeSe_2_	1*T*	3.32	1.68	170
FeTe_2_	1*T*	3.58	1.44	186
RuS_2_	1*T*	3.32	1.96	733
RuSe_2_	1*T*	3.44	1.60	701
RuTe_2_	1*T*	3.69	0.96	641
OsS_2_	1*T*	3.39	1.87	882
OsSe_2_	1*T*	3.53	1.98	891
OsTe_2_	1*T*	3.84	0.37	769

a *a*_0_ represents the lattice parameters, *m* denotes the magnetic moment, and Δ*E*_tot_ indicates the relative energy calculated as half of the total energy difference between the 2 × 1 1*T* and the 1 × 1 1*T*′ monolayers. Here, we consider only systems with non-magnetic ground states in the 1*T*′ phase. These results are obtained using the PBE framework.

**Figure 2:**
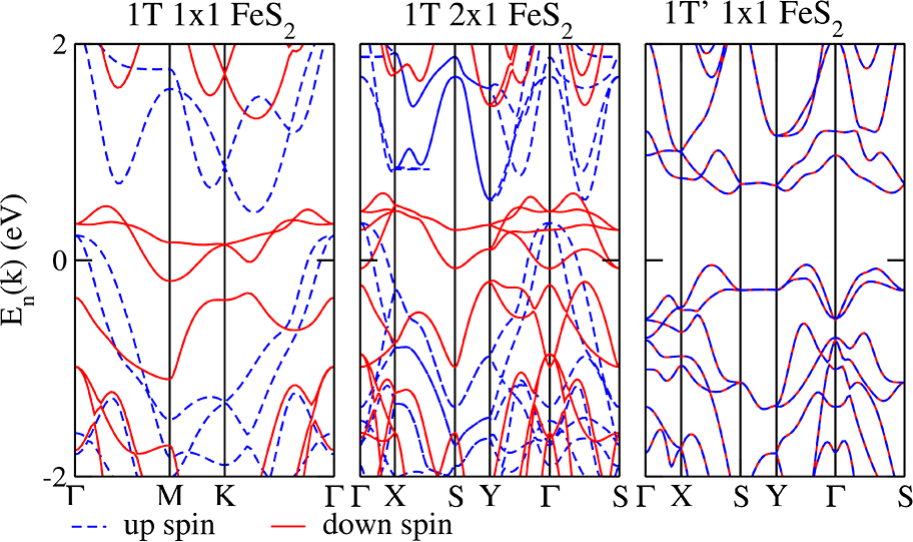
Electronic band structures for FeS_2_ monolayers evaluated within the DFT–PBE + D3 framework. Left panel: hexagonal 1*T* with a 1 × 1 cell; middle panel: 1*T* phase with a 2 × 1 cell; right panel: 1*T*′ unit cell, i.e., equivalent to a distorted 1*T* 2 × 1 cell.

We also analyze the electronic features of the band structures, as shown in Figure 2. The left panel shows the band structure for 1 × 1 1*T* FeS_2_, which clearly indicates its spin polarization. In the middle panel, we generate a 2 × 1 band structure for 1*T* FeS_2_, where the band folding also results in a magnetic configuration. However, transition metal dimerization takes place in the 1*T*′ phase (Figure 2, right panel), and the band for the spin-up is equal to the band for the spin-down, undoubtedly demonstrating magnetism hindering induced by the 1*T* to 1*T*′ phase transition.

### 3.3 Layers’ Impact on the Equilibrium Lattice Parameters

**Figure 3:**
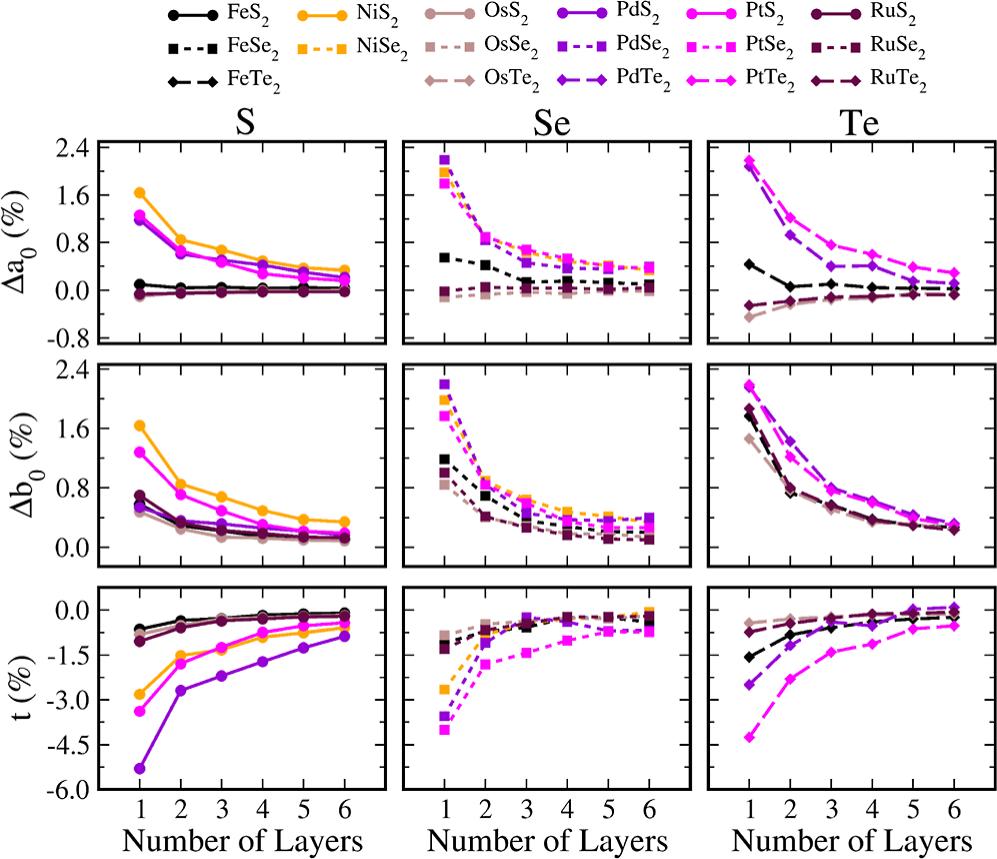
Percentual variation in planar lattice parameters *a*_0_ (top panels), *b*_0_ (middle panels), and layer thickness *t* (bottom panels) from their bulk values plotted as a function of the number of layers. We define the percentual variation as Δ*l* = (*l*^bulk^ – *l*^*n*^)/*l*^bulk^ × 100, where, *l* = *a*_0_, *b*_0_, or *t*.

Figure 3 presents the percentage variation of the geometrical parameters with the number of layers of their bulk values. First, we analyze the role of *n* in *a*_0_ and *b*_0_, and we notice that all parameters monotonically tend to their bulk values as *n* increases, as expected because the surface effects become less relevant against bulk effects for systems with more layers. Moreover, we find significant percentage variations, up to 2.3% for PtTe_2_ with *n* = 1. For reference, it is worth noting that variations in the lattice parameters up to 3% can be considered large in experiments.^69^ It is also noted that the variations of the lattice parameters are all positive with *n*, except for *b*_0_ of the RuTe_2_ and the OsTe_2_ monolayers.

From Figure 3, it is also evident that the group of the periodic table to which the metal belongs (8 or 10) influences the variation of the lattice parameter as there is a tendency of the curves with metals in the same periodic table metal group to aggregate. For example, the curves for *a*_0_ with *n* split the values for FeQ_2_, RuQ_2_, and OsQ_2_ (lower group) from those of NiQ_2_, PdQ_2_, and PtQ_2_ (higher group). Moreover, there is a slight separation of FeQ_2_ from its group for FeSe_2_ and FeTe_2_. A similar pattern but less evident occurs for *b*_0_. As an exception, *b*_0_ for PdS_2_ approaches the other group, a fact attributed to the peculiar relation, *a*_0_ = *b*_0_ for this system.

There are also effects of the chalcogen species on the dependence of the lattice parameter on *n*. Despite the effects of the metal group on the periodic table and the general shape of the curves similar to those for all systems, the percentage variations become more pronounced following the sequence S → Se → Te. In anticipation of one of our main findings, these effects relate to the strengthening of the interlayer interaction with the increase of the chalcogen height.

Finally, Figure 3 also presents the average percentage variation for the thickness of the layer, *t*, as a function of *n*. Despite the asymptotic behavior that tends to the bulk values also occurring, the variation has the opposite direction to *a*_0_ and *b*_0_, that is, the percentage variation is negative for single layers and gradually increases for the bulk values, indicating its decrease with *n*. Moreover, it is also notable in the effects of metal groups (8 or 10) and chalcogen species but less evident compared to *a*_0_. However, the range of values for *t*, reaching −5% for PdS_2_, as percentage variations in the layer thickness induced by long-range interactions, are rare in other systems. As a final remark, we point out that the geometrical parameters present similar variations for a set of systems with distinct crystalline structures. Then, these percentage variations in the geometrical properties with the number of layers fundamentally depend on the composition.

These lattice parameter variations with the number of layers are not expected for most layered systems because of the weak interlayer van der Waals interaction. For example, few-layer graphene,^70^ SnS, SnSe,^71^ and WS_2_^72^ maintain the same lattice parameters as the number of layers varies, despite the fact that there is a strong tuning of their electronic properties. To understand this feature, it is worth considering that the interactions between layers in these systems are mainly due to the interaction between π orbitals, while the lattice parameter is mostly determined by σ interactions compounding the intralayer covalent chemical bonds. However, in the literature, there are materials with large variations in the lattice parameters with *n*, such as in the examples of ZnO^73^ and PtSe_2_,^74^ which better align with our results.

### 3.4 Exfoliation Energy Variation Patterns

The exfoliation energy (*E*_exf_) quantifies the energy necessary to split the systems into isolated monolayers, which can be used to estimate their energetic stability.^75^ Thus, *E*_exf_ is given by the following equation
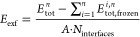
1where *E*_tot_^*n*^ is the total energy for the target system containing *n* layers, *E*_tot,frozen_^*i*,*n*^ is the total energy for the *i*-th layer isolated from the others and kept frozen as in the target system, *A* is the area of the unit cell, and *N*_interfaces_ is the number of interfaces between monolayers in the target system, being *n* – 1 for few-layer and *n* for van der Waals crystals. *E*_exf_ calculated with frozen monolayers is well approaching the adsorption energy *E*_ads_, which is calculated with optimized monolayer geometries. Thus, we choose to present in the main article only the values for *E*_exf_ due to the lower computational cost and the possibility of comparing them with other reported results.^37,76^

**Figure 4:**
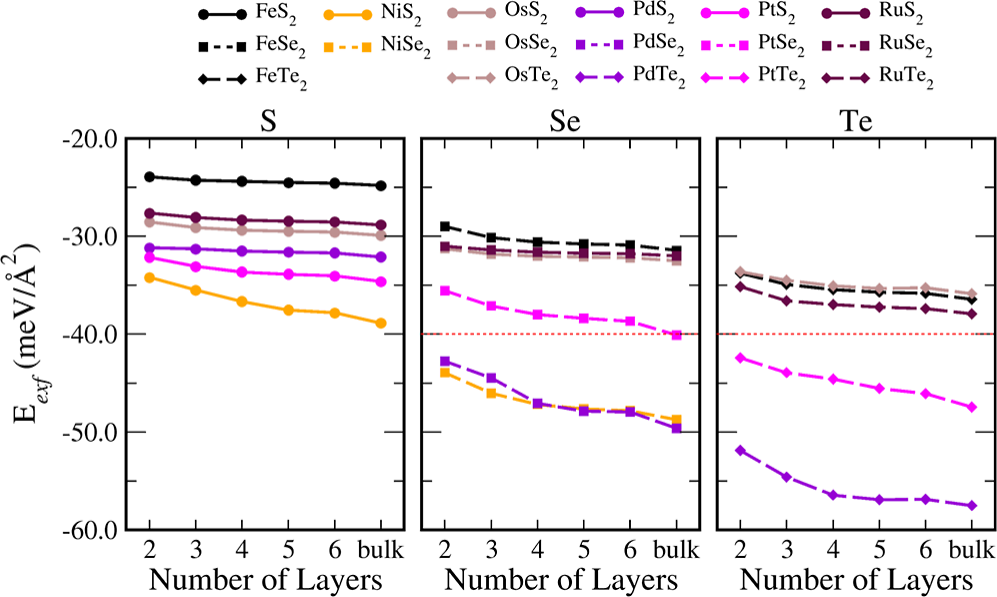
Exfoliation energy (*E*_exf_) versus the number of layers evaluated with the PBE + D3 framework. The red dotted lines represent the weak interaction threshold of 40 meV/Å.

Recent theoretical investigations on layered TMDs roughly suggested the threshold of 40 meV for *E*_exf_ above which van der Waals forces dominate the interlayer interactions.^37,77,78^Figure 4 shows *E*_exf_ as a function of *n*, demonstrating enhanced interlayer interactions with the number of layers for all materials (i.e., *E*_exf_ decreases with *n*). However, large variations with *n* occur for PdSe_2_ (1*T*) and NiSe_2_ (1*T*), whereas NiS_2_ (1*T*) presents an almost constant *E*_exf_. These larger variations do not exceed 15% of their absolute values; therefore, the number of layers does not alter the mechanism of interaction between layers, serving only as a refinement for *E*_exf_. The crystalline phase does not play a relevant role, suggesting that the composition is the dominant feature of these interlayer interactions.

An analysis of the role of the metal species in *E*_exf_ also indicates different behaviors between metals belonging to groups 8 and 10. The prior set of compounds (FeQ_2_, RuQ_2_, and OsQ_2_) present weakened *E*_exf_, while the converse is true for group 10 materials. This clustering of behaviors for the exfoliation energy perfectly connects to variations of the lattice parameters since the shorter variations (for example, for Δ*a*_0_) occur for systems with weakened *E*_exf_.

Changes in chalcogen species induce the most outstanding effects in *E*_exf_ as interlayer interactions become stronger for heavier chalcogens (i.e., S → Se → Te). We ascribe to this behavior the electronegative (Ξ) decrease with the chalcogen mass (Ξ_S_ > Ξ_Se_ > Ξ_Te_)^79^ because all investigated crystal phases have chalcogen positioned at their surfaces, and the lower electronegativity results in a lower charge at the surfaces’ atoms, which reduces the Coulomb repulsion that opposes long-range attraction forces, such as vdW and quadrupole interactions. Thus, lower chalcogen electronegativities result in stronger interlayer interactions, reflecting lower values for *E*_exf_.

### 3.5 Layer-Dependent Band Structures

All investigated materials present significant changes in the band structure with the number of layers. In addition, we found distinct characteristics among the investigated materials. First, we note some materials narrowing their band gaps with *n* on a low closing rate and others with a high closing rate. Although all materials present a band gap reduction as the number of layers increases due to quantum confinement effects (as expected), some of them undergo a semiconductor–metal transition by adding only one or two more layers, despite starting from significant band gap values for *n* = 1, while other materials narrow their band gaps moderately as *n* increases.

**Figure 5:**
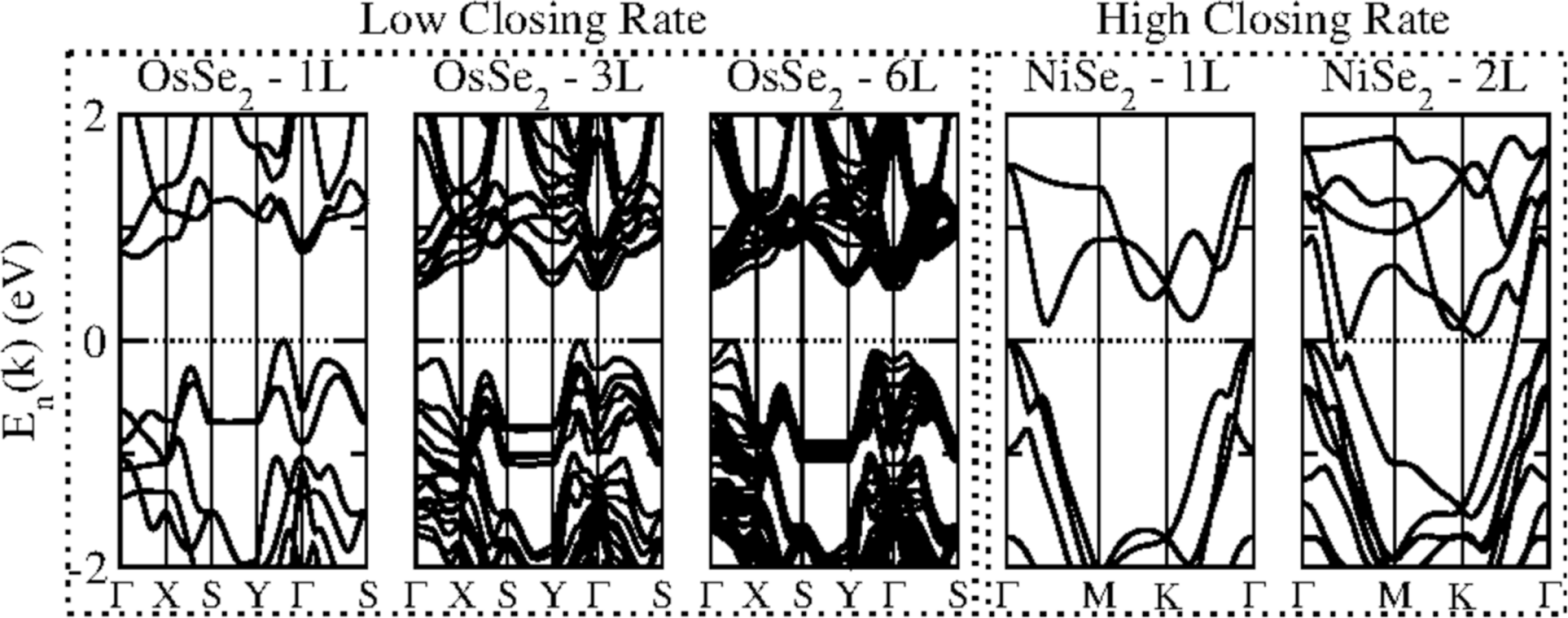
Examples of band gaps with high and low closing rates with *n*. The three left panels labeled as “Low Closing Rate” depict OsSe_2_ with *n* = 1, 3, and 6, while the two righter panels labeled as “High Closing Rate” show band structures for NiSe_2_ with *n* = 1 and 2, all calculated within the PBE + D3 level.

Figure 5 shows the band structure for OsSe_2_ as an example of a material with a low closing rate and NiSe_2_ as an example of a material with a high closing rate. We ascribe these changes to the strength of the interlayer binding interactions. The high-closing-rate materials are associated with strong interlayer interactions, whereas the converse is true for low-closing-rate materials. That is, high-closing-rate materials are PtSe_2_ and PdSe_2_, in addition to all TMDs based on Te and Ni. All of the remaining materials present low-closing-rate behaviors.

**Figure 6:**
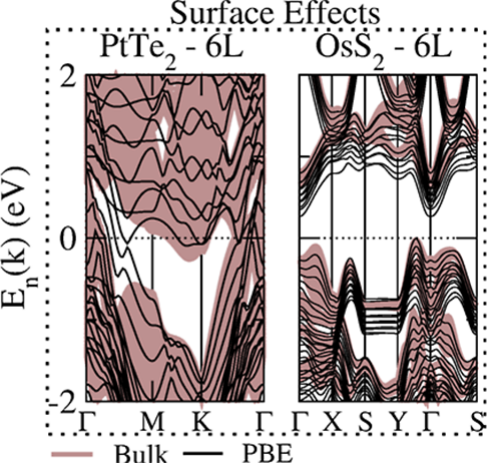
Surface effects at the electronic band structures showcase for PtTe_2_ and OsS_2_ with *n* = 6. The solid areas represent the bulk band structure projected onto the **k**-path with *k*_z_ = 0 calculated with the PBE + D3 framework.

Another peculiarity consists of two particular classes of surface states, exemplified in Figure 6 for PtTe_2_ and OsS_2_. We identified surface states by comparing the electronic band structure for few-layer TMDs with the bulk band structures projected onto the **k**-path of the few-layer band structure in the Brillouin zone. PtTe_2_ with *n* = 6 presents a metallic band structure; however, it has additional states in the Γ – *M* path around the Fermi level that are not present in the projected bulk band structure. Other few-layer TMDs present similar features such as NiS_2_, NiSe_2_, PdSe_2_, PtS_2_, and PtSe_2_. On the other hand, other materials, such as OsS_2_ with *n* = 6, shown in Figure 6, exhibit flat states of spread energy, which in this case are located in the *S* – *Y***k**-path (similar to OsSe_2_, RuS_2_, and RuSe_2_). These surface states arise from a combination of structural deformations (that make each monolayer of the few-layer TMD nonequivalent with respect to the others) and interlayer interactions (which split these flat energy bands).

#### 3.5.1 Band Gap Trends with Layer Number

We corrected the band gap values within the scissors operator approach for hybrid *E*_xc_ (χ^HSE06^) and spin–orbit coupling (χ^SOC^) by (*i*) χ^HSE06^ = *E*_g_^HSE06^ – *E*_g_^PBE + D3^ and (*ii*) χ^SOC^ = *E*_g_^PBE + D3^ – *E*_g_^PBE + D3 + SOC^, while *E*_g_^HSE06^, *E*_g_^PBE + D3^, and *E*_g_^PBE + D3 + SOC^ represent band gaps evaluated within the HSE06, PBE + D3, and PBE + D3 + SOC approaches, respectively. That is, χ^HSE06^ is the band gap increasing due to self-interaction corrections, whereas χ^SOC^ is the band gap reduction due to spin–orbit-coupling effects. We further optimized the use of computational resources for these calculations by considering only the **k**-points of the valence band maximum (VBM) and conduction band minimum (CBM) obtained from PBE + D3 calculations (not the entire electronic band structure) to evaluate the band gap corrections χ^HSE06^ and χ^SOC^ and by parsing the **k**-mesh for sampling the reciprocal space with the use of *R*_**k**_ = 20 Å and to evaluate those band gaps.

**Figure 7:**
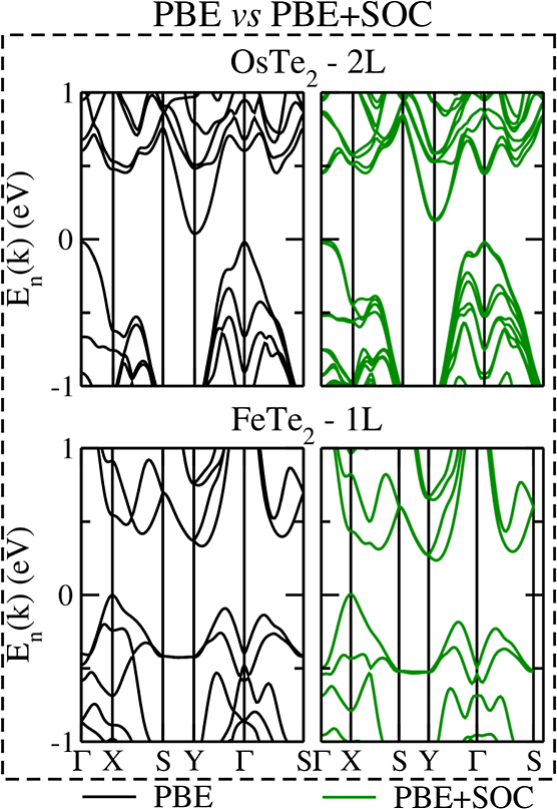
Representation of spin–orbit coupling effects in the electronic band structure. The left and right panels contrast the PBE and PBE + SOC approaches, respectively, for OsTe_2_ with *n* = 2 (upper panels) and FeS_2_ with *n* = 1 (bottom panels).

Now, we focus our analysis on SOC effects, which are expected to generate substantial changes in the electronic band structure in systems that have heavy atoms such as Te, Os, and Pt in their compositions. Generally, a band gap reduction is often observed with the inclusion of the SOC. However, notably, some investigated few-layer TMDs exhibit an unconventional band gap increase with the inclusion of spin–orbit coupling. Figure 7 showcases a spin–orbit-coupling-driven band gap increase for the particular case of OsTe_2_ with *n* = 2 and the regular SOC-driven band gap decreasing for FeTe_2_ with *n* = 2.

We identified SOC-driven band gap increases by more than 0.1 eV for FeTe_2_, OsTe_2_, and RuTe_2_ and less prominently for PtS_2_, PtSe_2_, RuSe_2_, and PdS_2_. Note that the remaining materials exhibit the expected band gap reduction with the inclusion of spin–orbit coupling. Since our band structures with spin–orbit coupling were obtained from frozen geometries resulting from PBE + D3 calculations, this peculiar band gap increase driven by spin–orbit coupling is purely electronic, and we attribute it to a peculiar splitting of states mainly around the Γ-point propagating to the entire band structure.

For the sake of completeness, we also analyze the effect of the HSE06 *E*_xc_ band structure on the PBE + D3 approach. All materials exhibit expected band gap widening with the use of the hybrid *E*_xc_, differing only in absolute values within the range of 0.2 to 1.0 eV, considering only semiconductor materials (we do not use this approach for metallic systems). However, the hybrid functional alters the shapes of both the valence and conduction bands. Localized (flat) regions experience larger corrections, whereas dispersive regions shift by smaller energies, as expected. Thus, the hybrid *E*_xc_ could be used to predict accurate band gap values but may have some influence on the shape of band structures at higher energies, potentially affecting optical properties such as absorption or refractive index.

**Figure 8:**
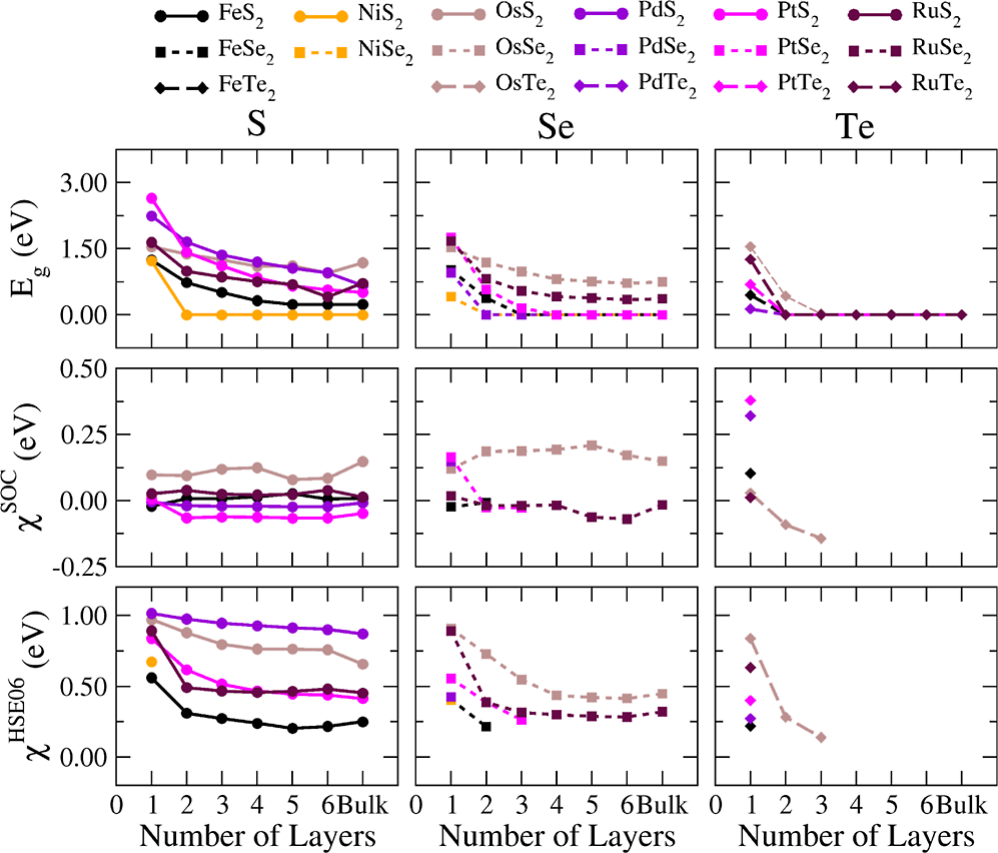
Fundamental band gaps (*E*_g_) (upper panels) as a function of the number of layers calculated as the difference between the CBM and VBM including corrections only for semiconductor systems (not for metallic ones) due to spin–orbit coupling χ^SOC^ (middle panels) and HSE06 χ^HSE06^ (bottom panels). Here, *E*_g_ = *E*_g_^PBE^ – χ^SOC^ + χ^HSE06^, with χ^SOC^ = *E*_g_^PBE^ – *E*_g_^PBE + SOC^ and χ^HSE06^ = *E*_g_^HSE06^ – *E*_g_^PBE^. *E*_g_^PBE^, *E*_g_^PBE + SOC^, and *E*_g_^HSE06^ represent fundamental band gaps calculated within plain PBE + D3, PBE + D3 with SOC, and HSE06 (no SOC) frameworks, respectively.

The upper panels of Figure 8 present our improved band gap estimate for all materials as a function of *n*, allowing us to identify trends in composition and eventually in crystalline structure. Primarily, it is worth noting the high- and low-closed-rate materials, as mentioned earlier. Numerous semiconductor-to-metal transitions, ruled by the number of layers, occur, and it is interesting to observe a substantial band gap narrowing with the addition of one more layer, as seen in the case of NiS_2_, where the band gap narrows by over 1.3 eV from *n* = 1 to *n* = 2.

From an analysis specifically focused on the metal species, we do not identify behavior aggregations for group 8 and 10 metals; that is, the band gap variations and ranges are of the same order for metals in different columns of the periodic table. Additionally, the crystalline phase does not dictate the band gap behaviors. However, there is a clear tendency to narrow band gaps for heavier chalcogens, especially for *n* ≥ 2. Here, since electronic structure tuning closely relates to interlayer interaction strength, the Te-based few-layer TMDs enhance the sensitivity of the band gap with the number of layers due to their stronger interlayer interactions.

The middle and bottom panels of Figure 8 show the band gap corrections due to SOC and the hybrid *E*_xc_. We evaluated these corrections only for semiconductor materials as they are extracted from shifts of the band edges using PBE + D3 as the reference. A prior analysis of the general behavior as a function of *n* reveals that χ^SOC^ assumes a plateau (i.e., an almost constant value independent of the number of layers), while χ^HSE06^ decreases with *n*. This trend of the SOC correction is not surprising as the SOC strength depends mainly on the composition, whereas species with numerous core electrons enhance the effects of the  operator.

On the other hand, the χ^HSE06^ decrease with *n* can be understood considering that states with mostly localized Bloch functions result in higher self-interaction errors.^80^ Thus, the addition of more layers delocalizes the orbitals over all containing monolayers in the few-layer TMDs, hence requiring lower energy band gap corrections to overcome the band gap underestimation inherent to the PBE + D3 approach. Specifically, it is worth noting the peculiar features of some monolayer materials compared to others with *n* > 1. Namely, PtSe_2_ changes the sign and strength of χ^SOC^ (from 164 to −27 meV), whereas RuSe_2_ and OsTe_2_ decrease χ^HSE06^ by less than half their values, all from *n* = 1 to *n* = 2.

Indeed, our results show that the GGA + D3 approach fails to classify even metallic/semiconductor behavior for some materials with a specific number of layers. Here, we note that FeTe_2_, NiS_2_, PdSe_2_, PtTe_2_, and RuTe_2_ with *n* = 2; OsTe_2_ and PtSe_2_ with *n* = 3; FeSe_2_ and PtSe_2_ with *n* = 4; RuSe_2_ with *n* = 6 present a semimetal^81^ band structure with nonoverlapping valence and conduction bands at the PBE + D3 level, opening the possibility of band gap openings with the inclusion of band gap corrections. Thus, we apply band gap corrections to these materials at both the SOC and HSE06 levels. Notably, band gap openings occur only for OsTe_2_ and PtSe_2_ with *n* = 3 beyond RuSe_2_ with *n* = 6, whereas the latter band gap reaches 0.35 eV, indicating not only the metal-to-semiconductor transition but also a substantial band gap value considering the room temperature reference. Therefore, band gap corrections deserve attention for electronic structure classifications of few-layer TMDs.

### 3.6 Role of the Number of Layers in the Band Edge Alignments

The work function is the minimum energy required to remove an electron from a surface and is determined as the energy difference between the Hartree potential plateau in the vacuum region adjacent to a given surface and the VBM energy, which equals the VBM value at 0 K.^76^ At the same level of approach, the electronic affinity equals the CBM value.

However, it is worth noting that the polarization electric fields arising from the lack of point inversion symmetry result in different plateaus for the Hartree potential in the vacuum region for each surface side and thus in two work function values termed here as Φ^+^ and Φ^–^, one for each slab surface side. The electron affinity also has two values, EA^+^ and EA^–^ determined by Φ^+^ and Φ^–^ plus the band gap value, respectively. The symbols + and – refer to higher and lower values, respectively. Regarding the use of *E*_xc_ for these simulations, we determined the Hartree potential, the band gap value, and the VBM within the HSE06 *E*_xc_; however, we further corrected the last two quantities by adding to them a shift due to SOC determined from a comparison between the band edges’ energy positions for PBE + D3 and PBE + D3 + SOC simulations.

**Figure 9:**
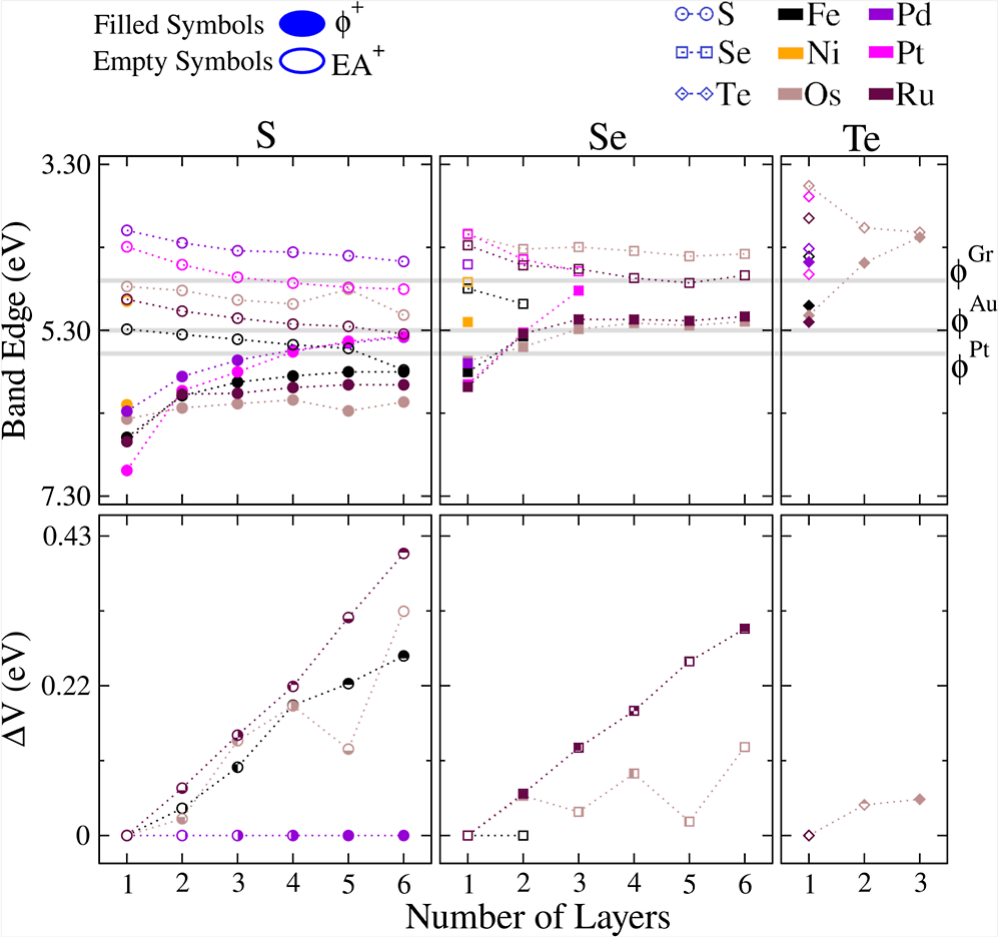
Work function and ionization potentials as a function of the number of layers. The top panels present the higher work functions (ϕ^+^) and electron affinities (EA^+^). The bottom panels show the difference between higher and lower work functions (Δ*V* = ϕ^+^ – ϕ^–^) for materials without point inversion symmetry (Δ*V* = 0 for the others). In these calculations, the valence band maximum (VBM) and conduction band minimum (CBM) were obtained within the HSE06 *E*_xc_ framework and corrected due to spin–orbit coupling (SOC). The gray horizontal lines indicate experimental work functions for graphene (ϕ^*Gr*^),^82^ gold (ϕ^*Au*^),^83^ and platinum (ϕ^*Pt*^).^84^

The position of the edge of the band (VBM and CBM) provides substantial information on 2D materials in the scope of heterostructures^85^ and water splitting,^86^ just to cite a few. The upside of Figure 9 shows the distance in energy from the VBM and CBM to the vacuum energy as a function of the number of layers. Here, changes in the number of layers induce variations of only tenths of electron volts, whereas the most relevant changes occur in the VBM of systems with a small number of layers, leading to the hint that the composition mainly rules the surface properties rather than the number of layers.

We also note higher variations in the composition for the CBM than for the VBM. In other words, the range of values for the CBM for materials with different compositions is wider than that for the VBM as a result of the fact that their ionization potentials are higher than those of their work functions. Moreover, despite the fact that we were unable to identify any trend of the band edge positions with the group of the periodic table to which the metal belongs, the chalcogen species play an important role: Both the CBM and VBM values have a clear tendency to decrease for few-layer TMDs with heavier chalcogens in their compositions.

These results also demonstrate an interplay between the point inversion symmetry operation and the permanent electric fields. To explain, the breaking of the point inversion symmetry results in different charges flowing among the layers composing the few-layer TMD, which manifests itself as a polarization electric field perpendicular to the planar periodicity at the atomic scale and in surfaces with distinct work function (and their derivative properties) at the mesoscopic scale. These effects are analogous to those appearing in 2D Janus materials;^87^ however, here the effect is more intriguing as few-layer TMDs are composed of stacked layers with equal compositions. This effect was previously reported by Ferreira et al.^54^ for materials of a few layers of WSe_2_. However, our study expands the number of investigated materials in a systematic exploration.

The bottom panels of Figure 9 show the difference in the work function (Δ*V*) between the two sides, revealing the materials with inherent electric fields and serving as a measure of their intensity. Among the 17 investigated materials, only 5 (namely, FeS_2_, OsS_2_, OsSe_2_, RuS_2_, and RuTe_2_) preserve inherent electric fields, and among them, RuS_2_ stands out. The increase in Δ*V* with the number of layers is notable, which is consistent with an inherent electric field that extends to all layers. Also, such a monotonic increase demonstrates a difference between few-layer and monolayer TMDs as the prior systems allow for easier detection of this feature.

Moreover, this electric field decreases for heavier chalcogen species, a fact again ascribed to the electronegativity differences among S, Se, and Te. Comparing our higher simulated Δ*V* of 40 meV for RuS_2_ with *n* = 6 with other reported materials in the literature, we note that our few-layer TMDs have comparable (despite slightly lower) values than Janus monolayer materials^87^ and the results of Ferreira et al.^54^ for WSe_2_ bilayers.

This band edge analysis indicates that the number of layers can be used for tuning the band edge positions with respect to the vacuum level, which is a valuable degree of freedom to reach specific values demanded by specific catalytic activities. The band edge positions are especially relevant for catalytic properties, and our results indicate a high potential for few-layer TMDs for those purposes. Moreover, the presence of sulfur in the composition enhances the surface effects, which we ascribed to the higher sulfur electronegativity (Ξ_S_) to the detriment of Se and Te.

Moreover, in the context of 2D materials, graphene, gold, and platinum are often used as electrodes to inject a charge (electrons or holes) into layered devices. The alignment between the electrode work functions and the valence band maximum and conduction band minimum of the 2D materials (acting as the active region) significantly affects charge injection and influences device performance.^88^Figure 9 presents the experimental work functions for these commonly used electrodes, specifically for graphene,^82^ Au,^83^ and Pt.^84^ While graphene generally favors hole injection and Pt favors electron injection, the work function of gold occupies an intermediate energy position. Moreover, increasing the chalcogen atom size (S → Se → Te) in TMDs favors electron injection as the work function of the few-layer TMDs rises, resulting in a reduced tendency for hole injection in Te-based TMDs. Additionally, the number of layers influences these alignments: the CBM varies less significantly with the number of layers compared to the VBM, suggesting that layer count primarily tunes electron injection. However, the specific transition metal in the TMD significantly affects the CBM position, making it the key factor in regulating the hole injection. Thus, Figure 9 enables the selection of TMD compositions, layer numbers, and electrode materials to optimize electron or hole injection or to form tunneling barriers.

### 3.7 Role of the Number of Layers in Electric Polarization

**Figure 10:**
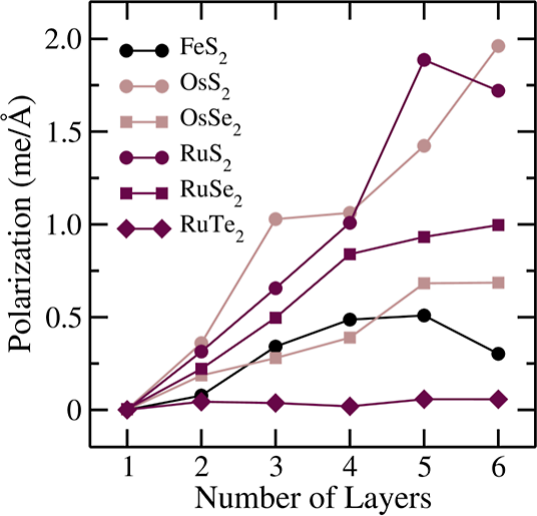
Polarization numerically evaluated as the electric dipole per area for the few layers presenting the symmetry-break-induced interlayer charge transfers.

We quantified the electric polarization induced by symmetry breaking by measuring the electric dipole per area, as shown in Figure 10. Notably, polarization arises only in systems with two layers and beyond, which aligns with the interlayer charge transfer mechanism. Furthermore, the polarization value increases with the number of layers, indicating that similar interlayer charge transfers occur in systems with a greater number of layers. Additionally, this polarization depends on the chalcogen; Te-based few-layers exhibit lower values than *S*-based ones. In other words, polarization tends to decrease with heavier chalcogens (i.e., it reduces in the order S → Se → Te). These values should be compared with other 2D systems, such as the group 4 monochalcogenides,^89^ which exhibit polarizations around 1.8 me/Å. In particular, there are quantitative similarities for few-layer TMDs with three or more layers.

## 4 Insights into the Unique Electronic Characteristics of Stacked Monolayers

Our work highlights two key aspects of few-layer TMDs: (i) the role of chalcogen species in determining structural, energetic, and electronic properties; and (ii) certain compositions exhibit unexpected features in their electronic structure. This section summarizes some aspects of these topics.

### 4.1 Role of the Chalcogen Species

**Figure 11:**
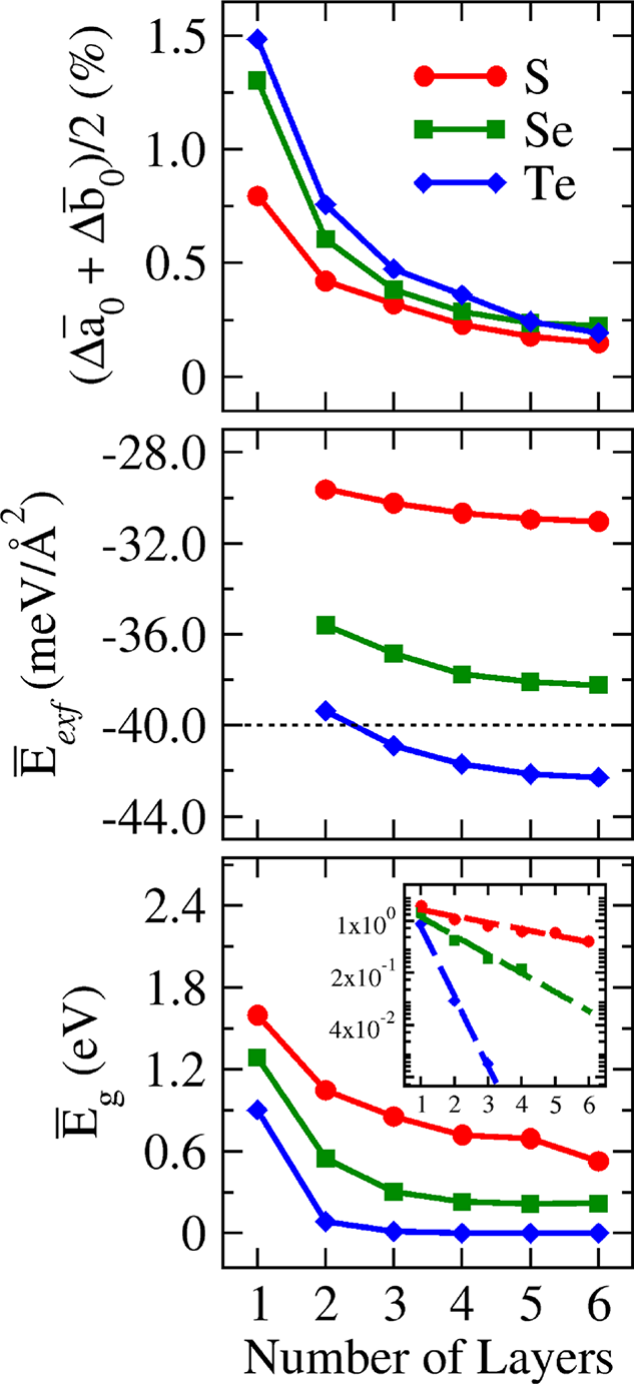
Average percentual deviations from bulk values with respect to the number of layers (*n*) for structural, energetic, and electronic parameters.

The presence of S, Se, or Te in the chalcogen composition significantly influences the properties of few-layer TMDs, as outlined in Figure 11. The percentage variations in the lattice parameters, averaged between the materials S, Se, and Te separately (top panel), demonstrate a stronger influence of the number of layers for Te-based compounds, followed by Se- and S-based ones, respectively. In line with these findings, the middle panel of Figure 11, which averages the exfoliation energy, indicates strong interlayer interactions for Te-based few-layer TMDs, followed by Se and S (consistent with the trend in the lattice parameter). This panel also marks the weak limit with a dashed line, revealing that only S-based compounds exhibit weak average exfoliation energies.

Consequently, our corrected band gap estimate (including SOC and hybrid *E*_xc_ corrections), depicted in the bottom panel of Figure 11, shows higher band gaps for S-based compounds compared to other chalcogen compositions. Here, it is worth stressing that several compositions undergo semiconductor-to-metal transitions, resulting in a band gap dependence on *n* that tends to zero with distinct closing rates for S-, Se-, and Te-based compositions. The inset of the bottom panel plots the band gaps against *n* using a logarithmic scale, revealing their exponential decrease (linear decrease on the logarithmic scale), where the role of the chalcogen species becomes more evident.

These comprehensive findings on structural, energetic, and electronic properties (illustrated in Figure 11) unequivocally demonstrate the decisive role of chalcogen species in few-layer TMD properties. The strength of the interlayer interactions, following the order S → Se → Te, significantly influences both the structural and electronic characteristics. To comprehend this, it is beneficial to separate the interlayer interaction into two components: (i) attractive interactions owing to van der Waals and charge-sharing forces between layers; and (ii) repulsive interactions encompassing intricate classical and quantum Coulomb repulsion, whereas the classical term arises from chalcogen atoms on monolayer surfaces. The higher electronegativity of chalcogens compared to those of transition-metal species displaces the electronic density from the inner regions to the surface, resulting in negatively charged surfaces. Thus, heavier chalcogens have lower electronegativity in comparison to lighter ones, resulting in surfaces with lower charges, which decreases the classical Coulomb repulsion and then enhances the interlayer interactions.

This insight underscores the importance of engineering chalcogen compositions, such as Janus structures and chalcogen mixtures (alloys), to tune the structural, energetic, and electronic properties of few-layer TMDs. Furthermore, because of sulfur’s higher electronegativity, its presence in compositions predominantly affects surface properties.

### 4.2 Unexpected Electronic Features

Layered materials have flexible electronic properties, in the sense that numerous degrees of freedom can be used to tune their electronic properties.^90^ Our investigated few-layer TMDs demonstrate intrinsic characteristics that result in unconventional properties compared to most 2D layered materials.

The most evident is the arising of polarization electric fields originating from asymmetric interlayer charge flow triggered by the lack of point inversion symmetry. However, here, it is interesting that this feature appears in the stacking of layers with equivalent chemical compositions and crystalline structures. This characteristic is present for FeS_2_, OsS_2_, OsSe_2_, RuS_2_, RuSe, and RuTe_2_, and polarization electric fields induce differences of 40 meV in six layers for RuS_2_, for example, a value comparable to other systems in which the polarization electric field was triggered by other mechanisms. This particular feature is of great relevance for catalytic applications because the work function and the ionization potential can be finely tuned by the number of layers. The presence of sulfur surface atoms enhances this effect as a result of their higher electronegativity among all of the considered chalcogens.

In general, it is expected that the SOC effects on the electronic structure depend only on the composition. However, the investigated few-layer TMDs reveal unexpected SOC effects that depend on the number of layers. There are materials that unexpectedly widen the band gap value as a result of spin–orbit coupling. This behavior is possible due to the low number of bands around the Fermi level, and then the SOC splittings mostly at the Γ point affect the entire low-energy electronic bands, resulting in a peculiar band gap that increases by more than 100 meV for FeTe_2_, OsTe_2_, and RuTe_2_. Moreover, the PBE + D3 approach fails to classify some materials as metals or semiconductors as in the examples of OsTe_2_ and PtSe_2_ with *n* = 3 and RuSe_2_ with *n* = 6. Thus, corrections due to SOC and self-interaction errors are necessary.

## 5 Conclusions

This study examines few-layer transition metal dichalcogenides characterized by chemical formulas MQ_2_, wherein M belongs to groups 8 and 10 of the periodic table and Q is represented by S, Se, or Te, with structure configurations extending up to six layers. The selection of crystalline structures for each composition is derived from previous investigations of these materials in both their bulk and monolayer manifestations.^37^ Optimized geometries are the result of computations within the DFT–PBE + D3 framework, while the electronic properties integrate corrections for spin–orbit couplings employing noncollinear spin calculations in conjunction with self-interaction errors addressed through the HSE06 hybrid exchange–correlation energy functional.

Our structural analysis demonstrates variations in the lattice parameters with changes in the number of layers up to 2.4% with respect to the bulk values. Here, the chalcogen species prove to be the most important factor. However, TMDs of group 10 change the crystalline phase and the lattice parameter for different chalcogen and transition metal species, while the structural properties of the TMDs of group 8 are almost insensitive to the choice of the transition metal species, becoming attractive for building commensurate heterostructures. None of the structures exhibit a magnetic moment. Although all group 10 TMDs are intrinsically nonmagnetic, group 8 TMDs lose their magnetism upon undergoing a phase transition from 1*T* to 1*T*′, with phase 1*T*′ being the ground state.

Moreover, the interlayer interaction evaluated from the exfoliation energies also demonstrates the relevant role of the chalcogen species. In this case, the decrease in electronegativity from S to Te results in lower surface charges and therefore stronger interlayer interactions as the Coulomb repulsion term (which opposes the attractive long-range terms) is reduced. Finally, we proceed to a deeper investigation of the electronic properties via band structure calculations, where the stronger interactions for heavier chalcogens result in lower band gaps, along with their higher closing rates.

The electronic properties demonstrate numerous peculiarities for some systems, such as an unexpected band gap increase driven by spin–orbit coupling for a few compositions, the arising of an intrinsic electric polarization increasing with the number of layers triggered by the break of point inversion symmetry, and semiconductor-to-metal transitions that, in some cases, occur by adding only one or two more layers to the monolayer. The presence of sulfur at the surface enhances the sensitivity of the surface properties, allowing the band edge positions to be tuned with the number of layers and the choice of transition-metal species. Thus, these few-layer TMDs are attractive for catalytic processes.

## Supporting Information Available

Specific details related to the VASP code; computational tests on the role of the dipole correction; and additional structural, energetic, and electronic properties for each of the 119 systems investigated (PDF)

## Abbreviations

DFT: density functional theory
VASP: Vienna ab initio simulation package
KS: Kohn–Sham
PAW: projector augmented-wave
GGA: generalized gradient approximation
PBE: Perdew–Burke–Ernzerhof
*E*_xc_: exchange-correlation energy
vdW: van der Waals
PBE + D3: Perdew–Burke–Ernzerhof plus D3 van der Waals correction
SOC: spin–orbit coupling
HSE06: Heyd–Scuseria–Ernzerhof
TMD: transition-metal dichalcogenide
VBM: valence band maximum
CBM: conduction band minimum.
