# Supplementary material for: Theoretical Investigation of Stacked Two-Dimensional Transition-Metal Dichalcogenide Materials: The Role of Chemical Species and Number of Monolayers
Source: ACS Omega. 2025 Feb 25;10(9):8922–34. doi: 10.1021/acsomega.4c05423 (PMC11904659; doi:10.1021/acsomega.4c05423)
Supplement: Supplementary file 1 — ao4c05423_si_001.pdf [file ao4c05423_si_001.pdf]

Supporting Information: Theoretical Investigation of Stacked Two-Dimensional Transition-Metal Dichalcogenide Materials: The Role of Chemical Species and Number of Monolayers.

Jean M. Bracht,† Mateus B. P. Querne,† Juarez L. F. Da Silva,‡ and Matheus P. Lima*,†

†*Department of Physics, Federal University of São Carlos,* 13565-905*, São Carlos, SP, Brazil.*

‡*São Carlos Institute of Chemistry, University of São Paulo, P.O. Box* 780, 13560- 970*, São Carlos, SP, Brazil.*

E-mail: mplima@df.ufscar.br

Supporting Information

Contents

1 Selected PAW Projectors. **S-2**

2 Selected Materials. **S-3**

2.1 Crystalline Phases and Selected Compositions. S-3

3 Computatinal Tests: Role of Dipole Corrections. **S-5**

3.1 Structural and Energetic Properties. S-5

3.2 Layer Resolved Local Density of States. S-15

3.3 Conclusions From the Tests on the Dipole Corrections. S-19

4 Results **S-20**

4.1 Lattice Parameters and Total Energies S-20

4.2 Point-Inversion Symmetry Analysis for Few-Layer Materials. S-29

4.3 Exfoliation, Adsorption and Relaxation Energies S-30

4.4 Band Structures. S-35

4.5 Band Gaps as a Function of the Number of Layers. S-43

4.6 Band Gap Corrections S-44

4.7 Electrostatic Potential Energy as a Function of the Number of Layers. S-48

4.8 Band Offset S-57

References **S-68**

1 Selected PAW Projectors.

We selected projector-augmented-wave (PAW) projectors with the `GW` variant and the lower value for `ENMAX` among them. Table S1 identifies the employed PAW projects for each atomic specie.

Table S1: Selected PAW projectors for each element. Projector name (title); creation date (date); number of valence electrons (Z_{val}); electronic valence distribution (valence); and maximum recommended cutoff energy (`ENMAX`). We obtained these projectrons from the potpaw_pbe_5.4 library.

Element	title	date	Z_{val}	valence	`ENMAX` (eV)
Fe	Fe_GW	31/03/2010	8	$3d^7 4s^1$	321.007
Ru	Ru_sv_GW	05/12/2013	16	$4s^2 4p^6 4d^8$	348.106
Os	Os_sv_GW	23/03/2010	16	$5s^2 5p^6 5d^8$	319.773
Ni	Ni_GW	31/03/2010	10	$3d^9 4s^1$	357.323
Pd	Pd_GW	06/03/2008	10	$4d^9 5s^1$	250.925
Pt	Pt_GW	10/03/2009	10	$5d^9 6s^1$	248.716
S	S_GW	19/03/2012	6	$3s^2 3p^4$	258.689
Se	Se_GW	20/03/2012	6	$4s^2 4p^4$	211.555
Te	Te_GW	22/03/2012	6	$5s^2 5p^4$	174.982

2 Selected Materials.

In this work, we investigate few layers of two-dimensional (2D) transition metal dichalcogenides (TMDs), motivated by an earlier work published by some of the authors of this manuscript[1]. This earlier work selected 17 materials from a theoretical investigation of their monolayers and van der Waals crystal bulks with density-functional theory (DFT) techniques, demonstrating their great potential for applications in technological fields. The present work aims to reveal two aspects of these materials: (i) the evolution of the structural, energetic, and electronic properties with the number of layers and (ii) the role of the chalcogen species (S, Se, or Te) on the electronic properties of these materials.

2.1 Crystalline Phases and Selected Compositions.

Each investigated material belongs to one of the four crystal phases shown in Figure S1, namely, 1T', PdS_2-type, Calaverite, or 1T. This figure shows examples of slabs containing four layers ($n = 4$). However, we also explore slabs with the number of layers ranging from $n = 1$ to 6, and compare their properties with those of van der Waals crystal bulks ($n \to \infty$).

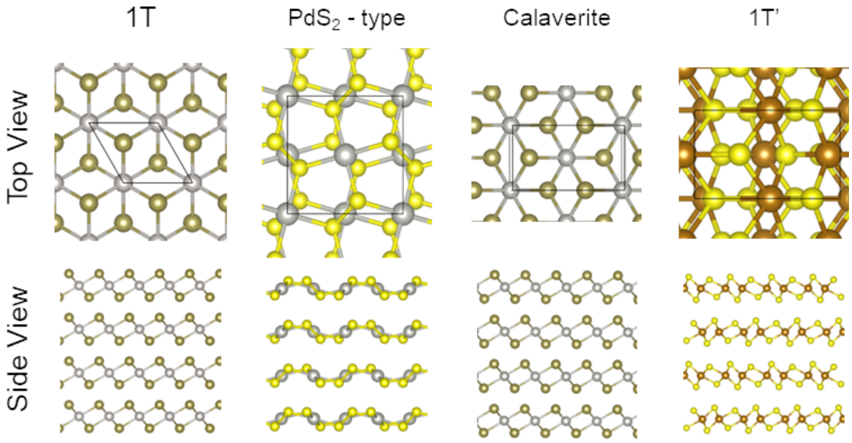

Figure S1: Ball and stick representation for the crystalline structures investigated in this work, namely, 1T', PdS_2-type, Calaverite and 1T. The top views show the unit cell as solid line, whereas the side view shows structures with four layers. In these particular structures, the compositions for the 1T', PdS_2-type, Calaverite and 1T crystalline structures are FeS_2, PdS_2, $PdTe_2$, and $PtTe_2$, respectively.

The investigated MQ_2 compositions have M = Fe, Ni, Os, Pd, Pt, or Ru; and Q = S, Se, or Te. In our previous work, a screening for lowest energy semiconductor MQ_2 2D TMDs considers various crystalline structures from explorations only of their monolayer ($n = 1$) and their van der Waals bulks ($n \to \infty$). However, the present work investigates a few layers of these materials in their lowest energy structure, determining the evolution of their properties with the number of layers. NiTe was not included due to its metallic behavior in both monolayer and van der Waals crystal forms. Figure S1 shows the crystalline structure for each composition, and Table S2 identifies the crystalline phase for each composition and the number of formula units per layer. These structures corroborate with data presented in the computational two-dimensional materials database (C2DB)[2,3].

Table S2: MQ_2 compositions (Composition), the respective crystalline structure (Structure) and the number of formulas units per layer ($\frac{f.u.}{layer}$).

Composition	Structure	$\frac{f.u.}{layer}$
FeS_2	1T'	2
$FeSe_2$	1T'	2
$FeTe_2$	1T'	2
RuS_2	1T'	2
$RuSe_2$	1T'	2
$RuTe_2$	1T'	2
NiS_2	1T	1
$NiSe_2$	1T	1
OsS_2	1T'	2
$OsSe_2$	1T'	2
$OsTe_2$	1T'	2
PdS_2	PdS_2-type	2
$PdSe_2$	1T	1
$PdTe_2$	Calaverite	2
PtS_2	Calaverite	2
$PtSe_2$	Calaverite	2
$PtTe_2$	1T	1

3 Computatinal Tests: Role of Dipole Corrections.

When there is a breaking of Point Inversion Symmetry (PIS) in a layered material, it implies that the charge distribution should not be symmetrical about the layers. This can result in the creation of polarization electric fields perpendicular to the planar periodicity directions. In this context, dipole corrections become crucial for evaluating the properties of two-dimensional materials, such as 2D TMDs, as they help correct numerical errors and improve the accuracy of results obtained by computational methods. This correction adds an opposite electric field to the polarization one to break the long-range behavior of the electrostatic interactions. It's worth noting that in materials that maintain PIS, this polarization effect may not be significant and thus does not need to be considered dipole corrections in the calculations.

3.1 Structural and Energetic Properties.

This section presents further details to determine the role of dipole correction in the structural and energetic properties by comparing results obtained from optimized few-layer 2D TMDs

performed with dipole corrections and without it. These effects on the structural properties were assessed through the lattice parameters a_0 and b_0, interlayer distances $d_{\text{interlayer}}$, and layer thickness, all of them in Å. Moreover, these effects on the energetic properties were evaluated through two parameters, namely, E_{B1} and E_{B2}, given by:

$$E_{B1} = \frac{E_{\text{monolayer}} - \frac{E_{\text{few layers}}}{N_{\text{few layers}}}}{A_{\text{few layers}}} \tag{1}$$

and

$$E_{B2} = \frac{E_{\text{few layers}} - E_{\text{bulk}} \cdot \frac{N_{\text{few layers}}}{N_{\text{bulk}}}}{A_{\text{few layers}}}. \tag{2}$$

E_{B1} corresponds to the adsorption energy, i.e., the total energy required to split all layers into single layers; whereas E_{B2} corresponds to the total energy required to remove a few layers containing n layers from the van der Waals crystal bulk. In the abover expression, $E_{\text{monolayer}}$ represent the total energy for opmized single-layer, E_{bulk} represent the total energy for the van der Waals crystal bulk, N_{bulk} is the number of layers for the van der Waals crystal bulk, $N_{\text{few layers}}$ is the number of layer for the few-layer systems, and $A_{\text{few layers}}$ is the area of the unit cell for the few-layer systems. Figures S2 to S18 show these comparisons for all compositions using the results from Tables S3 to S10.

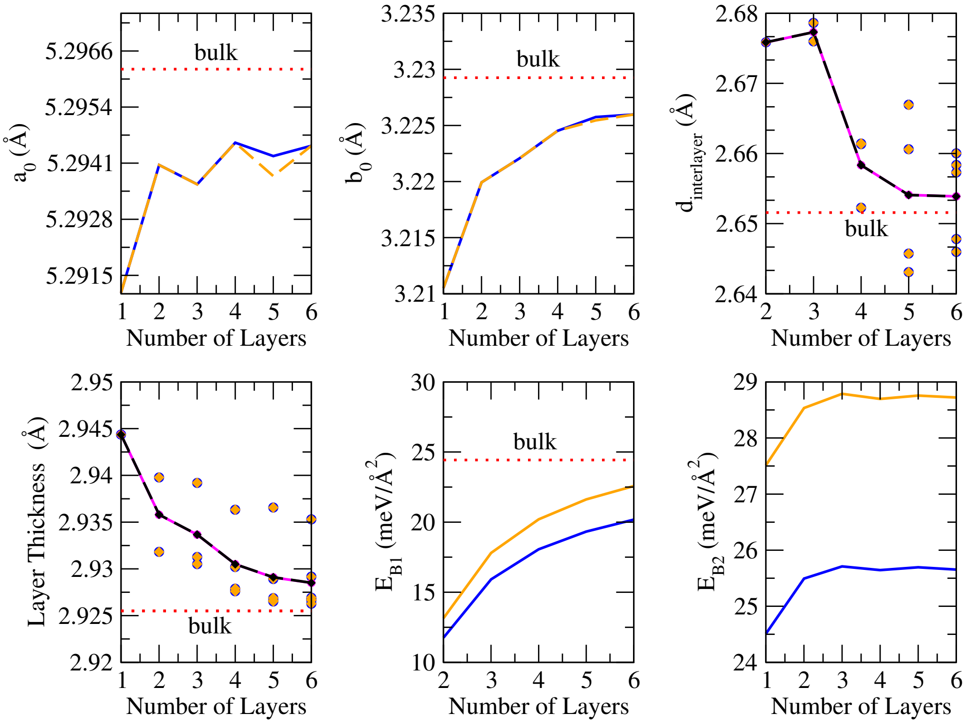

Figure S2: Structural and energetic properties of few-layer FeS_2 with dipole correction (orange and black colors) and without it (blue and magenta colors) are shown. The red dotted lines represent the bulk values, serving as a reference. The panels present planar lattice parameters a_0 and b_0, interlayer distances $d_{\text{interlayer}}$, layer thickness, and the two investigated binding energies E_{B_1} and E_{B_2}.

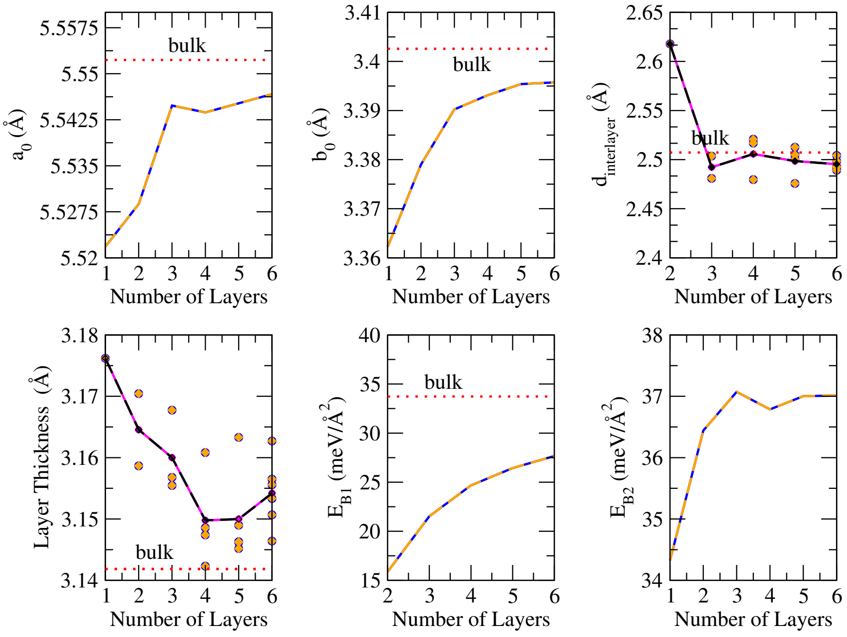

Figure S3: Structural and energetic properties of few-layer $FeSe_2$ with dipole correction (orange and black colors) and without it (blue and magenta colors) are shown. The red dotted lines represent the bulk values, serving as a reference. The panels present planar lattice parameters a_0 and b_0, interlayer distances $d_{\text{interlayer}}$, layer thickness, and the two investigated binding energies E_{B_1} and E_{B_2}.

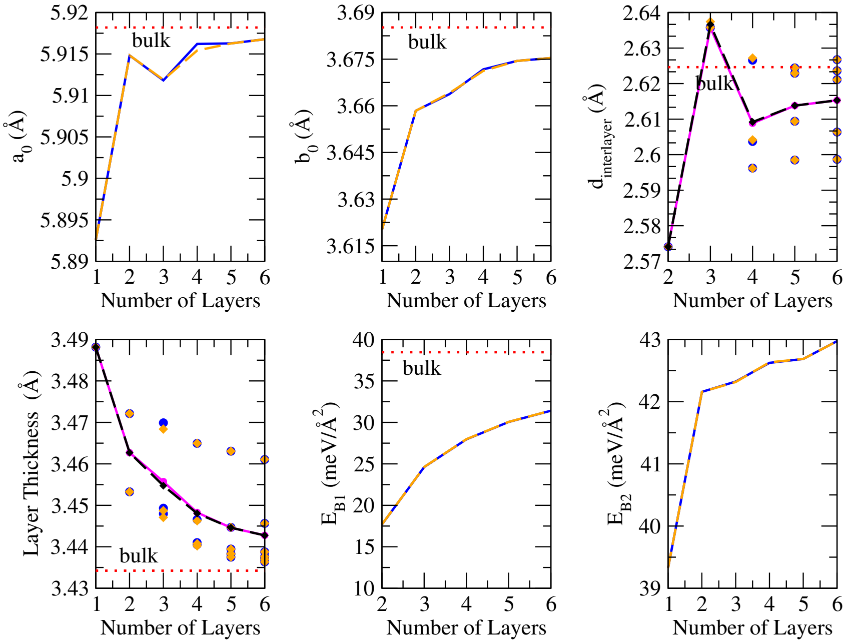

Figure S4: Structural and energetic properties of few-layer $FeTe_2$ with dipole correction (orange and black colors) and without it (blue and magenta colors) are shown. The red dotted lines represent the bulk values, serving as a reference. The panels present planar lattice parameters a_0 and b_0, interlayer distances $d_{\text{interlayer}}$, layer thickness, and the two investigated binding energies E_{B_1} and E_{B_2}.

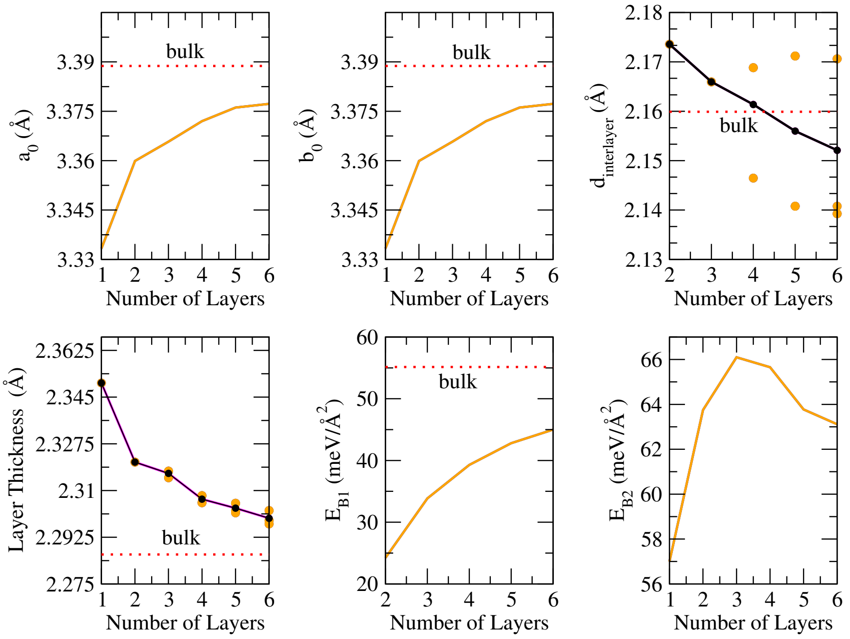

Figure S5: Structural and energetic properties of few-layer NiS_2 with dipole correction (orange and black colors) and without it (blue and magenta colors) are shown. The red dotted lines represent the bulk values, serving as a reference. The panels present planar lattice parameters a_0 and b_0, interlayer distances $d_{\text{interlayer}}$, layer thickness, and the two investigated binding energies E_{B_1} and E_{B_2}.

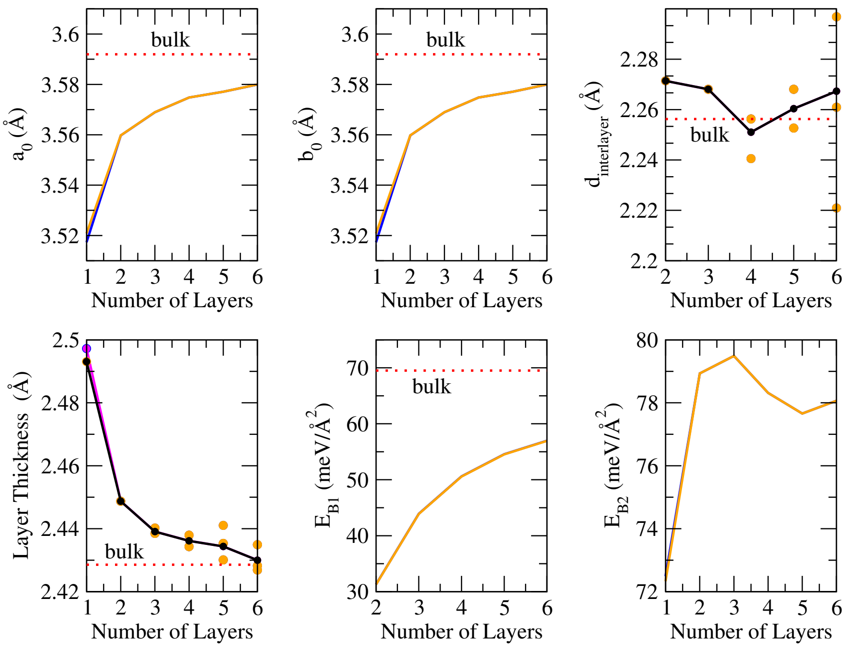

Figure S6: Structural and energetic properties of few-layer $NiSe_2$ with dipole correction (orange and black colors) and without it (blue and magenta colors) are shown. The red dotted lines represent the bulk values, serving as a reference. The panels present planar lattice parameters a_0 and b_0, interlayer distances $d_{\text{interlayer}}$, layer thickness, and the two investigated binding energies E_{B_1} and E_{B_2}.

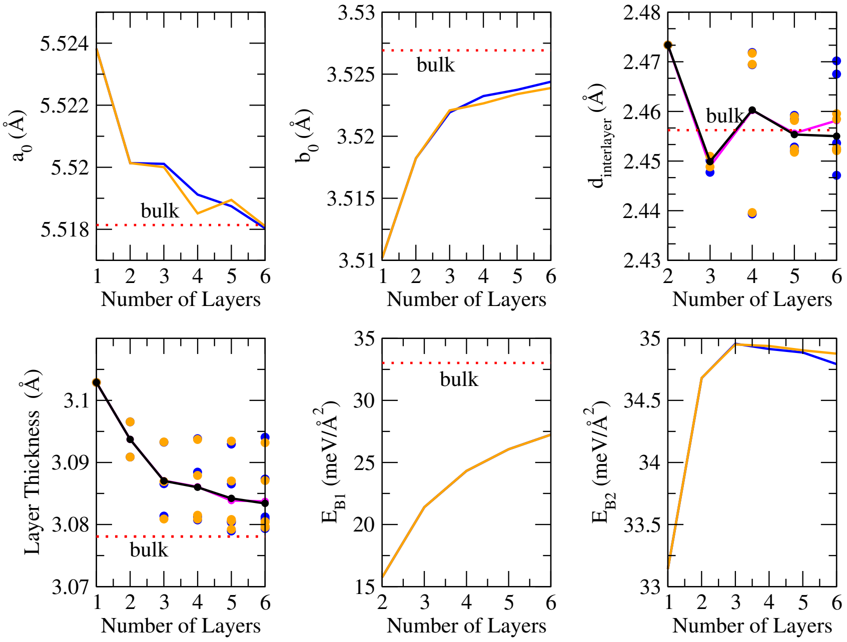

Figure S7: Structural and energetic properties of few-layer OsS_2 with dipole correction (orange and black colors) and without it (blue and magenta colors) are shown. The red dotted lines represent the bulk values, serving as a reference. The panels present planar lattice parameters a_0 and b_0, interlayer distances $d_{\text{interlayer}}$, layer thickness, and the two investigated binding energies E_{B_1} and E_{B_2}.

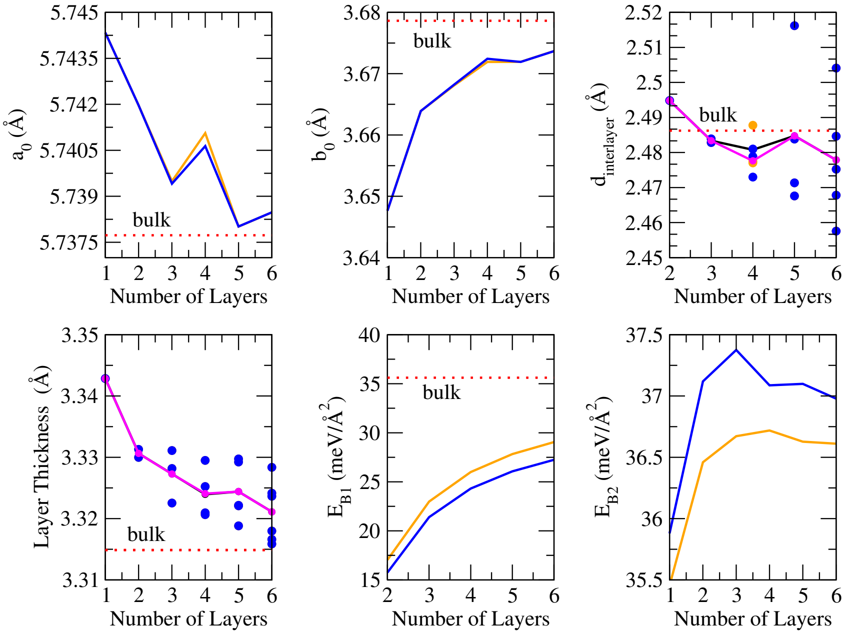

Figure S8: Structural and energetic properties of few-layer $OsSe_2$ with dipole correction (orange and black colors) and without it (blue and magenta colors) are shown. The red dotted lines represent the bulk values, serving as a reference. The panels present planar lattice parameters a_0 and b_0, interlayer distances $d_{\text{interlayer}}$, layer thickness, and the two investigated binding energies E_{B_1} and E_{B_2}.

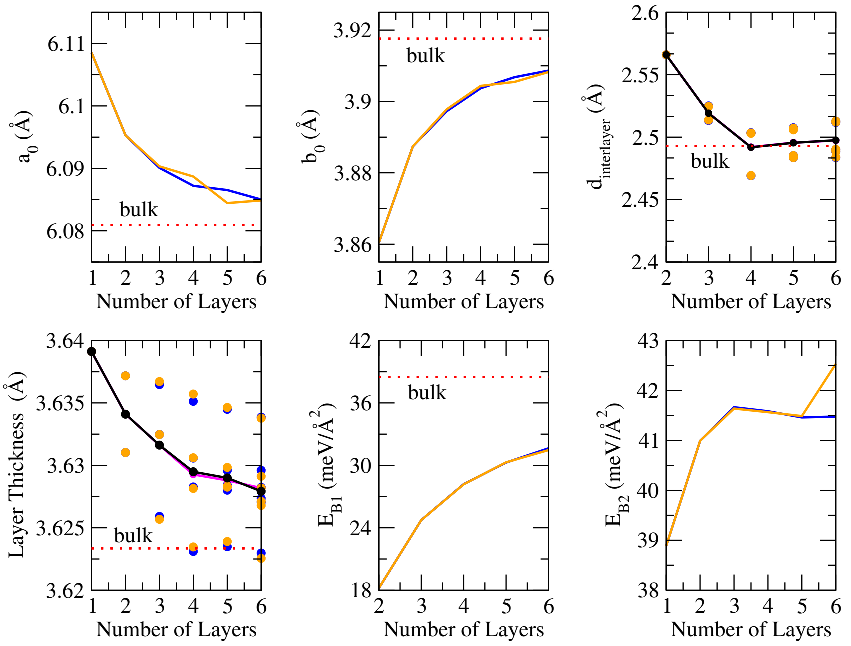

Figure S9: Structural and energetic properties of few-layer $OsTe_2$ with dipole correction (orange and black colors) and without it (blue and magenta colors) are shown. The red dotted lines represent the bulk values, serving as a reference. The panels present planar lattice parameters a_0 and b_0, interlayer distances $d_{\text{interlayer}}$, layer thickness, and the two investigated binding energies E_{B_1} and E_{B_2}.

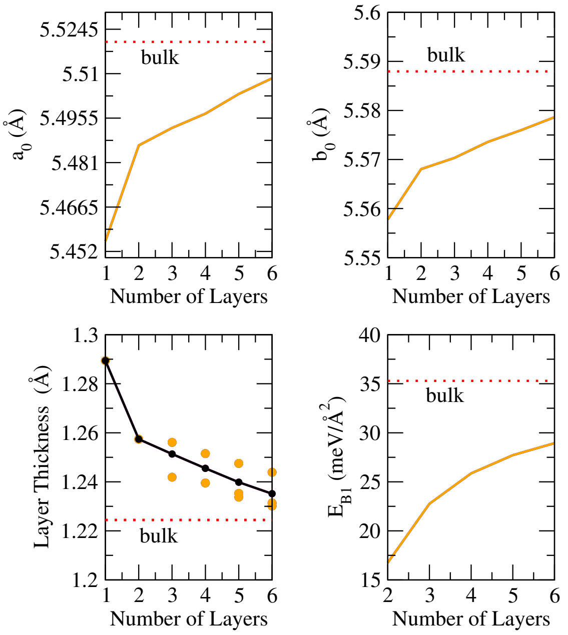

Figure S10: Structural and energetic properties of few-layer PdS_2 with dipole correction (orange and black colors) and without it (blue and magenta colors) are shown. The red dotted lines represent the bulk values, serving as a reference. The panels present planar lattice parameters a_0 and b_0, interlayer distances $d_{\text{interlayer}}$, layer thickness, and the two investigated binding energies E_{B_1} and E_{B_2}.

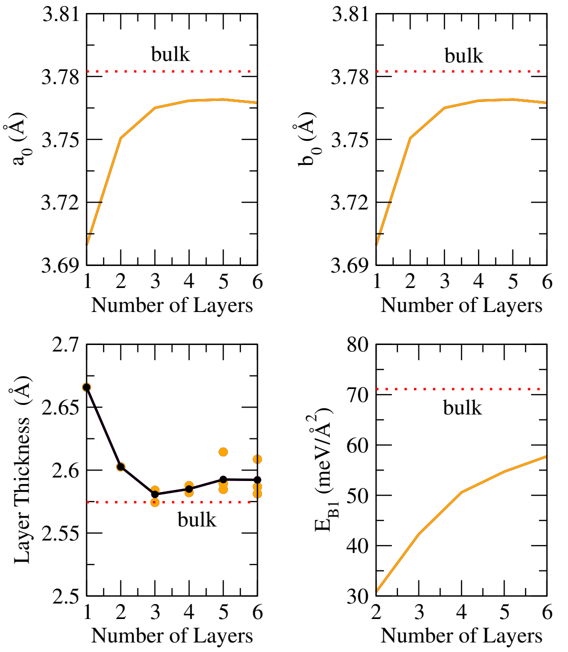

Figure S11: Structural and energetic properties of few-layer $PdSe_2$ with dipole correction (orange and black colors) and without it (blue and magenta colors) are shown. The red dotted lines represent the bulk values, serving as a reference. The panels present planar lattice parameters a_0 and b_0, interlayer distances $d_{\text{interlayer}}$, layer thickness, and the two investigated binding energies E_{B_1} and E_{B_2}.

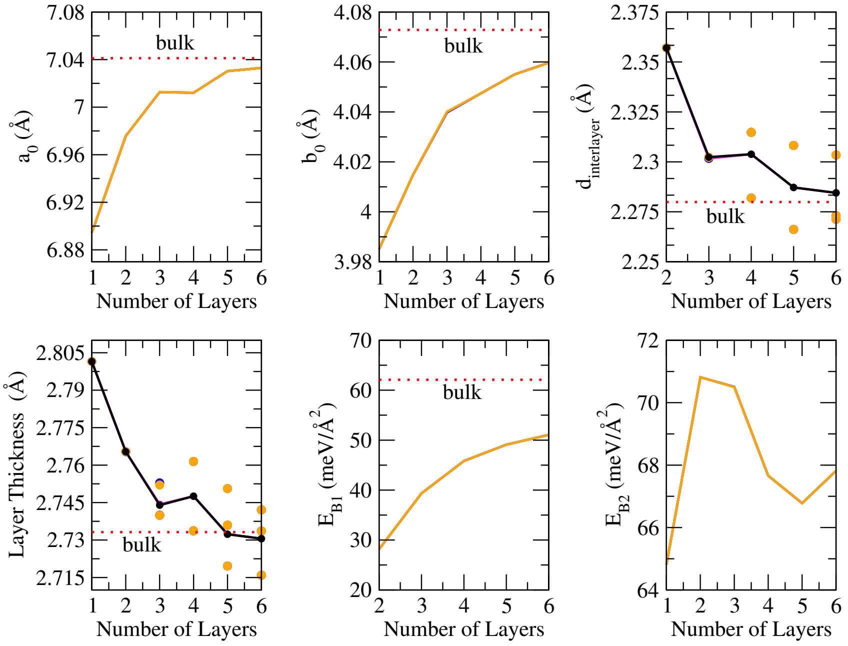

Figure S12: Structural and energetic properties of few-layer $PdTe_2$ with dipole correction (orange and black colors) and without it (blue and magenta colors) are shown. The red dotted lines represent the bulk values, serving as a reference. The panels present planar lattice parameters a_0 and b_0, interlayer distances $d_{\text{interlayer}}$, layer thickness, and the two investigated binding energies E_{B_1} and E_{B_2}.

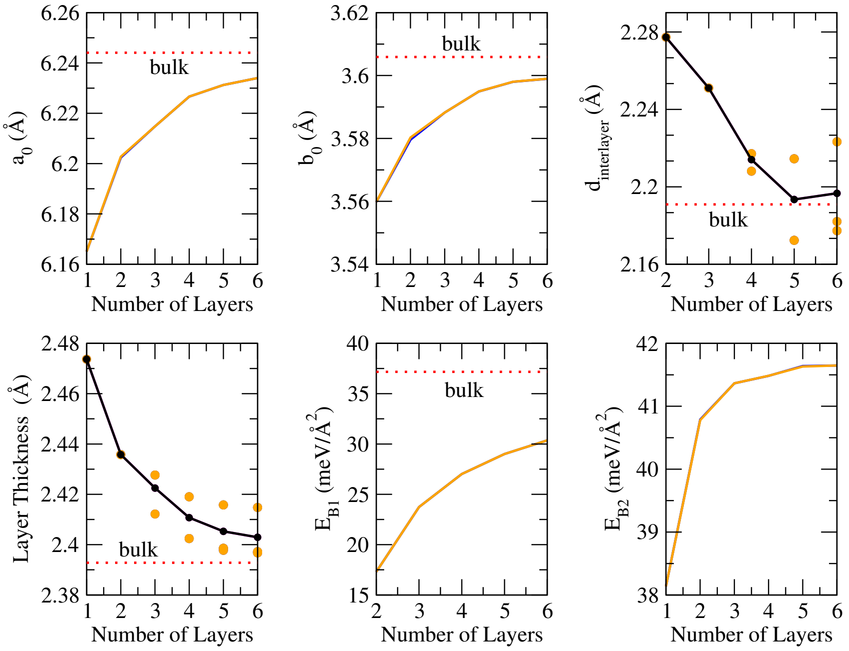

Figure S13: Structural and energetic properties of few-layer PtS_2 with dipole correction (orange and black colors) and without it (blue and magenta colors) are shown. The red dotted lines represent the bulk values, serving as a reference. The panels present planar lattice parameters a_0 and b_0, interlayer distances $d_{\text{interlayer}}$, layer thickness, and the two investigated binding energies E_{B_1} and E_{B_2}.

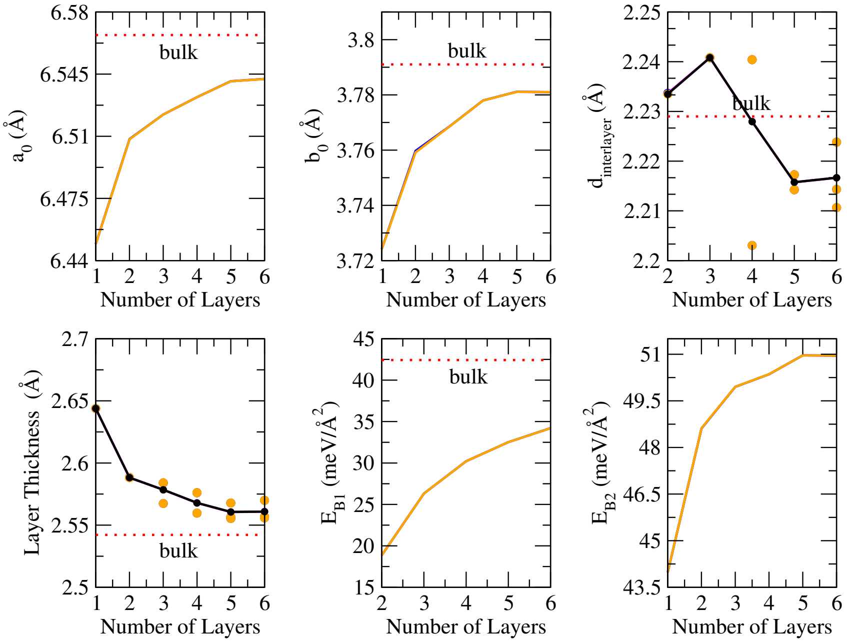

Figure S14: Structural and energetic properties of few-layer $PtSe_2$ with dipole correction (orange and black colors) and without it (blue and magenta colors) are shown. The red dotted lines represent the bulk values, serving as a reference. The panels present planar lattice parameters a_0 and b_0, interlayer distances $d_{\text{interlayer}}$, layer thickness, and the two investigated binding energies E_{B_1} and E_{B_2}.

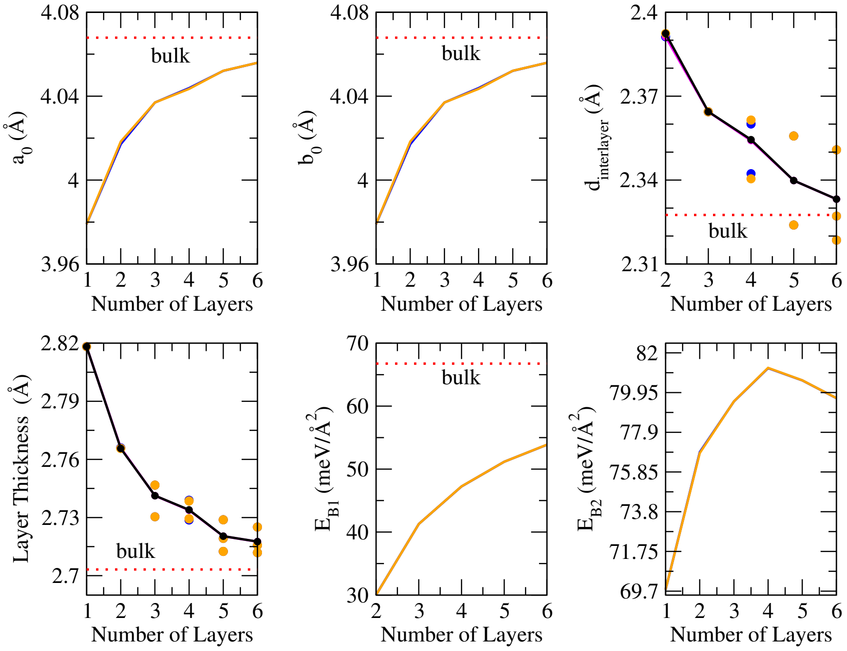

Figure S15: Structural and energetic properties of few-layer $PtTe_2$ with dipole correction (orange and black colors) and without it (blue and magenta colors) are shown. The red dotted lines represent the bulk values, serving as a reference. The panels present planar lattice parameters a_0 and b_0, interlayer distances $d_{\text{interlayer}}$, layer thickness, and the two investigated binding energies E_{B_1} and E_{B_2}.

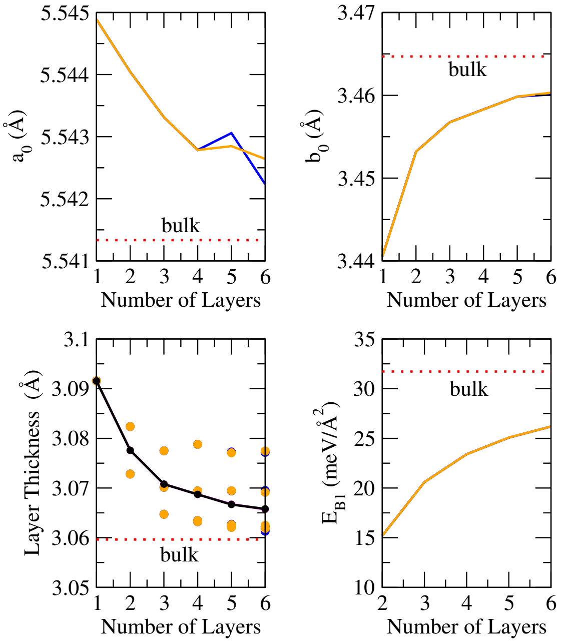

Figure S16: Structural and energetic properties of few-layer RuS_2 with dipole correction (orange and black colors) and without it (blue and magenta colors) are shown. The red dotted lines represent the bulk values, serving as a reference. The panels present planar lattice parameters a_0 and b_0, interlayer distances $d_{\text{interlayer}}$, layer thickness, and the two investigated binding energies E_{B_1} and E_{B_2}.

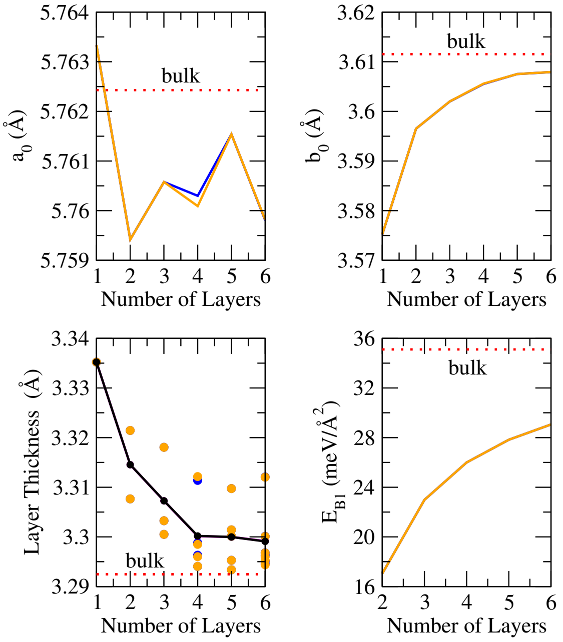

Figure S17: Structural and energetic properties of few-layer $RuSe_2$ with dipole correction (orange and black colors) and without it (blue and magenta colors) are shown. The red dotted lines represent the bulk values, serving as a reference. The panels present planar lattice parameters a_0 and b_0, interlayer distances $d_{\text{interlayer}}$, layer thickness, and the two investigated binding energies E_{B_1} and E_{B_2}.

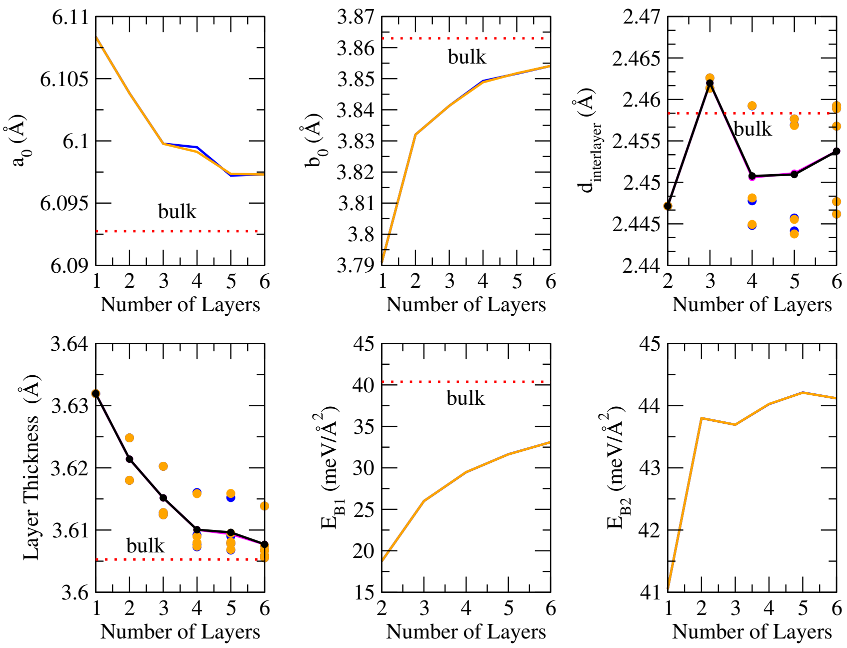

Figure S18: Structural and energetic properties of few-layer $RuTe_2$ with dipole correction (orange and black colors) and without it (blue and magenta colors) are shown. The red dotted lines represent the bulk values, serving as a reference. The panels present planar lattice parameters a_0 and b_0, interlayer distances $d_{\text{interlayer}}$, layer thickness, and the two investigated binding energies E_{B_1} and E_{B_2}.

From these Figures (S2 to S18), it is possible to conclude that some systems present good agreement for calculations with and without dipole corrections. However, specifically FeS_2, $OsSe_2$, $OsTe_2$ with $n = 6$, RuS_2 with $n = 5$ and 6, $RuSe_2$, and $RuTe_2$ show disagreements between the geometrical and energetic properties in calculations with and without dipole corrections.

3.2 Layer Resolved Local Density of States.

This section present our tests on the influence of dipole corrections on the electronic states via the graphing of Local Density of States (LDOS). We choose two systems: (i) the RuS_2, as we note severous influence of the polarization electric field on the geometrical and electronic properties, as noted in Figure S17; and (ii) the NiS_2, which serve as a control for our tests as this system preserve the PIS.

In the tests presented here, we optimize the system with dipole corrections and without them. Below, we present LDOS evaluated from the optimized geometries in distinct situations, summing the LDOS for each layer separately in each panel. Figure S19 evaluates the LDOS

from the geometry optimized without dipole corrections using a Hamiltonian without dipole corrections. It can be noted that there is a separation in energy of the states for each layer. We interpret this as the effect of the polarization electric field of the image layer acting on the investigated layer. As the electric field generates a ramp in the Hartree potential, states for layers at different z coordinates undergo distinct shifts due to this Hartree potential.

We also evaluate the LDOS from this same geometry; however, we turn on the dipole correction only in the evaluation in the Hamiltonian used to evaluate the LDOS, shown in Figure S20. There are notable changes in the shape of the LDOS. Here, the system acquires metallic behavior, which is intriguing as the van der Waals crystal bulk has a finite band gap (semiconducting behavior). Moreover, as the dipole correction blocks the long-range electric field, the states for each layer are degenerated in energy. However, since this geometry is frozen at atomic positions obtained without dipole corrections, these calculations may have errors.

Finally, we evaluate the LDOS from a geometry optimized with dipole corrections, and we also include such a correction in the Hamiltonian used to evaluate it, shown in Figure S21. Now, we observe the expected semiconductor behavior. Thus, these tests on the RuS_2 structure demonstrate the necessity to include dipole corrections in the evaluation of both the geometry optimization and the evaluation of its local density of states.

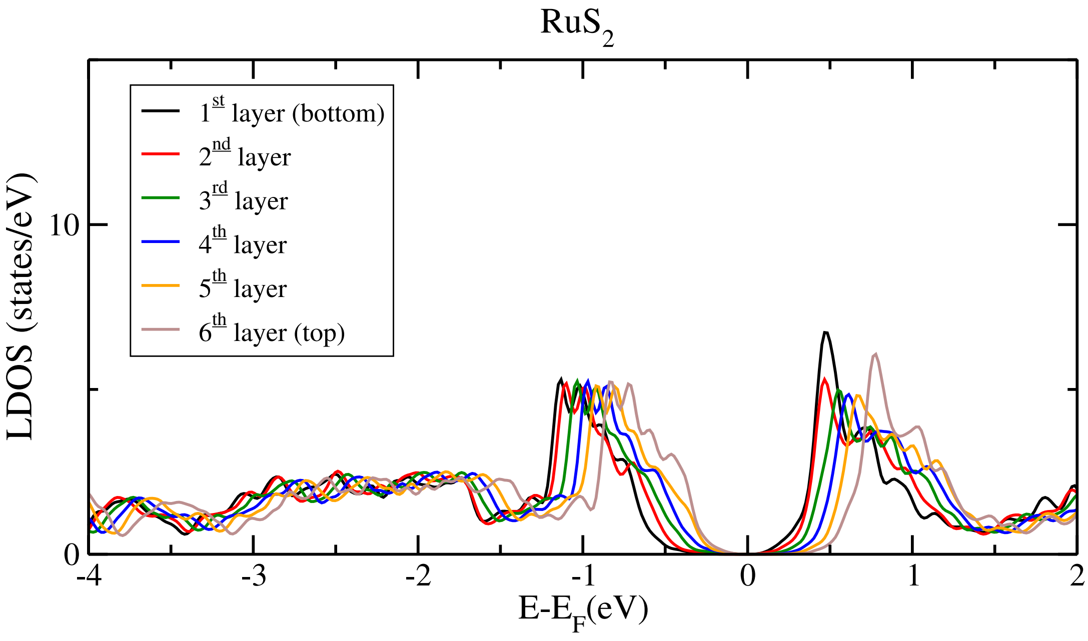

Figure S19: Local density of states obtained without dipole corrections for RuS_2 with 6 layers, and the atomic positions obtained form an geometry optimization carried without dipole corrections.

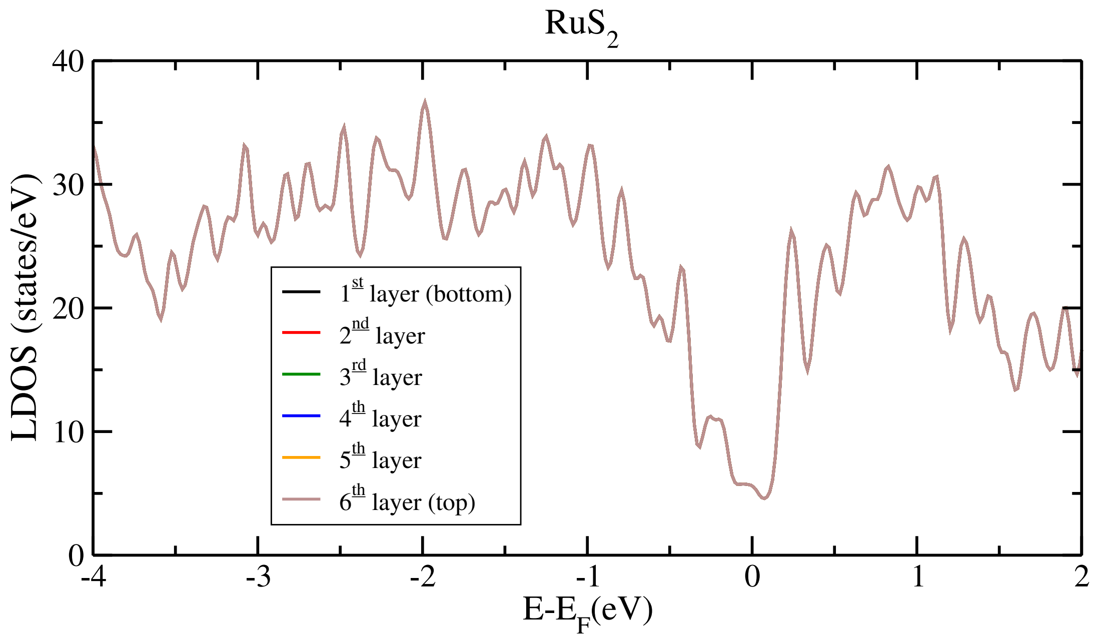

Figure S20: Local density of states obtained with the inclusion of dipole corrections in the electronic Hamiltonian for RuS_2 with 6 layers; however, the atomic positions were obtained from an geometry optimization carried without dipole corrections.

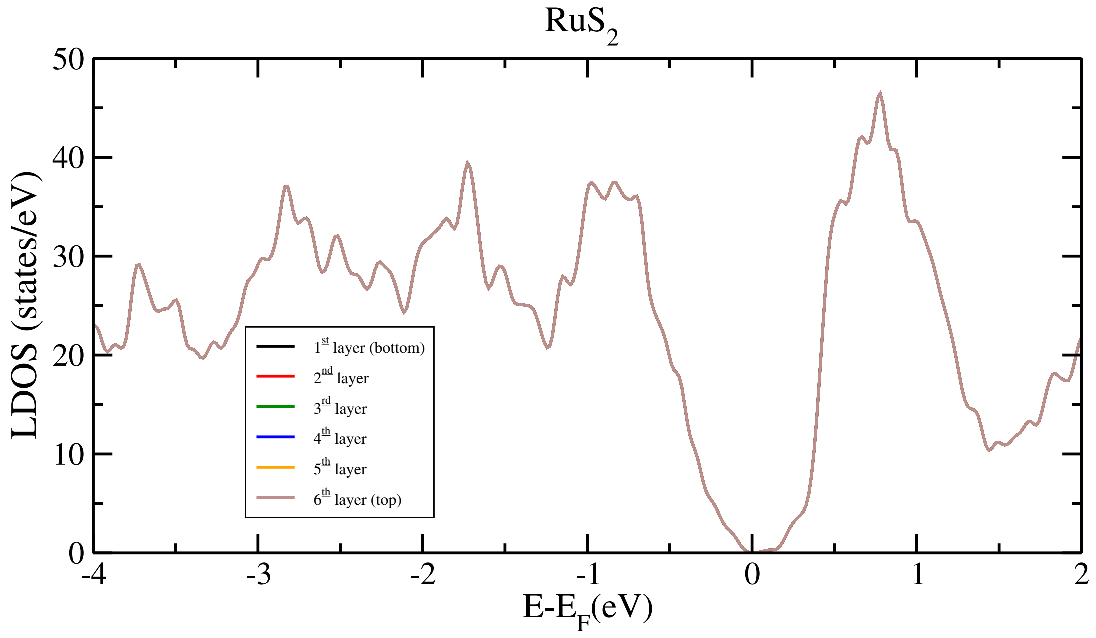

Figure S21: Full dipole corrected calculations. Local density of states obtained with dipole corrections for RuS_2 with 6 layers, from an structure previous relaxed with the inclusion of dipole corrections.

We perform exactly the same tests for NiS_2, a system with PIS, shown in Figures S22, S23, and S24. As noted, all approaches used for evaluating the LDOS result in LDOS with the same

shape, i.e., the dipole correction do not make effect in the DOS and geometry of NiS_2. One should expect this result, as polarization electrif fields do not arise in NiS_2.

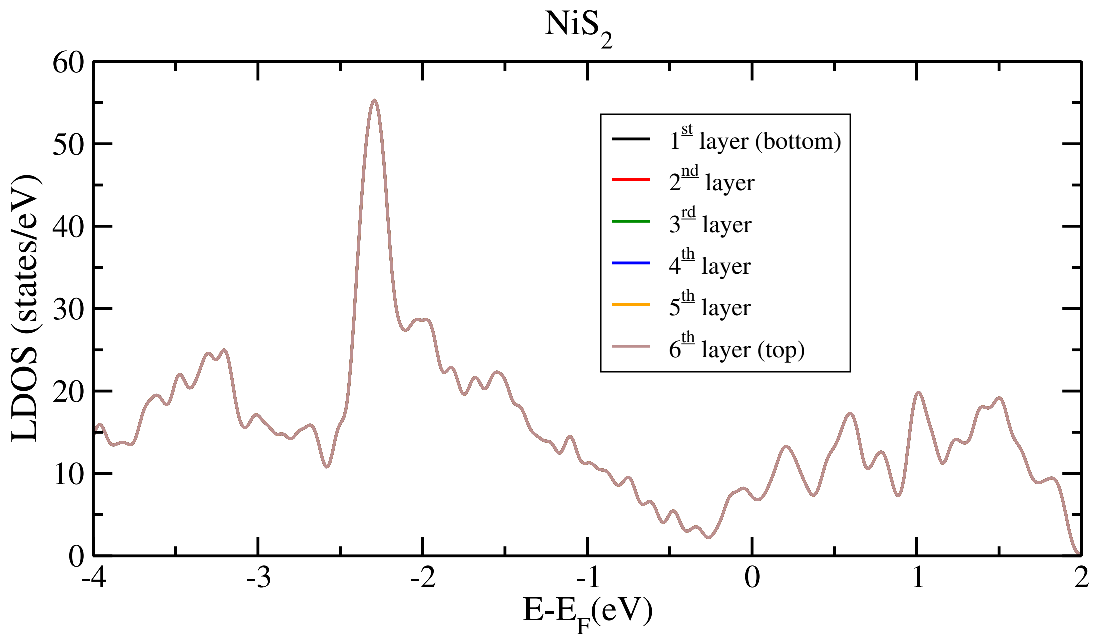

Figure S22: Local Density of States obtained without dipole corrections for NiS_2 with 6 layers. This structure was optimized with PBE+D3 functional without dipole corrections.

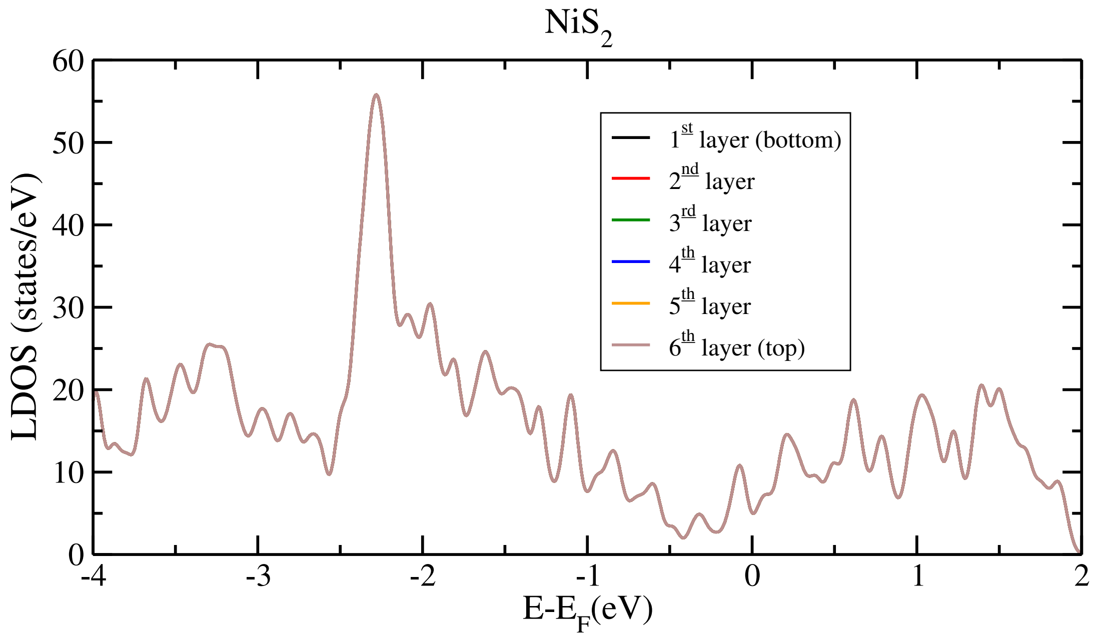

Figure S23: Local Density of States obtained with dipole corrections for NiS_2 with 6 layers. This structure was optimized with PBE+D3 functional without dipole corrections.

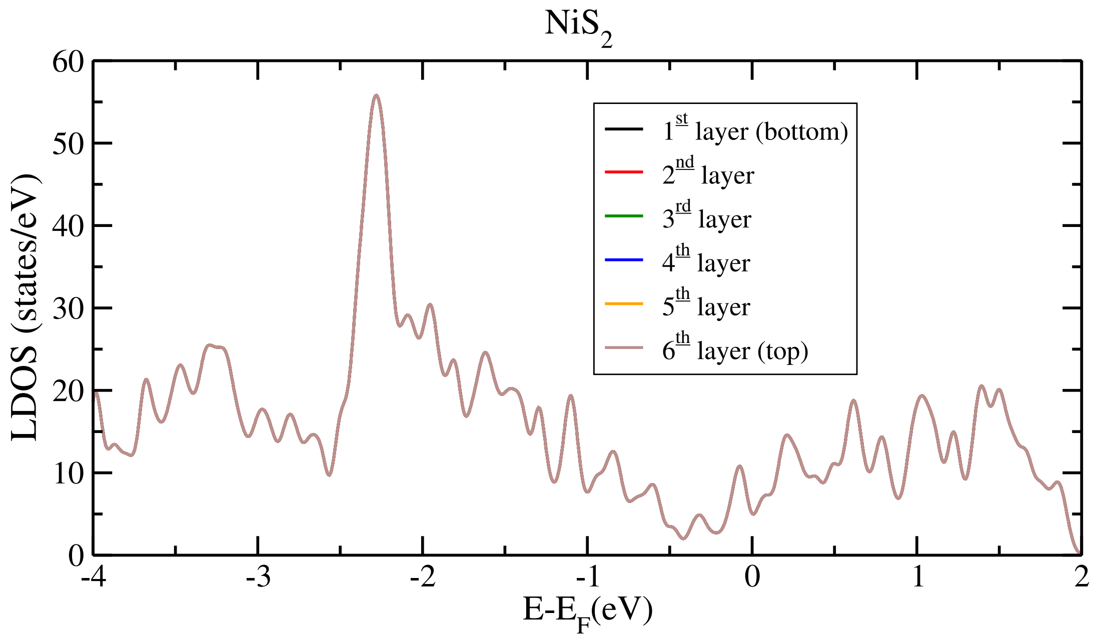

Figure S24: Local Density of States obtained with dipole corrections for NiS_2 with 6 layers. This structure was optimized with PBE+D3 functional with dipole corrections.

3.3 Conclusions From the Tests on the Dipole Corrections.

We ascribe such disagreements between the structural, energetic and electronic properties of calculations with and without the inclusion of dipole corrections to spurious interactions between the periodic images of the layers introduced from polarization electric fields. Thus, we include dipole correction for the geometry optimizations and to evaluate the energetic properties of all simulations shown in the main manuscript.

4 Results

4.1 Lattice Parameters and Total Energies

Table S3: Lattice Parameters for the 2D Crystal Structures (van der Waals crystals) obtained from our optimized structures (a_0, b_0 and c_0) compared to Reference values extracted from Ref.[1], a'_0, b'_0 and c'_0. Table also present total energy extracted from our simulations.

Composition	a_0 (Å)	a'_0 (Å)	b_0 (Å)	b'_0 (Å)	c_0 (Å)	c'_0 (Å)	E_{tot} (eV)
FeS_2	5.30	5.29	3.23	3.23	11.15	11.19	−72.649257
$FeSe_2$	5.55	5.56	3.40	3.41	11.30	11.28	−67.724029
$FeTe_2$	5.92	5.92	3.69	3.69	12.12	12.12	−62.953334
OsS_2	5.52	5.52	3.53	3.53	11.07	11.07	−88.728927
$OsSe_2$	5.74	5.74	3.68	3.68	11.60	11.58	−83.072653
$OsTe_2$	6.08	6.08	3.92	3.92	12.23	12.23	−78.623024
RuS_2	5.54	5.54	3.46	3.46	11.00	11.00	−76.255838
$RuSe_2$	5.76	5.76	3.61	3.61	11.52	11.50	−71.343568
$RuTe_2$	6.09	6.09	3.86	3.86	12.13	12.13	−67.520637
NiS_2	3.39	3.39	3.39	3.39	4.44	4.44	−15.144800
$NiSe_2$	3.59	3.59	3.59	3.59	4.68	4.68	−14.195620
PdS_2	5.52	5.52	5.59	5.59	7.36	7.46	−60.574487
$PdSe_2$	3.78	3.79	3.78	3.79	4.69	4.67	−14.158367
$PdTe_2$	7.04	7.04	4.07	4.08	5.01	5.02	−27.284751
PtS_2	6.24	6.25	3.61	3.61	4.58	4.59	−32.758880
$PtSe_2$	6.57	6.57	3.79	3.79	4.77	4.75	−30.847610
$PtTe_2$	4.07	4.07	4.07	4.07	5.03	5.03	−14.819852

Table S4: Lattice Parameters for the 2D Crystal Structures (van der Waals crystals) obtained from our optimized structures (α_0, β_0 and γ_0) compared to Reference values extracted from Ref.[1], α_0', β_0' and γ_0'.

Composition	α_0 (°)	α_0' (°)	β_0 (°)	β_0' (°)	γ_0 (°)	γ_0' (°)
FeS_2	90.0	90.0	90.0	90.0	90.0	90.0
$FeSe_2$	90.0	90.0	90.0	90.0	90.0	90.0
$FeTe_2$	90.0	90.0	90.0	90.0	90.0	90.0
OsS_2	90.0	90.0	90.0	90.0	90.0	90.0
$OsSe_2$	90.0	90.0	90.0	90.0	90.0	90.0
$OsTe_2$	90.0	90.0	90.0	90.0	90.0	90.0
RuS_2	90.0	90.0	90.0	90.0	90.0	90.0
$RuSe_2$	90.0	90.0	90.0	90.0	90.0	90.0
$RuTe_2$	90.0	90.0	90.0	90.0	90.0	90.0
NiS_2	90.0	90.0	90.0	90.0	120.0	120.0
$NiSe_2$	90.0	90.0	90.0	90.0	120.0	120.0
PdS_2	90.0	90.0	90.0	90.0	90.0	90.0
$PdSe_2$	90.0	90.0	90.0	90.0	120.0	120.0
$PdTe_2$	90.0	90.0	89.7	89.5	90.0	90.0
PtS_2	90.0	90.0	90.0	90.0	90.0	90.0
$PtSe_2$	90.0	90.0	90.2	89.9	90.0	90.0
$PtTe_2$	90.0	90.0	90.0	90.0	120.0	120.0

Table S5: Monolayer Lattice Parameters obtained from relaxed 2D crystal structures, where a_0,b_0 and c_0 (all values in Angstroms) are the modules of unit cell vectors a, b and c. The total energy (in electronvolts) and α_0, β_0 and γ_0 (all values in degrees) are the angles between unit cell vectors a and b, b and c, a and c, respectively. Also, we have MQ_2 compositions (composition).

Composition	a_0 (Å)	b_0 (Å)	c_0 (Å)	α_0 (°)	β_0 (°)	γ_0 (°)	E_{tot} (eV)
FeS_2	5.29	3.21	17.92	90.0	90.0	90.0	−35.906811
$FeSe_2$	5.52	3.36	18.14	90.0	90.0	90.0	−33.292367
$FeTe_2$	5.89	3.62	18.43	90.0	90.0	90.0	−30.726678
RuS_2	5.54	3.44	18.06	90.0	90.0	90.0	−37.583456
$RuSe_2$	5.76	3.58	18.29	90.0	90.0	90.0	−35.018637
$RuTe_2$	6.11	3.79	18.61	90.0	90.0	90.0	−32.910578
OsS_2	5.52	3.51	18.08	90.0	90.0	90.0	−43.789978
$OsSe_2$	5.74	3.65	18.31	90.0	90.0	90.0	−40.864229
$OsTe_2$	6.11	3.86	18.62	90.0	90.0	90.0	−38.491721
NiS_2	3.33	3.33	17.29	90.0	90.0	120.0	−14.777136
$NiSe_2$	3.52	3.52	17.43	90.0	90.0	120.0	−13.675026
PdS_2	5.46	5.56	16.22	90.0	90.0	90.0	−29.313950
$PdSe_2$	3.70	3.70	17.57	90.0	90.0	120.0	−13.567794
$PdTe_2$	6.89	3.99	17.74	90.0	89.4	90.0	−25.692943
PtS_2	6.17	3.56	17.39	90.0	90.1	90.0	−32.010652
$PtSe_2$	6.45	3.72	17.55	90.0	90.2	90.0	−29.903285
$PtTe_2$	3.98	3.98	17.70	90.0	90.0	120.0	−14.178477

Table S6: Bilayer Lattice Parameters obtained from relaxed 2D crystal structures, where a_0,b_0 and c_0 (all values in Angstroms) are the modules of unit cell vectors a, b and c. The total energy (in electronvolts) and α_0, β_0 and γ_0 (all values in degrees) are the angles between unit cell vectors a and b, b and c, a and c, respectively. Also, we have MQ_2 compositions (composition).

Composition	a_0 (Å)	b_0 (Å)	c_0 (Å)	α_0 (°)	β_0 (°)	γ_0 (°)	E_{tot} (eV)
FeS_2	5.29	3.22	23.50	90.0	90.0	90.0	−72.214409
$FeSe_2$	5.53	3.38	23.79	90.0	90.0	90.0	−67.115405
$FeTe_2$	5.91	3.66	24.49	90.0	90.0	90.0	−62.137805
RuS_2	5.54	3.45	23.56	90.0	90.0	90.0	−75.686886
$RuSe_2$	5.76	3.60	24.04	90.0	90.0	90.0	−70.668401
$RuTe_2$	6.10	3.83	24.67	90.0	90.0	90.0	−66.604786
OsS_2	5.52	3.52	23.61	90.0	90.0	90.0	−88.126780
$OsSe_2$	5.74	3.66	24.12	90.0	90.0	90.0	−82.374528
$OsTe_2$	6.10	3.89	24.74	90.0	90.0	90.0	−77.754667
NiS_2	3.36	3.36	21.73	90.0	90.0	120.0	−29.871747
$NiSe_2$	3.56	3.56	22.11	90.0	90.0	120.0	−27.810483
PdS_2	5.49	5.57	19.91	90.0	90.0	90.0	−59.543447
$PdSe_2$	3.75	3.75	22.26	90.0	90.0	120.0	−27.638277
$PdTe_2$	6.98	4.01	22.75	90.0	89.54	90.0	−52.796425
PtS_2	6.20	3.58	21.98	90.0	90.1	90.0	−64.708149
$PtSe_2$	6.51	3.76	22.32	90.0	90.2	90.0	−60.631787
$PtTe_2$	4.02	4.02	22.73	90.0	90.0	120.0	−28.919434

Table S7: Trilayer Lattice Parameters obtained from relaxed 2D crystal structures, where a_0,b_0 and c_0 (all values in Angstroms) are the modules of unit cell vectors a, b and c. The total energy (in electronvolts) and α_0, β_0 and γ_0 (all values in degrees) are the angles between unit cell vectors a and b, b and c, a and c, respectively. Also, we have MQ_2 compositions (composition).

Composition	a_0 (Å)	b_0 (Å)	c_0 (Å)	α_0 (°)	β_0 (°)	γ_0 (°)	E_{tot} (eV)
FeS_2	5.29	3.22	29.08	90.0	90.0	90.0	−108.534938
$FeSe_2$	5.54	3.39	29.44	90.0	90.0	90.0	−100.963014
$FeTe_2$	5.91	3.66	30.55	90.0	90.0	90.0	−93.610524
RuS_2	5.54	3.46	34.56	90.0	90.0	90.0	−113.808778
$RuSe_2$	5.76	3.60	29.79	90.0	90.0	90.0	−106.335083
$RuTe_2$	6.10	3.84	30.73	90.0	90.0	90.0	−100.365712
OsS_2	5.52	3.52	29.15	90.0	90.0	90.0	−132.485908
$OsSe_2$	5.74	3.67	29.92	90.0	90.0	90.0	−123.905737
$OsTe_2$	6.09	3.90	30.86	90.0	90.0	90.0	−117.050875
NiS_2	3.37	3.37	26.17	90.0	90.0	120.0	−44.999646
$NiSe_2$	3.57	3.57	26.79	90.0	90.0	120.0	−41.998984
PdS_2	5.49	5.57	23.59	90.0	90.0	90.0	−89.809489
$PdSe_2$	3.77	3.77	26.95	90.0	90.0	120.0	−41.747268
$PdTe_2$	7.01	4.04	27.77	90.0	89.6	90.0	−80.068584
PtS_2	6.21	3.59	26.56	90.0	90.1	90.0	−97.451975
$PtSe_2$	6.52	3.77	27.09	90.0	90.2	90.0	−91.445215
$PtTe_2$	4.04	4.04	27.76	90.0	90.0	120.0	−43.707356

Table S8: Tetralayer Lattice Parameters obtained from relaxed 2D crystal structures, where a_0,b_0 and c_0 (all values in Angstroms) are the modules of unit cell vectors a, b and c. The total energy (in electronvolts) and α_0, β_0 and γ_0 (all values in degrees) are the angles between unit cell vectors a and b, b and c, a and c, respectively. Also, we have MQ_2 compositions (composition).

Composition	a_0 (Å)	b_0 (Å)	c_0 (Å)	α_0 (°)	β_0 (°)	γ_0 (°)	E_{tot} (eV)
FeS_2	5.29	3.22	34.66	90.0	90.0	90.0	−144.860537
$FeSe_2$	5.54	3.39	35.09	90.0	90.0	90.0	−134.829436
$FeTe_2$	5.92	3.67	36.61	90.0	90.0	90.0	−125.078956
RuS_2	5.54	3.46	34.56	90.0	90.0	90.0	−151.937706
$RuSe_2$	5.76	3.61	35.54	90.0	90.0	90.0	−142.005387
$RuTe_2$	6.10	3.85	36.80	90.0	90.0	90.0	−134.117326
OsS_2	5.52	3.52	34.68	90.0	90.0	90.0	−176.850655
$OsSe_2$	5.74	3.67	35.72	90.0	90.0	90.0	−165.446560
$OsTe_2$	6.09	3.90	36.97	90.0	90.0	90.0	−156.362666
NiS_2	3.37	3.37	30.61	90.0	90.0	120.0	−60.145766
$NiSe_2$	3.57	3.57	31.47	90.0	90.0	120.0	−56.201399
PdS_2	5.50	5.57	27.27	90.0	90.0	90.0	−120.089064
$PdSe_2$	3.77	3.77	31.64	90.0	90.0	120.0	−55.939584
$PdTe_2$	7.01	4.05	32.78	90.0	89.6	90.0	−107.422189
PtS_2	6.23	3.59	31.14	90.0	90.0	90.0	−130.205356
$PtSe_2$	6.53	3.78	31.86	90.0	90.2	90.0	−122.279480
$PtTe_2$	4.04	4.04	32.80	90.0	90.0	120.0	−58.508356

Table S9: Pentalayer Lattice Parameters obtained from relaxed 2D crystal structures, where a_0,b_0 and c_0 (all values in Angstroms) are the modules of unit cell vectors a, b and c. The total energy (in electronvolts) and α_0, β_0 and γ_0 (all values in degrees) are the angles between unit cell vectors a and b, b and c, a and c, respectively. Also, we have MQ_2 compositions (composition).

Composition	a_0 (Å)	b_0 (Å)	c_0 (Å)	α_0 (°)	β_0 (°)	γ_0 (°)	E_{tot} (eV)
FeS_2	5.29	3.23	40.24	90.0	90.0	90.0	−181.184206
$FeSe_2$	5.55	3.40	40.74	90.0	90.0	90.0	−168.687263
$FeTe_2$	5.92	3.67	42.67	90.0	90.0	90.0	−156.553731
RuS_2	5.54	3.46	40.06	90.0	90.0	90.0	−190.066815
$RuSe_2$	5.76	3.61	41.29	90.0	90.0	90.0	−177.678324
$RuTe_2$	6.10	3.85	42.86	90.0	90.0	90.0	−167.873389
OsS_2	5.52	3.52	40.22	90.0	90.0	90.0	−221.215546
$OsSe_2$	5.74	3.67	41.52	90.0	90.0	90.0	−206.982800
$OsTe_2$	6.08	3.91	43.09	90.0	90.0	90.0	−195.676237
NiS_2	3.38	3.38	35.06	90.0	90.0	120.0	−75.301958
$NiSe_2$	3.58	3.58	36.16	90.0	90.0	120.0	−70.401152
PdS_2	5.50	5.58	30.95	90.0	90.0	90.0	−150.371312
$PdSe_2$	3.77	3.77	36.33	90.0	90.0	120.0	−70.094703
$PdTe_2$	7.03	4.06	37.79	90.0	89.7	90.0	−134.721770
PtS_2	6.23	3.60	35.73	90.0	90.0	90.0	−162.959952
$PtSe_2$	6.54	3.78	36.64	90.0	90.2	90.0	−153.111207
$PtTe_2$	4.05	4.05	37.83	90.0	90.0	120.0	−73.331059

Table S10: Hexalayer Lattice Parameters obtained from relaxed 2D crystal structures, where a_0,b_0 and c_0 (all values in Angstroms) are the modules of unit cell vectors a, b and c. The total energy (in electronvolts) and α_0, β_0 and γ_0 (all values in degrees) are the angles between unit cell vectors a and b, b and c, a and c, respectively. Also, we have MQ_2 compositions (composition).

Composition	a_0 (Å)	b_0 (Å)	c_0 (Å)	α_0 (°)	β_0 (°)	γ_0 (°)	E_{tot} (eV)
FeS_2	5.29	3.23	45.81	90.0	90.0	90.0	−217.509216
$FeSe_2$	5.55	3.40	46.39	90.0	90.0	90.0	−202.548850
$FeTe_2$	5.92	3.68	48.73	90.0	90.0	90.0	−188.024562
RuS_2	5.54	3.46	60.56	90.0	90.0	90.0	−228.195507
$RuSe_2$	5.76	3.61	47.04	90.0	90.0	90.0	−213.350536
$RuTe_2$	6.10	3.85	48.92	90.0	90.0	90.0	−201.635065
OsS_2	5.52	3.52	45.75	90.0	90.0	90.0	−265.580486
$OsSe_2$	5.74	3.67	47.32	90.0	90.0	90.0	−248.520977
$OsTe_2$	6.08	3.91	65.21	90.0	90.0	90.0	−234.964824
NiS_2	3.38	3.38	39.50	90.0	90.0	120.0	−90.450855
$NiSe_2$	3.58	3.58	40.84	90.0	90.0	120.0	−84.592812
PdS_2	5.51	5.58	34.63	90.0	90.0	90.0	−180.653581
$PdSe_2$	3.77	3.77	41.02	90.0	90.0	120.0	−84.262032
$PdTe_2$	7.03	4.06	42.80	90.0	89.7	90.0	−161.977395
PtS_2	6.23	3.60	40.31	90.0	90.0	90.0	−195.717916
$PtSe_2$	6.54	3.78	41.41	90.0	90.2	90.0	−183.958843
$PtTe_2$	4.06	4.06	42.86	90.0	90.0	120.0	−88.158227

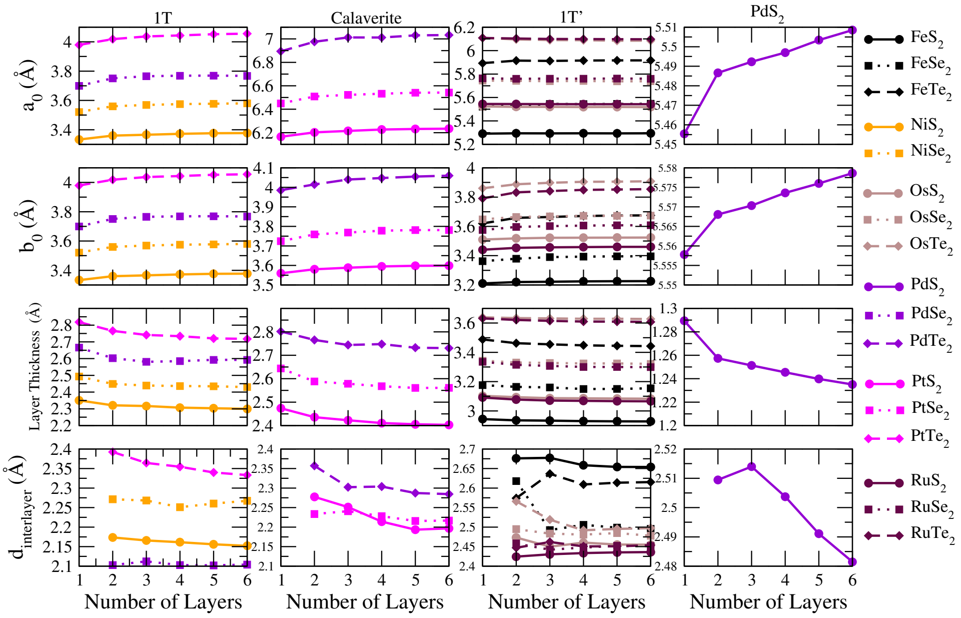

Figure S25: Variation in planar lattice parameters as a function of the number of layers for all investigated materials. The materials were grouped according to their symmetries and separated into four different columns, namely 1T, Calaverite, 1T' and PdS_2. The a_0 and b_0 represents the module of unit cell vectors a and b respectively, layer thickness represents the layer height and interlayer distance ($d_{interlayer}$) represents the distance between layers.

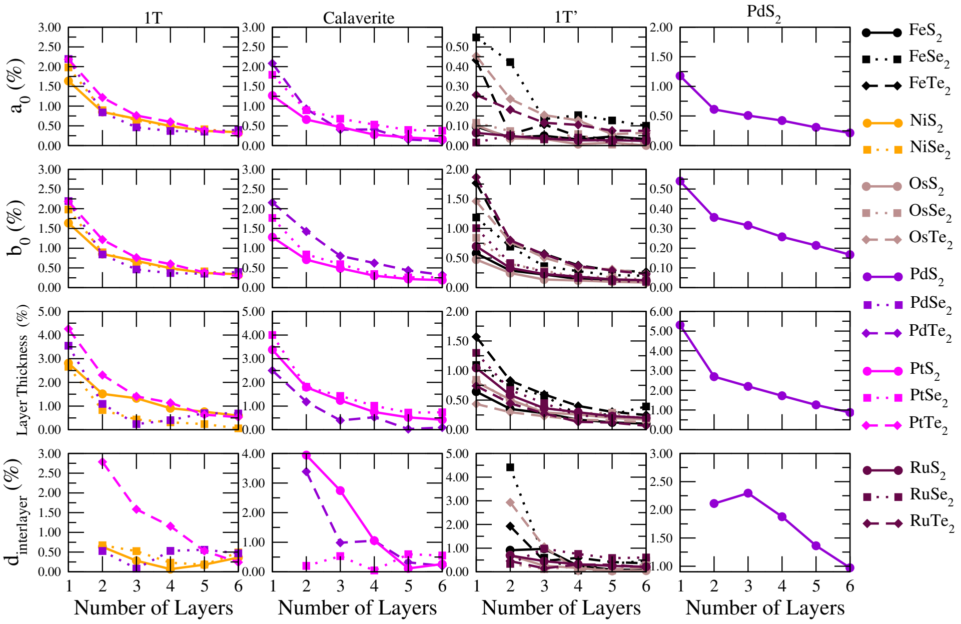

Figure S26: Percentual variation in planar lattice parameters from their bulk values plotted as a function of the number of layers for all investigated materials. The materials were grouped according to their symmetries and separated into four different columns, namely 1T, Calaverite, 1T' and PdS_2. From top to bottom: The a_0 and b_0 represents the module of unit cell vectors a and b respectively, layer thickness represents the layer height and interlayer distance ($d_{interlayer}$) represents the distance between layers.

4.2 Point-Inversion Symmetry Analysis for Few-Layer Materials.

As symmetry, particularly the PIS, has been demonstrated to be a dominating factor in the properties of few-layer 2D TMDs, we present below some specific features evaluated from the optimized structures. For each composition, Table S11 presents the **k**-grid adopted for sampling the reciprocal space integration, the dimensionality (which can be either few-layer or bulk), the Point-Group detected by the symmetry analysis of the VASP code, and the evaluation of the PIS, which may assume values "yes" or "no".

Table S11: Symmetry information for the optimized structures, enclosing the MQ_2 Composition, the Dimensionality (few-layer and bulk), **k**-grid for DFT simulations, the classification of the Point-Group, and Point Inversion Symmetry (PIS), which may assume values "yes" or "no".

Composition	dimensionality	**k**-grid	Point-Group	PIS
FeS_2	few-layer	$8 \times 12 \times 1$	$C_s = C_{1h}$ (for 1 to 6 layers)	No
	bulk	$8 \times 12 \times 4$	C_{2v}	No
$FeSe_2$	few-layer	$7 \times 12 \times 1$	$C_s = C_{1h}$ (for 1 to 6 layers)	No
	bulk	$7 \times 12 \times 4$	C_{2v}	No
$FeTe_2$	few-layer	$7 \times 11 \times 1$	$C_s = C_{1h}$ (for 1 to 6 layers)	No
	bulk	$7 \times 11 \times 3$	C_{2v}	No
RuS_2	few-layer	$7 \times 12 \times 1$	$C_s = C_{1h}$ (for 1 to 6 layers)	No
	bulk	$7 \times 12 \times 4$	C_{2v}	No
$RuSe_2$	few-layer	$7 \times 11 \times 1$	$C_s = C_{1h}$ (for 1 to 6 layers)	No
	bulk	$7 \times 11 \times 3$	C_{2v}	No
$RuTe_2$	few-layer	$7 \times 10 \times 1$	$C_s = C_{1h}$ (for 1 to 6 layers)	No
	bulk	$7 \times 10 \times 3$	C_{2v}	No
OsS_2	few-layer	$7 \times 11 \times 1$	$C_s = C_{1h}$ (for 1 to 6 layers)	No
	bulk	$7 \times 11 \times 4$	C_{2v}	No
$OsSe_2$	few-layer	$7 \times 11 \times 1$	$C_s = C_{1h}$ (for 1 to 6 layers)	No
	bulk	$7 \times 11 \times 3$	C_{2v}	No
$OsTe_2$	few-layer	$7 \times 10 \times 1$	$C_s = C_{1h}$ (for 1 to 6 layers)	No

Continued on next page

Table S11 – continued from previous page

Composition	Structures	**k**-points grid	Point Group	PIS
	bulk	$7 \times 10 \times 3$	C_{2v}	No
NiS_2	few-layer	$14 \times 14 \times 1$	D_{3d} (for 1 to 6 layers)	Yes
	bulk	$14 \times 14 \times 9$	D_{3d}	Yes
$NiSe_2$	few-layer	$13 \times 13 \times 1$	D_{3d} (for 1 to 6 layers)	Yes
	bulk	$13 \times 13 \times 9$	D_{3d}	Yes
	few-layer	$7 \times 7 \times 1$	C_{2h} (for 1; 3 and 5 layers)	Yes
PdS_2	few-layer	$7 \times 7 \times 1$	C_{2v} (for 2; 4 and 6 layers)	No
	bulk	$7 \times 7 \times 5$	D_{2h}	Yes
$PdSe_2$	few-layer	$12 \times 12 \times 1$	D_{3d} (for 1 to 6 layers)	Yes
	bulk	$12 \times 12 \times 9$	D_{3d}	Yes
$PdTe_2$	few-layer	$6 \times 10 \times 1$	C_{2h} (for 1 to 6 layers)	Yes
	bulk	$6 \times 10 \times 8$	C_{2h}	Yes
PtS_2	few-layer	$6 \times 11 \times 1$	C_{2h} (for 1 to 6 layers)	Yes
	bulk	$6 \times 11 \times 9$	C_{2h}	Yes
$PtSe_2$	few-layer	$6 \times 11 \times 1$	C_{2h} (for 1 to 6 layers)	Yes
	bulk	$6 \times 11 \times 8$	C_{2h}	Yes
$PtTe_2$	few-layer	$11 \times 11 \times 1$	D_{3d} (for 1 to 6 layers)	Yes
	bulk	$11 \times 11 \times 8$	D_{3d}	Yes

4.3 Exfoliation, Adsorption and Relaxation Energies

The exfoliation energy (E_{exf}) is given by:

$$E_{exf} = \frac{E^{N}_{\text{tot}} - \sum_{i=1}^{N} E^{i,N}_{\text{tot,frozen}}}{AN_{interfaces}}, \tag{3}$$

Here, N represents the number of layers, and $N_{interfaces}$ denotes the number of interfaces, which is $N-1$ for few-layers and N for bulks. E_{tot}^{N} denotes the total energy divided by the number of formula units in each layer. Such scaling allows for comparing distinct structures, as the number of formula units per layer varies among the 1T, 1T', Calaverite, and PdS_2-type structures. For example, 1T has one formula unit per layer, whereas 1T' has two. $E_{\text{tot,frozen}}^{i,N}$ represents the total energy per formula unit for each individual layer frozen at its interacting geometries. A represents the layer's area.

The adsorption energy for few layers (E_{ads}) is given by:

$$E_{ads} = \frac{E_{\text{tot}}^{\text{N}} - NE_{\text{tot,opt}}^{1}}{AN_{interfaces}}, \tag{4}$$

$E_{\text{tot,opt}}^{1}$ is the total energy per formula unity for the monolayer at its optimized geometry.

The relaxation energy (E_{rel}) calculated as:

$$E_{rel} = \frac{\sum_{i=1}^{N} E_{\text{tot,frozen}}^{i,N}}{AN_{interfaces}} - E_{\text{tot,opt}}^{1} \tag{5}$$

is the average energy increasing induced by interlayer interactions.

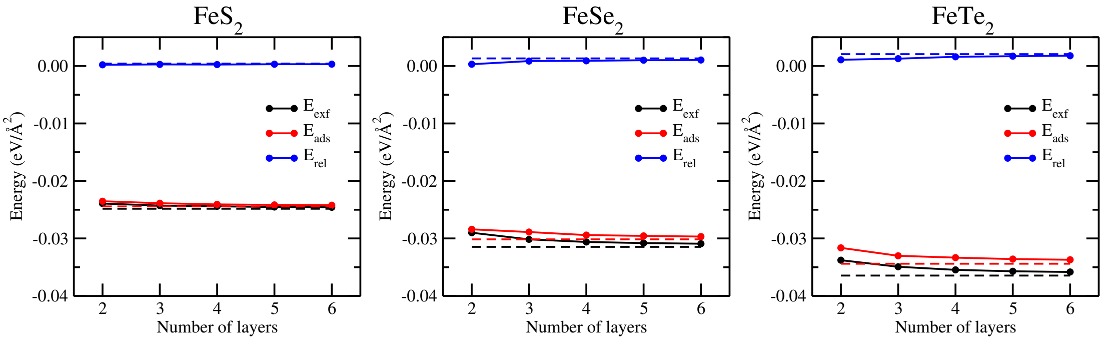

Figure S27: FeQ_2: Exfoliation, adsorption and relaxation energies. Dashed lines indicate the bulk values.

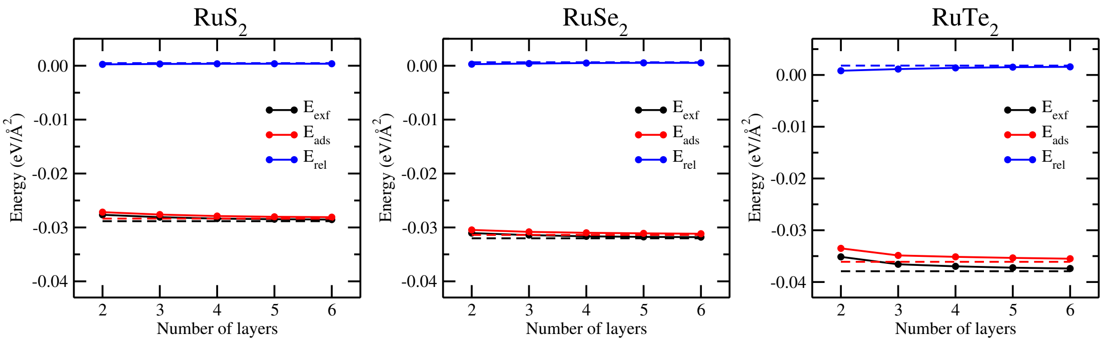

Figure S28: RuQ_2: Exfoliation, adsorption and relaxation energies. Dashed lines indicate the bulk values.

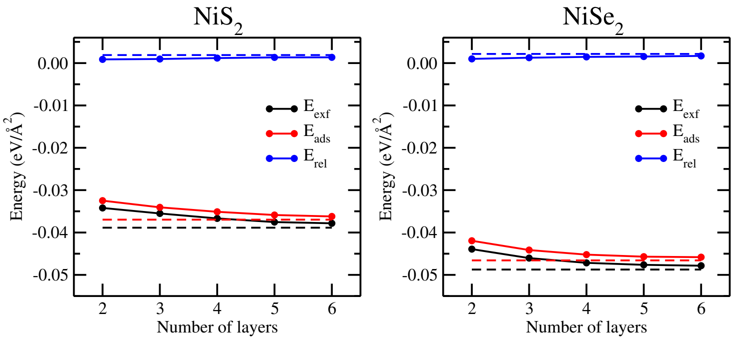

Figure S29: NiQ_2: Exfoliation, adsorption and relaxation energies. Dashed lines indicate the bulk values.

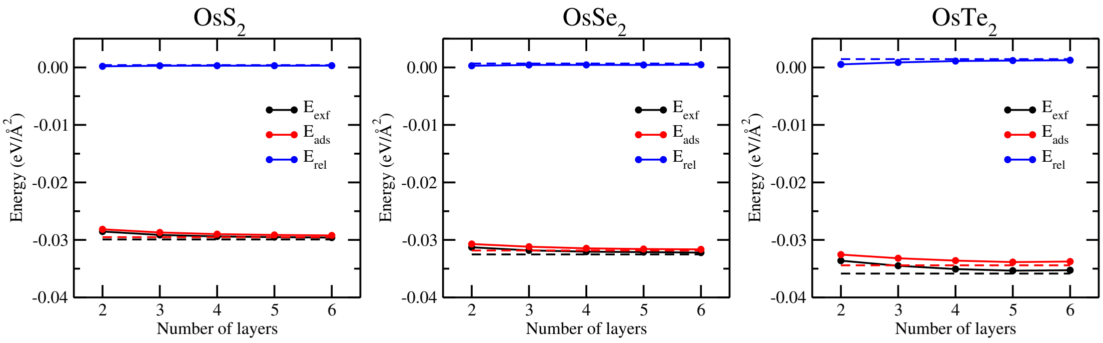

Figure S30: OsQ_2: Exfoliation, adsorption and relaxation energies. Dashed lines indicate the bulk values.

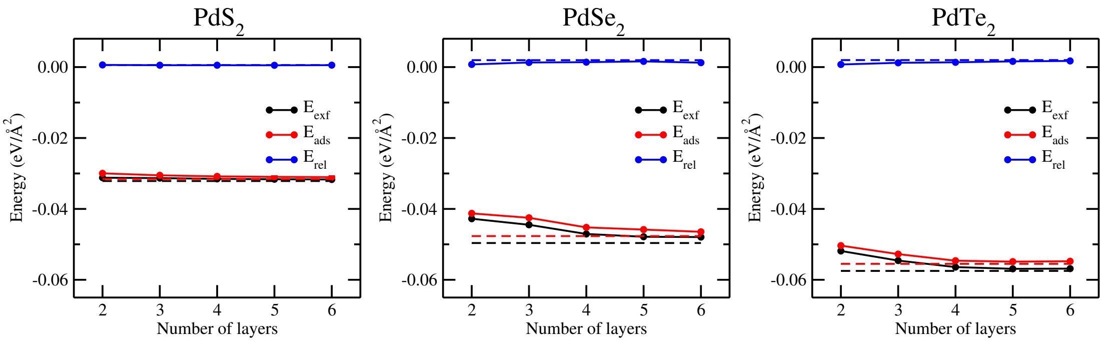

Figure S31: PdQ_2: Exfoliation, adsorption and relaxation energies. Dashed lines indicate the bulk values.

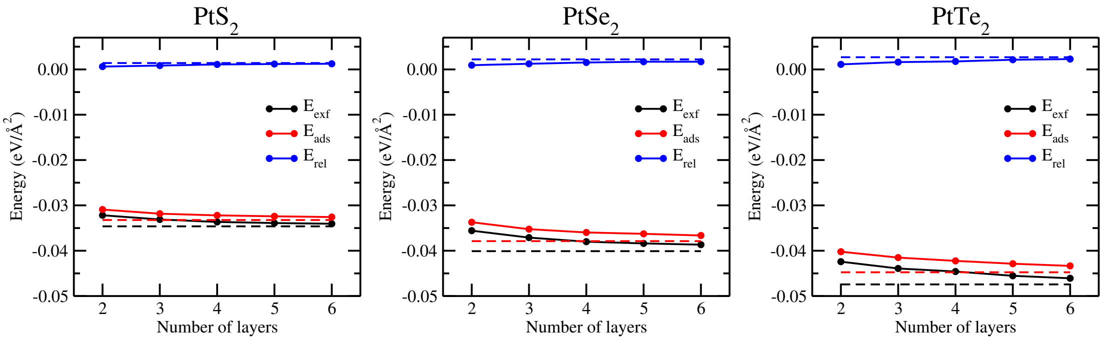

Figure S32: PtQ_2: Exfoliation, adsorption and relaxation energies. Dashed lines indicate the bulk values.

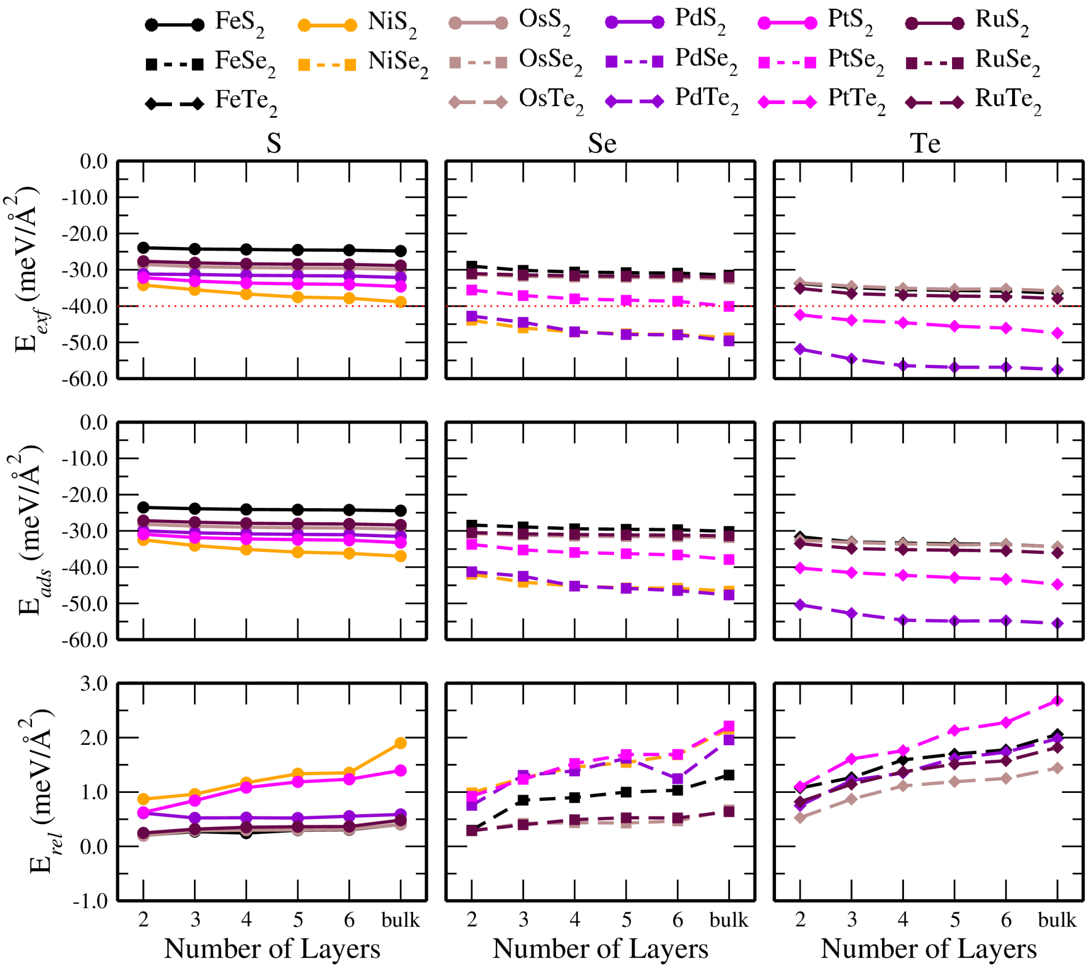

Figure S33: In top, middle and bottom lines, are the exfoliation (E_{exf}), adsorption (E_{ads}) and relaxation (E_{rel}) energies, respectively. For better understanding, every columns represents compounds with different chalcogen.

4.4 Band Structures.

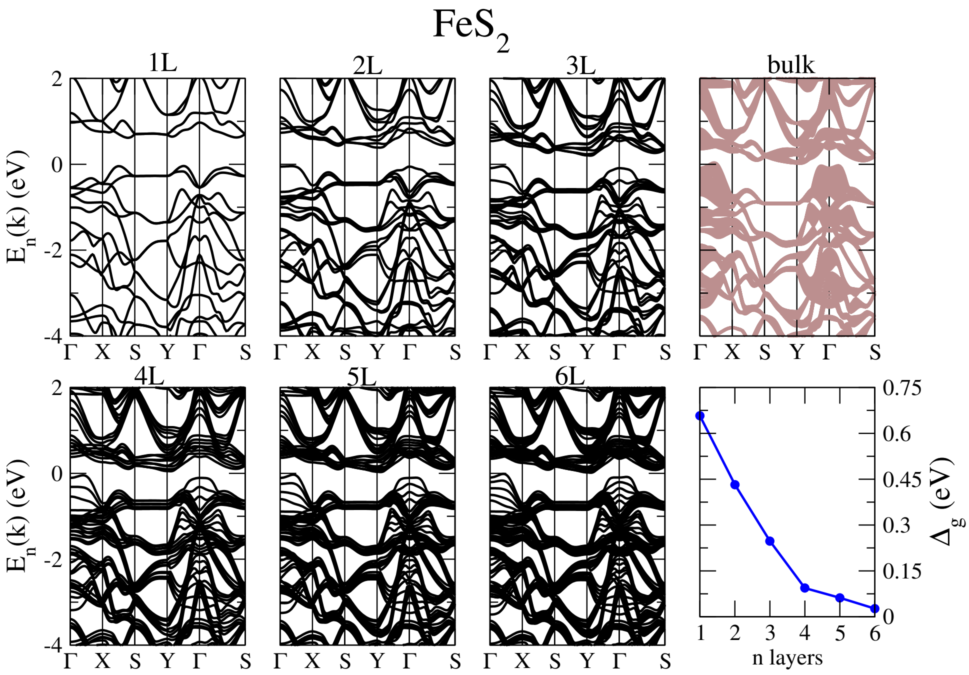

Figure S34: Band structures with dipole correction for few-layer FeS_2 systems, N=1–6 layers; projected bandstructure for the bulk (upper-right panel); and bandgap as a function of the number of layers (bottom-right panel).

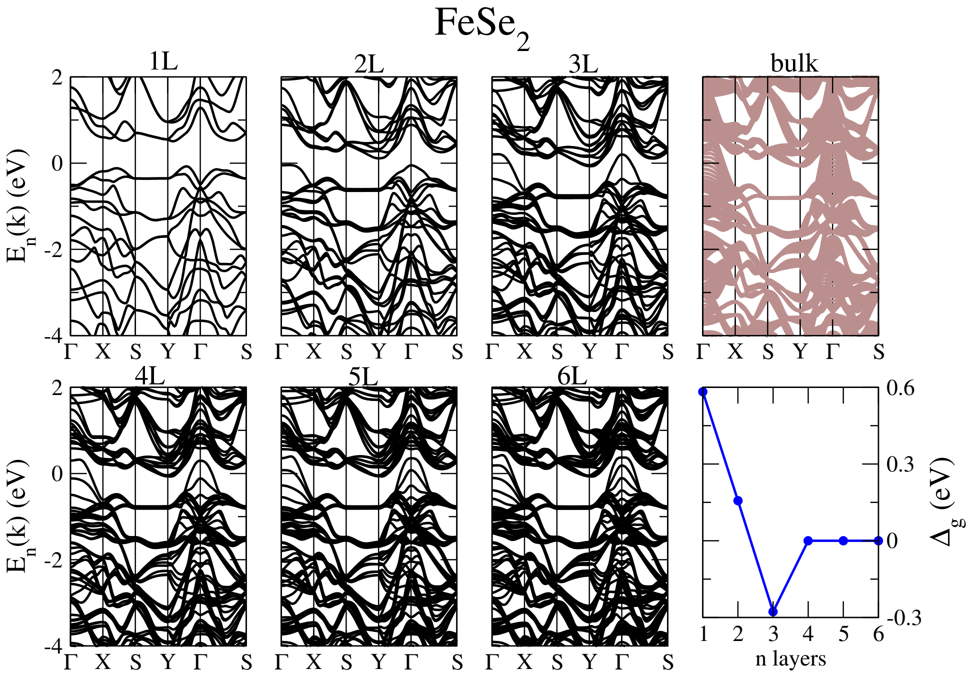

Figure S35: Band structures with dipole correction for few-layer $FeSe_2$ systems, N=1–6 layers; projected bandstructure for the bulk (upper-right panel); and bandgap as a function of the number of layers (bottom-right panel).

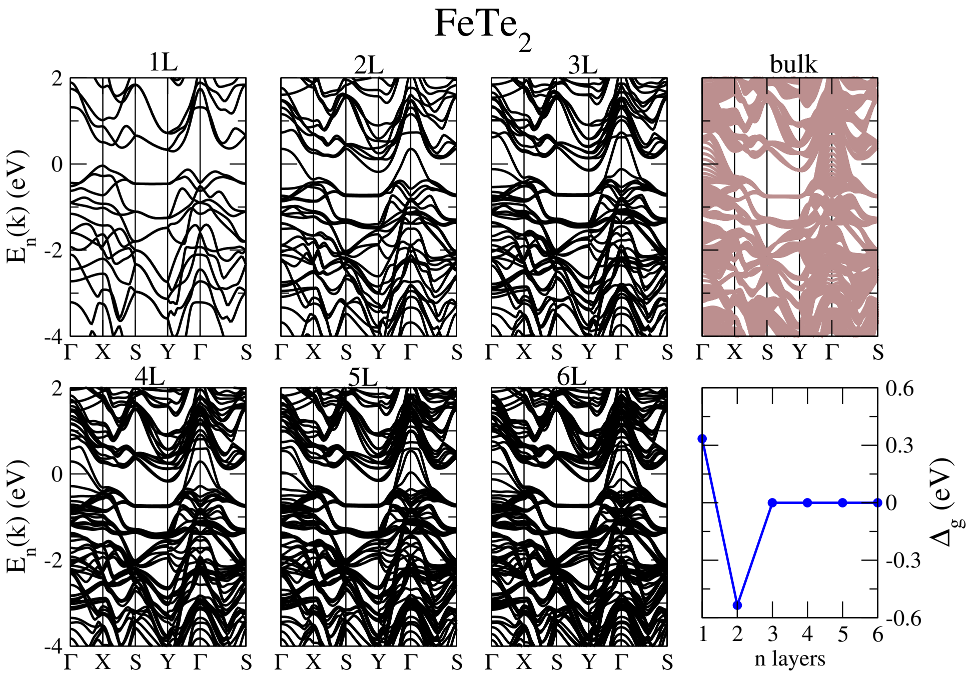

Figure S36: Band structures with dipole correction for few-layer $FeTe_2$ systems, N=1–6 layers; projected bandstructure for the bulk (upper-right panel); and bandgap as a function of the number of layers (bottom-right panel).

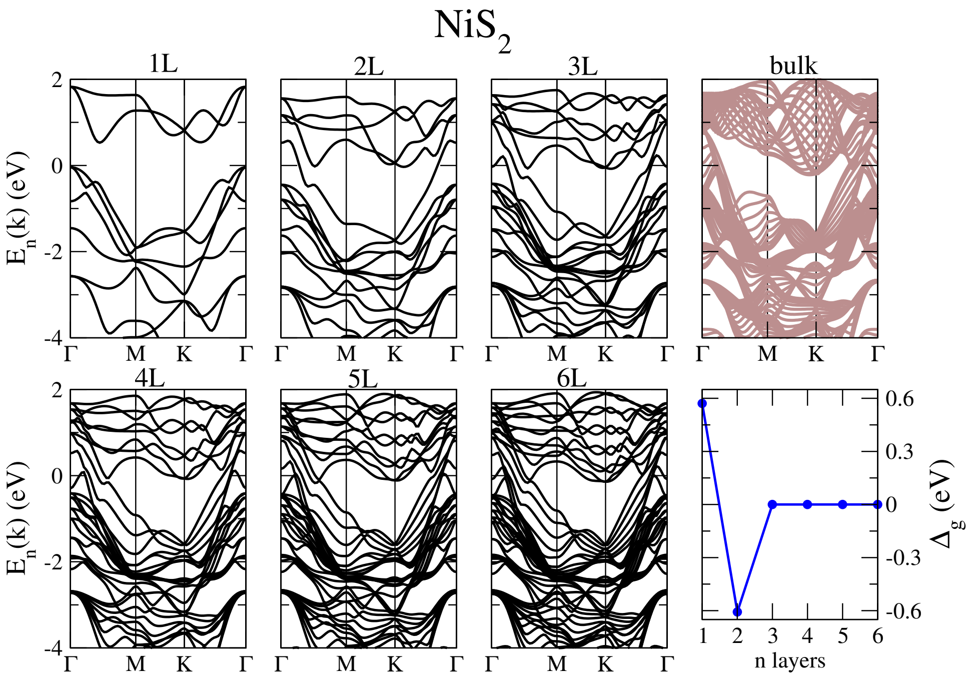

Figure S37: Band structures with dipole correction for few-layer NiS_2 systems, N=1–6 layers; projected bandstructure for the bulk (upper-right panel); and bandgap as a function of the number of layers (bottom-right panel).

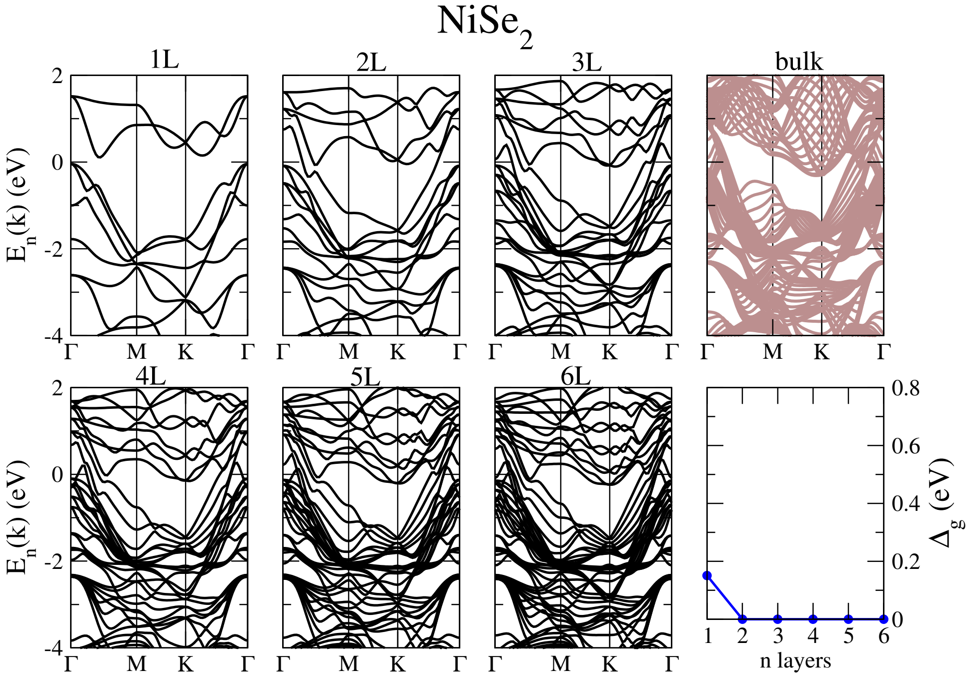

Figure S38: Band structures with dipole correction for few-layer $NiSe_2$ systems, N=1–6 layers; projected bandstructure for the bulk (upper-right panel); and bandgap as a function of the number of layers (bottom-right panel).

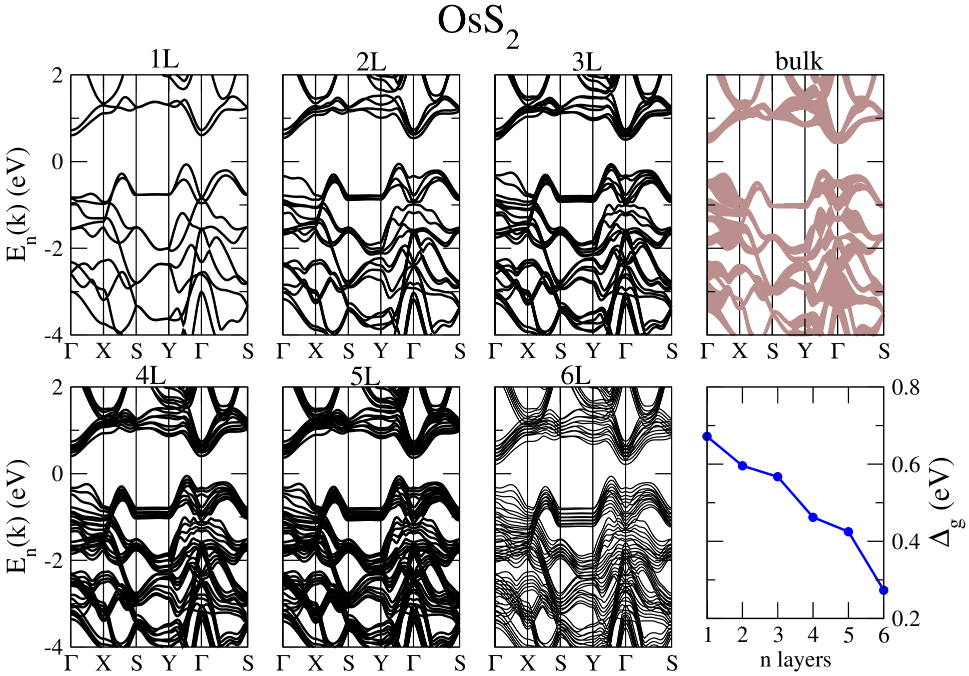

Figure S39: Band structures with dipole correction for few-layer OsS_2 systems, N=1–6 layers; projected bandstructure for the bulk (upper-right panel); and bandgap as a function of the number of layers (bottom-right panel).

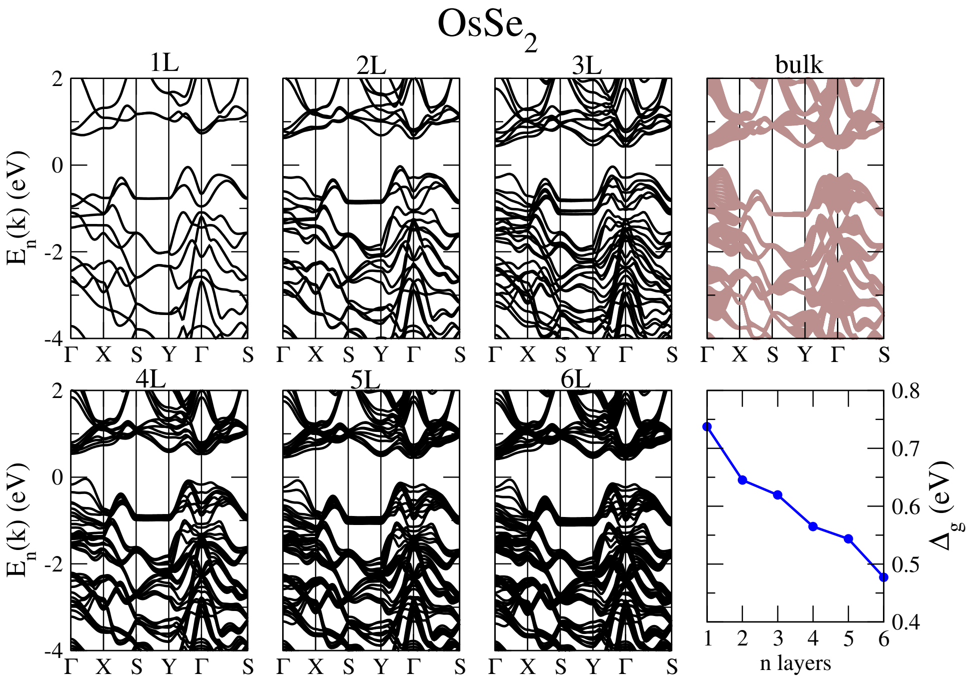

Figure S40: Band structures with dipole correction for few-layer $OsSe_2$ systems, N=1–6 layers; projected bandstructure for the bulk (upper-right panel); and bandgap as a function of the number of layers (bottom-right panel).

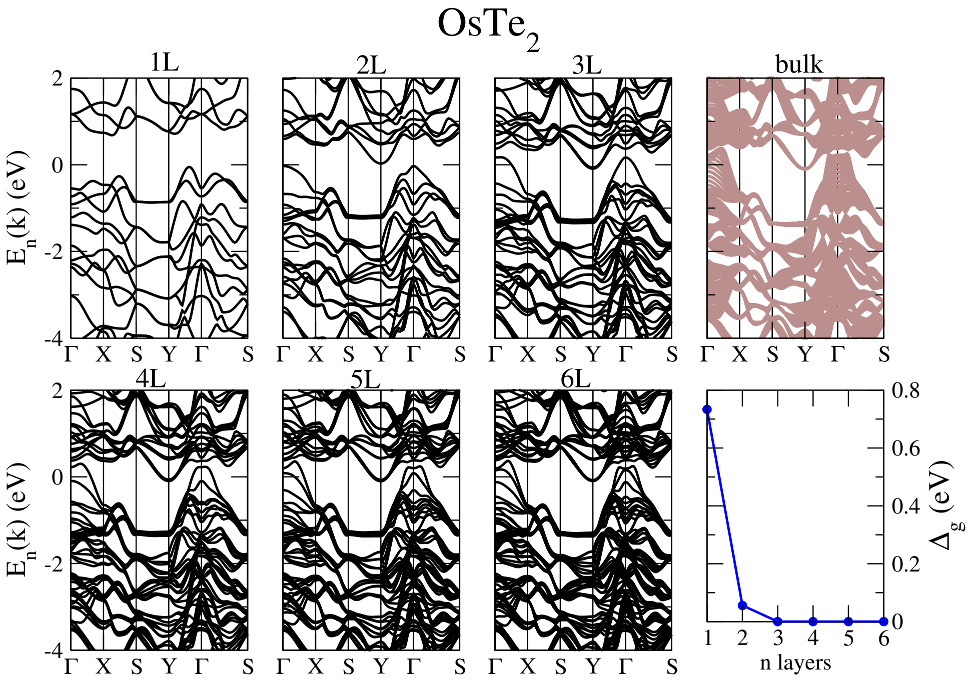

Figure S41: Band structures with dipole correction for few-layer $OsTe_2$ systems, N=1–6 layers; projected bandstructure for the bulk (upper-right panel); and bandgap as a function of the number of layers (bottom-right panel).

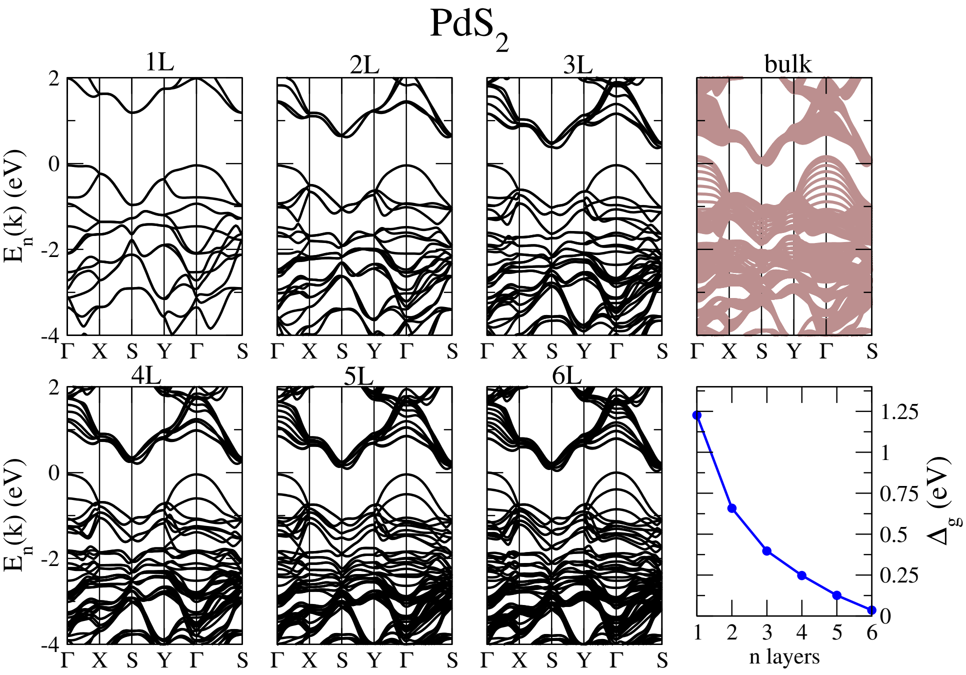

Figure S42: Band structures with dipole correction for few-layer PdS_2 systems, N=1–6 layers; projected bandstructure for the bulk (upper-right panel); and bandgap as a function of the number of layers (bottom-right panel).

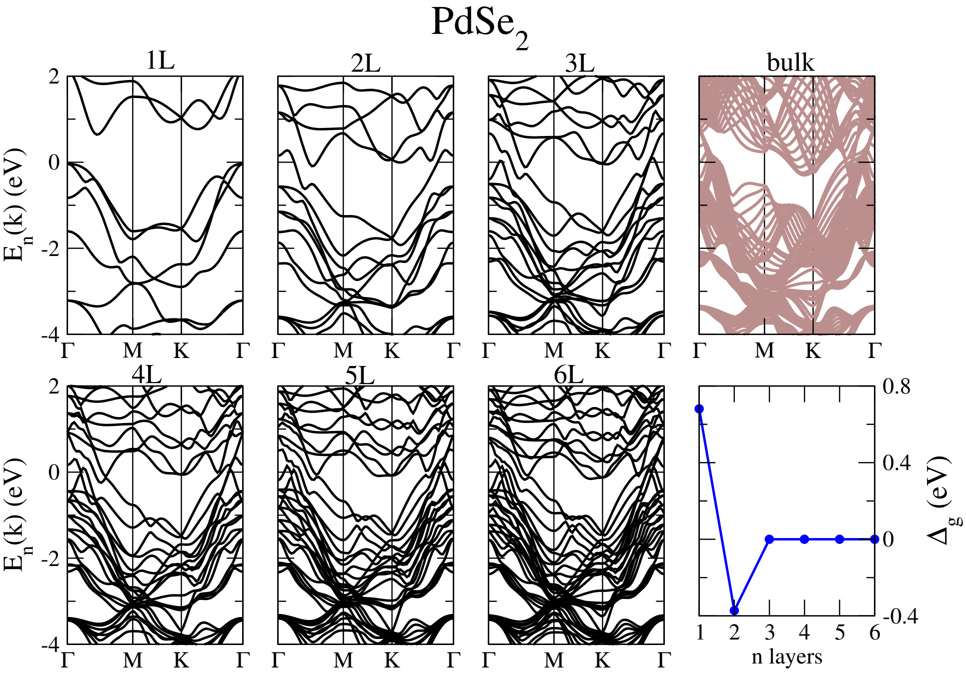

Figure S43: Band structures with dipole correction for few-layer $PdSe_2$ systems, N=1–6 layers; projected bandstructure for the bulk (upper-right panel); and bandgap as a function of the number of layers (bottom-right panel).

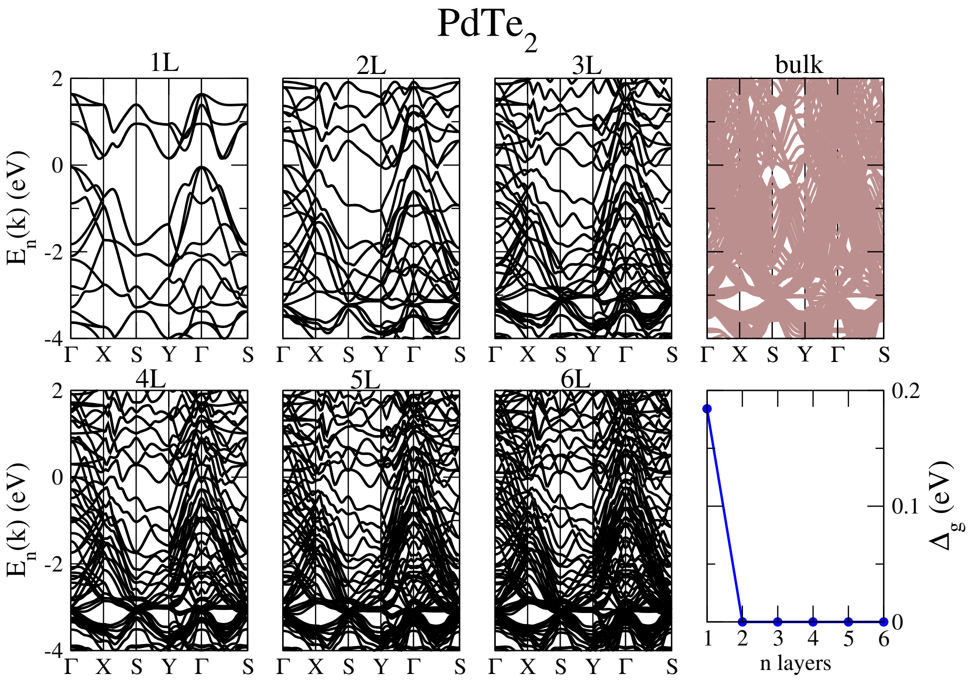

Figure S44: Band structures with dipole correction for few-layer $PdTe_2$ systems, N=1–6 layers; projected bandstructure for the bulk (upper-right panel); and bandgap as a function of the number of layers (bottom-right panel).

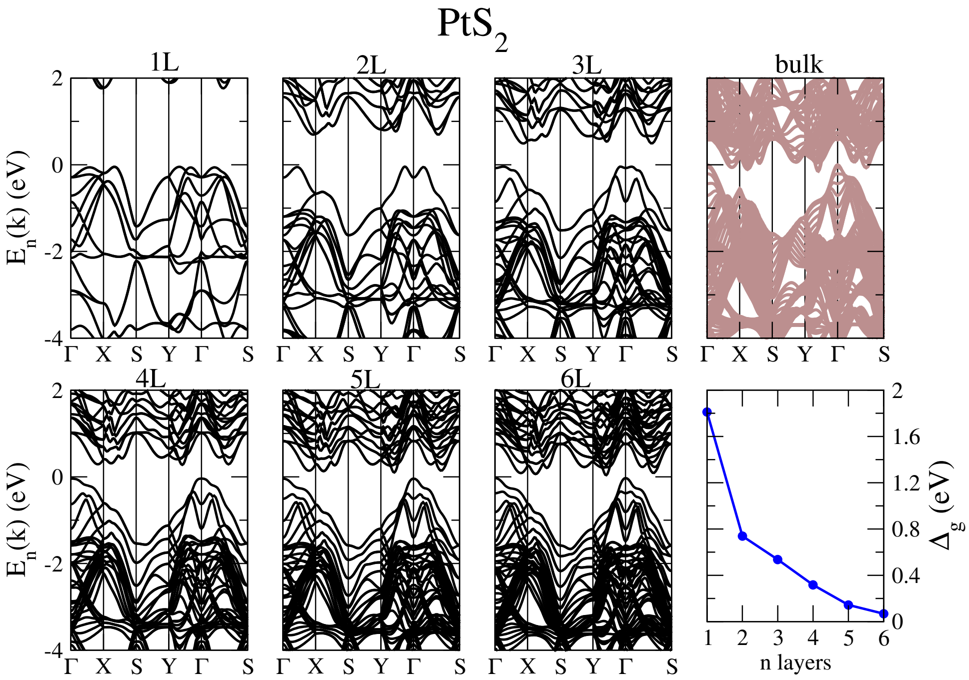

Figure S45: Band structures with dipole correction for few-layer PtS_2 systems, N=1–6 layers; projected bandstructure for the bulk (upper-right panel); and bandgap as a function of the number of layers (bottom-right panel).

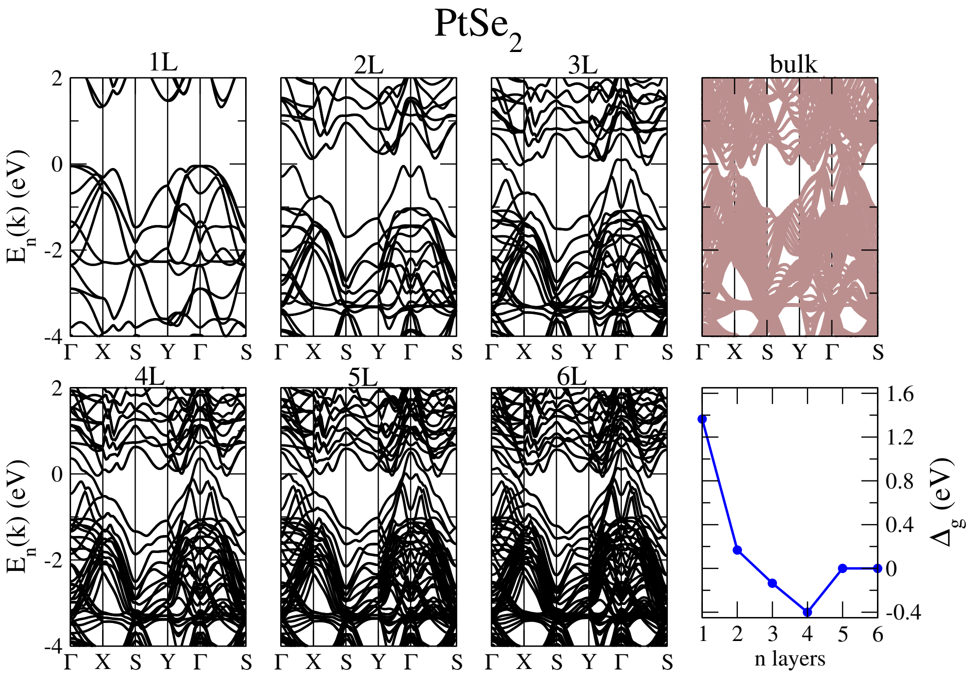

Figure S46: Band structures with dipole correction for few-layer $PtSe_2$ systems, N=1–6 layers; projected bandstructure for the bulk (upper-right panel); and bandgap as a function of the number of layers (bottom-right panel).

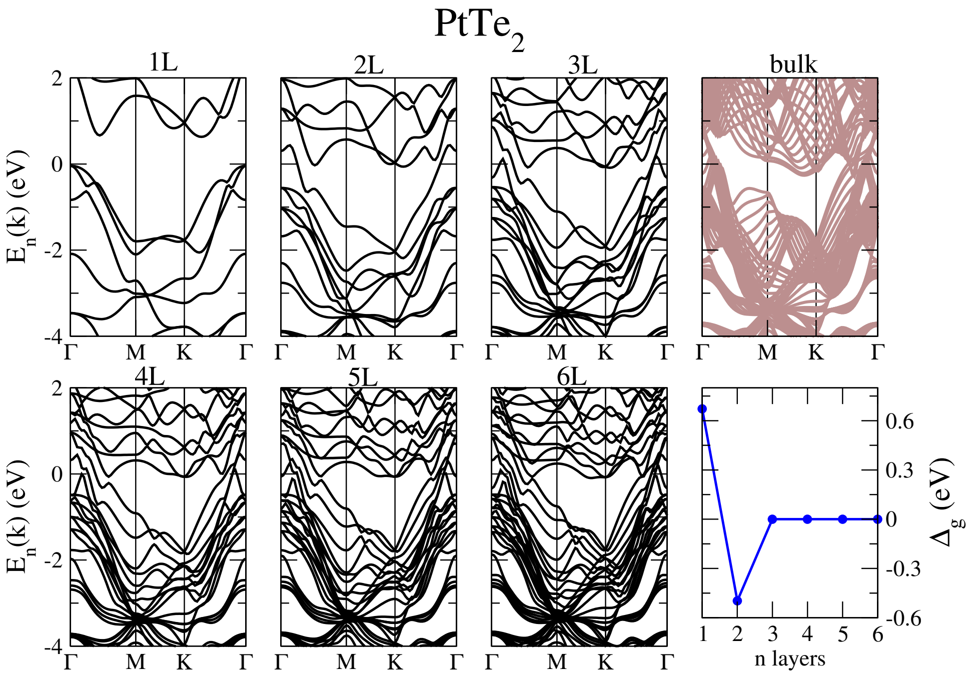

Figure S47: Band structures with dipole correction for few-layer $PtTe_2$ systems, N=1–6 layers; projected bandstructure for the bulk (upper-right panel); and bandgap as a function of the number of layers (bottom-right panel).

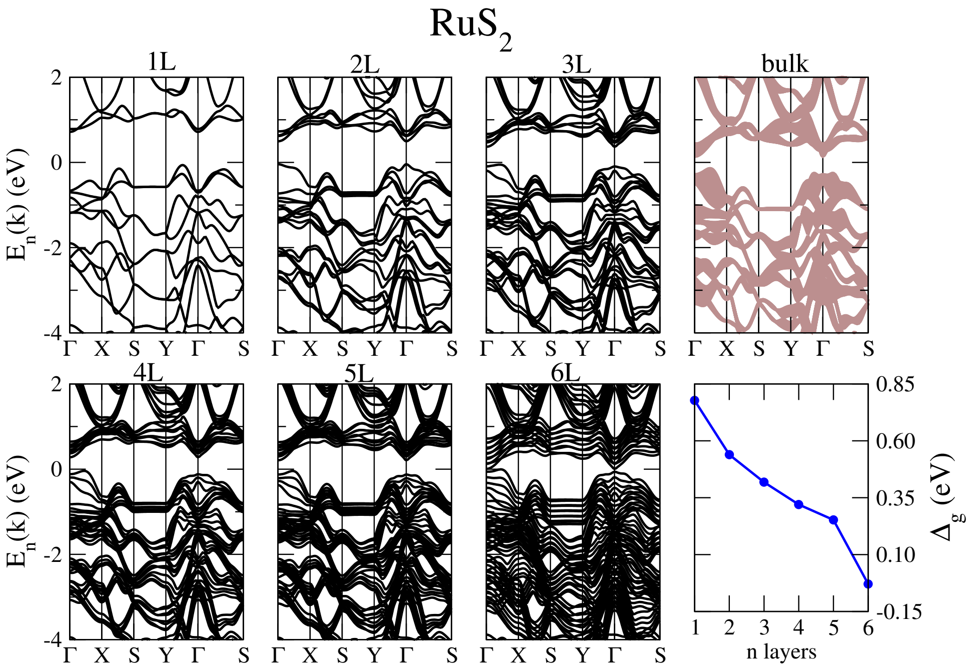

Figure S48: Band structures with dipole correction for few-layer RuS_2 systems, N=1–6 layers; projected bandstructure for the bulk (upper-right panel); and bandgap as a function of the number of layers (bottom-right panel).

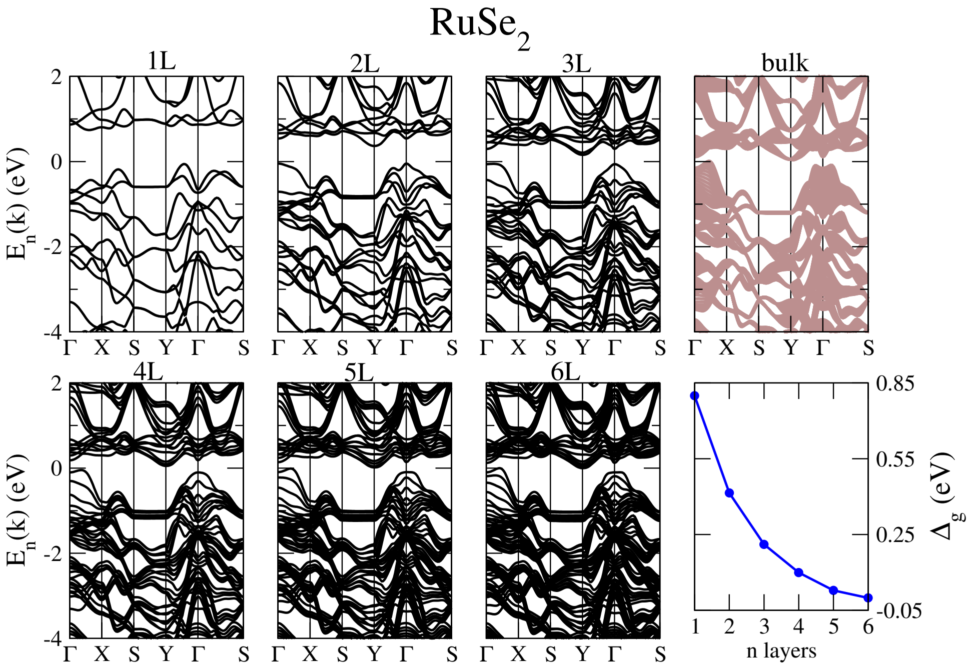

Figure S49: Band structures with dipole correction for few-layer $RuSe_2$ systems, N=1–6 layers; projected bandstructure for the bulk (upper-right panel); and bandgap as a function of the number of layers (bottom-right panel).

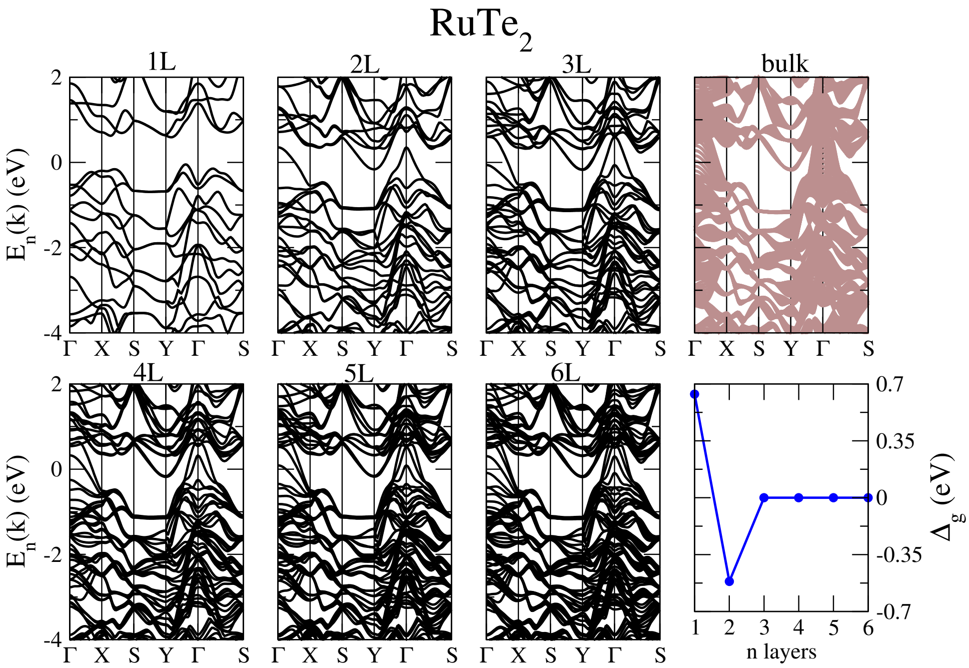

Figure S50: Band structures with dipole correction for few-layer $RuTe_2$ systems, N=1–6 layers; projected bandstructure for the bulk (upper-right panel); and bandgap as a function of the number of layers (bottom-right panel).

4.5 Band Gaps as a Function of the Number of Layers.

Table S12: Band gaps calculated at the $PBE + D3$ level as a function of the number of layers. Band gaps values in eV for all compositions from 1 to 6 layers and bulk.

Composition	1L (eV)	2L (eV)	3L (eV)	4L (eV)	5L (eV)	6L (eV)	bulk (eV)
FeS_2	0.657	0.432	0.248	0.094	0.062	0.026	−0.004
$FeSe_2$	0.583	0.157	−0.277	M	M	M	M
$FeTe_2$	0.334	−0.535	M	M	M	M	M
NiS_2	0.571	−0.607	M	M	M	M	M
$NiSe_2$	0.151	M	M	M	M	M	M
OsS_2	0.672	0.596	0.567	0.462	0.425	0.273	0.668
$OsSe_2$	0.737	0.645	0.619	0.565	0.544	0.477	0.455
$OsTe_2$	0.733	0.056	−0.262	M	M	M	M
PdS_2	1.226	0.658	0.397	0.247	0.126	0.035	−0.214
$PdSe_2$	0.681	−0.372	M	M	M	M	M

Continued on next page

Table S12 – continued from previous page

Composition	1L (eV)	2L (eV)	3L (eV)	4L (eV)	5L (eV)	6L (eV)	bulk (eV)
$PdTe_2$	0.184	M	M	M	M	M	M
PtS_2	1.811	0.738	0.536	0.317	0.144	0.068	0.049
$PtSe_2$	1.365	0.168	−0.136	−0.401	M	M	M
$PtTe_2$	0.672	−0.497	M	M	M	M	M
RuS_2	0.778	0.539	0.418	0.320	0.253	−0.029	0.282
$RuSe_2$	0.799	0.414	0.211	0.099	0.029	−0.001	0.032
$RuTe_2$	0.637	−0.515	M	M	M	M	M

4.6 Band Gap Corrections

Table S13: $\boldsymbol{MQ_2}$, M=Fe, Ni, Os, Pd, Pt and Ru; Q=S, Se, and Te: Band gaps calculated at the PBE+D3 level with $R_k = 40$ ($E_{g}{}^{PBE}_{R_k=40}$) and $R_k = 20$ ($E_{g}{}^{PBE}_{R_k=20}$), and corrections due the spin-orbic coupling (Δ_{SOC}) and hybrid HSE06 exchange-correlation functional (Δ_{HSE06}). The last column presents the corrected band gap value, E_g. All values are presented in eV.

MQ_2	N	$E_{g}{}^{PBE}_{R_k 40}$	$E_{g}{}^{PBE}_{R_k 20}$	$E_{g}{}^{SOC}_{R_k 20}$	$E_{g}{}^{HSE06}_{R_k 20}$	Δ_{SOC}	Δ_{HSE06}	E_g
FeS_2	1L	0.657	0.674	0.696	1.235	−0.022	0.561	1.240
FeS_2	2L	0.432	0.415	0.408	0.725	0.007	0.310	0.735
FeS_2	3L	0.248	0.224	0.217	0.497	0.007	0.273	0.514
FeS_2	4L	0.094	0.118	0.104	0.357	0.014	0.239	0.319
FeS_2	5L	0.062	0.072	0.046	0.275	0.026	0.203	0.239
FeS_2	6L	0.026	0.005	0.000	0.221	0.005	0.216	0.237
FeS_2	bulk	−0.004	−0.005	−0.013	0.245	0.008	0.250	0.238
$FeSe_2$	1L	0.583	0.556	0.580	0.963	−0.024	0.407	1.014
$FeSe_2$	2L	0.157	0.160	0.167	0.374	−0.007	0.214	0.378
$FeSe_2$	3L	−0.277	−0.274	−0.243	−0.311	−0.031	−0.037	−0.283**

Continued on next page

Table S13 – continued from previous page

MQ_2	N	$E_{g_{R_k40}}^{PBE}$	$E_{g_{R_k20}}^{PBE}$	$E_{g_{R_k20}}^{SOC}$	$E_{g_{R_k20}}^{HSE06}$	Δ_{SOC}	Δ_{HSE06}	E_g
$FeTe_2$	1L	0.334	0.334	0.231	0.553	0.103	0.219	0.450
$FeTe_2$	2L	−0.535	−0.533	−0.416	−0.793	−0.117	−0.260	−0.678**
NiS_2	1L	0.571	0.547	0.528	1.221	0.019	0.674	1.226
NiS_2	2L	−0.607	−0.623	−0.614	−0.384	−0.009	0.239	−0.359**
$NiSe_2$	1L	0.151	0.131	−0.013	0.534	0.144	0.403	0.410
OsS_2	1L	0.672	0.663	0.565	1.632	0.098	0.969	1.543
OsS_2	2L	0.596	0.560	0.466	1.437	0.094	0.877	1.379
OsS_2	3L	0.567	0.527	0.408	1.323	0.119	0.796	1.244
OsS_2	4L	0.462	0.473	0.349	1.237	0.124	0.764	1.102
OsS_2	5L	0.425	0.391	0.311	1.155	0.080	0.764	1.109
OsS_2	6L	0.273	0.311	0.227	1.068	0.084	0.757	0.946
OsS_2	bulk	0.668	0.642	0.494	1.298	0.148	0.656	1.176
$OsSe_2$	1L	0.737	0.731	0.612	1.638	0.119	0.907	1.525
$OsSe_2$	2L	0.645	0.639	0.454	1.367	0.185	0.728	1.188
$OsSe_2$	3L	0.619	0.617	0.429	1.163	0.188	0.546	0.977
$OsSe_2$	4L	0.565	0.576	0.383	1.012	0.193	0.436	0.808
$OsSe_2$	5L	0.544	0.538	0.330	0.960	0.208	0.422	0.758
$OsSe_2$	6L	0.477	0.488	0.316	0.902	0.172	0.414	0.719
$OsSe_2$	bulk	0.455	0.451	0.302	0.898	0.149	0.447	0.753
$OsTe_2$	1L	0.733	0.738	0.761	1.577	0.028	0.839	1.544
$OsTe_2$	2L	0.056	0.052	0.143	0.334	−0.091	0.282	0.429
$OsTe_2$	3L	−0.262	−0.267	−0.124	−0.128	−0.143	0.139	0.020 *
PdS_2	1L	1.226	1.195	1.200	2.207	−0.005	1.012	2.243
PdS_2	2L	0.658	0.629	0.649	1.602	−0.020	0.973	1.651
PdS_2	3L	0.397	0.370	0.390	1.314	−0.020	0.944	1.361
PdS_2	4L	0.247	0.220	0.242	1.146	−0.022	0.926	1.195

Continued on next page

Table S13 – continued from previous page

MQ_2	N	$E_{g_{R_k}40}^{PBE}$	$E_{g_{R_k}20}^{PBE}$	$E_{g_{R_k}20}^{SOC}$	$E_{g_{R_k}20}^{HSE06}$	Δ_{SOC}	Δ_{HSE06}	E_g
PdS_2	5L	0.126	0.099	0.122	1.012	−0.023	0.913	1.062
PdS_2	6L	0.035	0.010	0.033	0.910	−0.023	0.900	0.958
PdS_2	bulk	−0.214	−0.237	−0.228	0.633	−0.009	0.870	0.665
$PdSe_2$	1L	0.681	0.659	0.511	1.084	0.148	0.425	0.958
$PdSe_2$	2L	−0.372	−0.393	−0.387	−0.248	−0.006	0.145	−0.221**
$PdTe_2$	1L	0.184	0.171	−0.149	0.442	0.149	0.320	0.135
PtS_2	1L	1.811	1.780	1.774	2.617	0.006	0.837	2.642
PtS_2	2L	0.738	0.712	0.778	1.329	−0.066	0.617	1.421
PtS_2	3L	0.536	0.509	0.571	1.023	−0.062	0.514	1.112
PtS_2	4L	0.317	0.290	0.352	0.758	−0.062	0.468	0.847
PtS_2	5L	0.144	0.116	0.182	0.562	−0.066	0.446	0.656
PtS_2	6L	0.068	0.039	0.105	0.478	−0.066	0.439	0.573
PtS_2	bulk	0.049	−0.183	−0.134	0.232	−0.049	0.415	0.513
$PtSe_2$	1L	1.365	1.345	1.181	1.900	0.164	0.555	1.756
$PtSe_2$	2L	0.168	0.156	0.183	0.541	−0.027	0.385	0.580
$PtSe_2$	3L	−0.136	−0.145	−0.118	0.118	−0.027	0.263	0.154 *
$PtSe_2$	4L	−0.401	−0.415	−0.389	−0.225	−0.026	0.190	−0.185**
$PtTe_2$	1L	0.672	0.659	0.280	1.058	0.379	0.399	0.692
$PtTe_2$	2L	−0.497	−0.510	−0.567	−0.380	0.057	0.130	−0.424**
RuS_2	1L	0.778	0.767	0.741	1.657	0.026	0.890	1.642
RuS_2	2L	0.539	0.536	0.498	1.028	0.038	0.492	0.993
RuS_2	3L	0.418	0.418	0.393	0.884	0.025	0.466	0.859
RuS_2	4L	0.320	0.320	0.298	0.777	0.022	0.457	0.755
RuS_2	5L	0.253	0.257	0.234	0.722	0.023	0.465	0.695
RuS_2	6L	−0.029	0.161*	0.121	0.640	0.040*	0.479*	0.410 *

Continued on next page

Table S13 – continued from previous page

MQ_2	N	$E_{g R_k 40}^{PBE}$	$E_{g R_k 20}^{PBE}$	$E_{g R_k 20}^{SOC}$	$E_{g R_k 20}^{HSE06}$	Δ_{SOC}	Δ_{HSE06}	E_g
RuS_2	bulk	0.282	0.279	0.267	0.732	0.012	0.453	0.723
$RuSe_2$	1L	0.799	0.794	0.776	1.684	0.018	0.890	1.671
$RuSe_2$	2L	0.414	0.411	0.429	0.798	−0.018	0.387	0.819
$RuSe_2$	3L	0.211	0.209	0.228	0.524	−0.019	0.315	0.545
$RuSe_2$	4L	0.099	0.101	0.119	0.401	−0.018	0.300	0.417
$RuSe_2$	5L	0.029	0.035	0.098	0.322	−0.063	0.287*	0.379
$RuSe_2$	6L	−0.001	−0.071*	−0.001	0.211	−0.070*	0.282*	0.351 *
$RuSe_2$	bulk	0.032	0.030	0.046	0.349	−0.016	0.319	0.367
$RuTe_2$	1L	0.637	0.632	0.621	1.265	0.011	0.633	1.259
$RuTe_2$	2L	−0.515	−0.521	−0.351	−0.492	−0.170	0.029	−0.316**

4.7 Electrostatic Potential Energy as a Function of the Number of Layers.

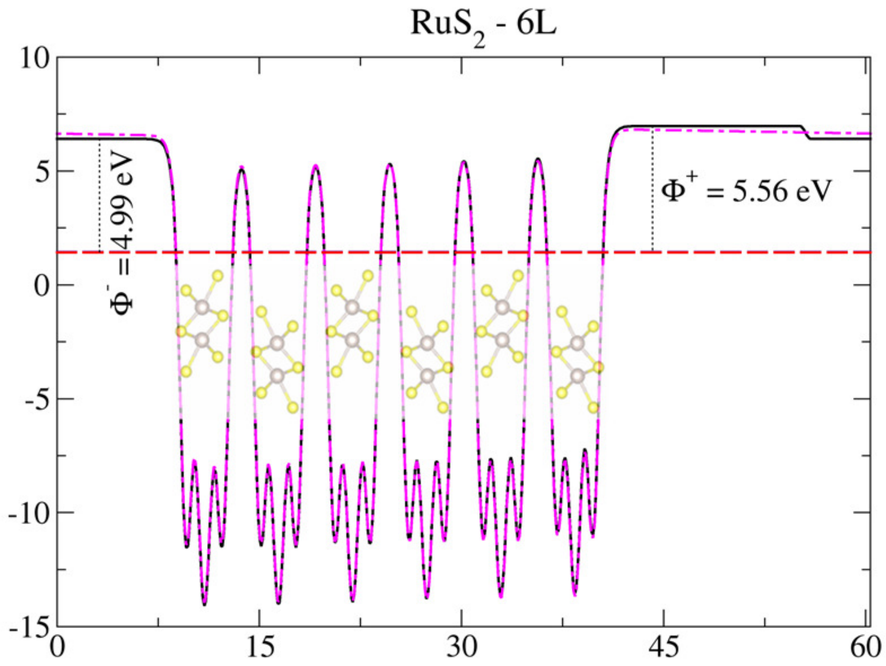

Figure S51: Electrostatic Potential energy for 6 layers RuS_2. The black solid line is the system with dipole correction and, the magenta dotted and dashed line, the system without dipole correction. The dashed blue and red lines are respectively the Conduction Band Maximum (CBM) and the Valence Band Maximum (VBM), where, the Work Functions (Φ) are given by $\Phi^+ = E_{vac}{}^{highest} - VBM$ and $\Phi^- = E_{vac}{}^{lowest} - VBM$.

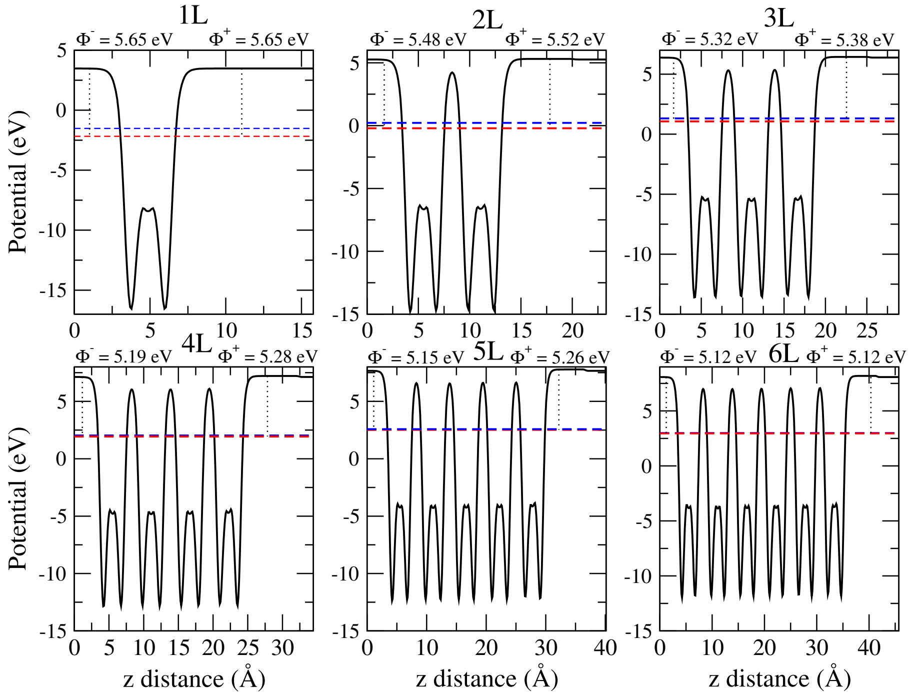

Figure S52: Electrostatic Potential energy as a function of the number of layers for FeS_2. The dashed blue and red lines are respectively the Conduction Band Maximum (CBM) and the Valence Band Maximum (VBM), where, the Work Functions (Φ) are given by $\Phi^+ = {E_{vac}}^{highest} - VBM$ and $\Phi^- = {E_{vac}}^{lowest} - VBM$.

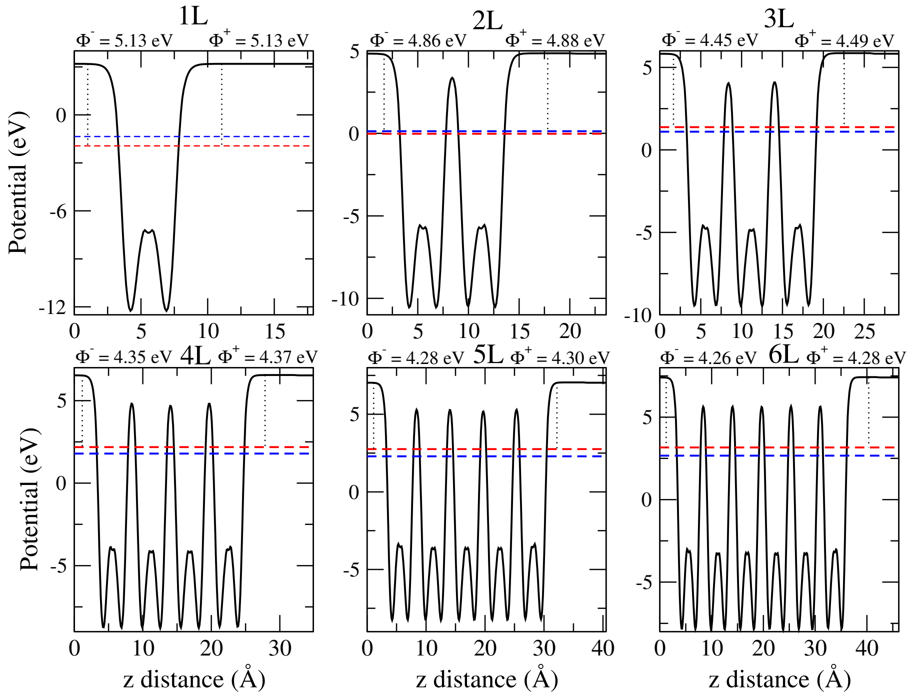

Figure S53: Electrostatic Potential energy as a function of the number of layers for $FeSe_2$. The dashed blue and red lines are respectively the Conduction Band Maximum (CBM) and the Valence Band Maximum (VBM), where, the Work Functions (Φ) are given by $\Phi^+ = {E_{vac}}^{highest} - VBM$ and $\Phi^- = {E_{vac}}^{lowest} - VBM$.

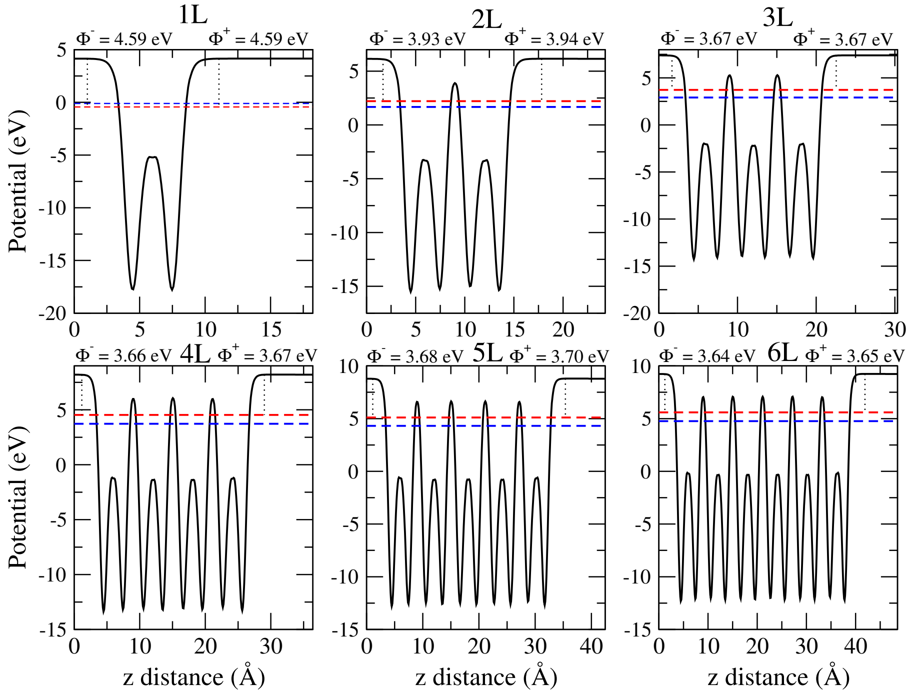

Figure S54: Electrostatic Potential energy as a function of the number of layers for $FeTe_2$. The dashed blue and red lines are respectively the Conduction Band Maximum (CBM) and the Valence Band Maximum (VBM), where, the Work Functions (Φ) are given by $\Phi^+ = E_{vac}{}^{highest} - VBM$ and $\Phi^- = E_{vac}{}^{lowest} - VBM$.

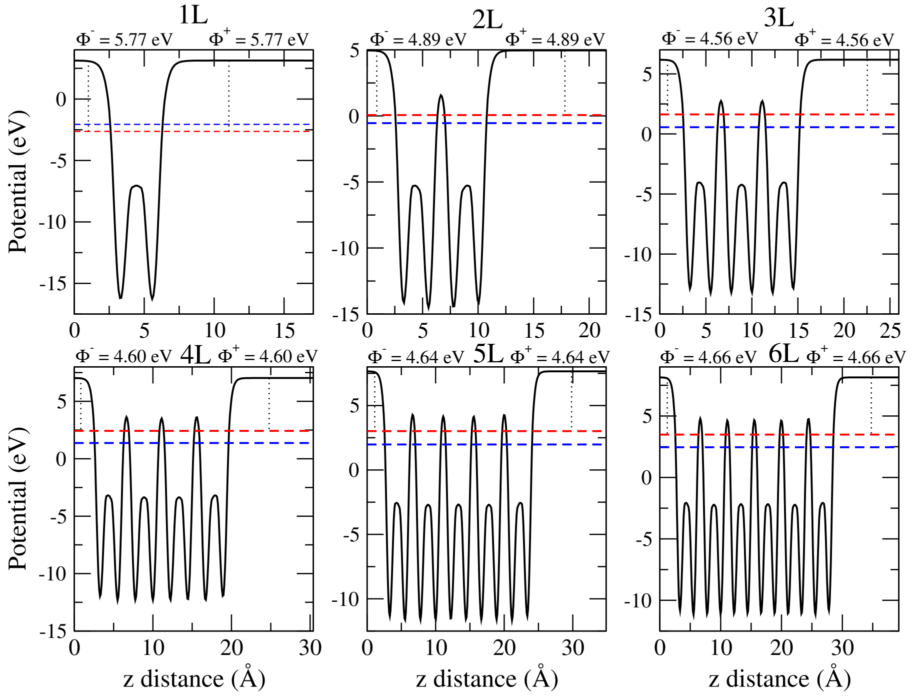

Figure S55: Electrostatic Potential energy as a function of the number of layers for NiS_2. The dashed blue and red lines are respectively the Conduction Band Maximum (CBM) and the Valence Band Maximum (VBM), where, the Work Functions (Φ) are given by $\Phi^+ = E_{vac}{}^{highest} - VBM$ and $\Phi^- = E_{vac}{}^{lowest} - VBM$.

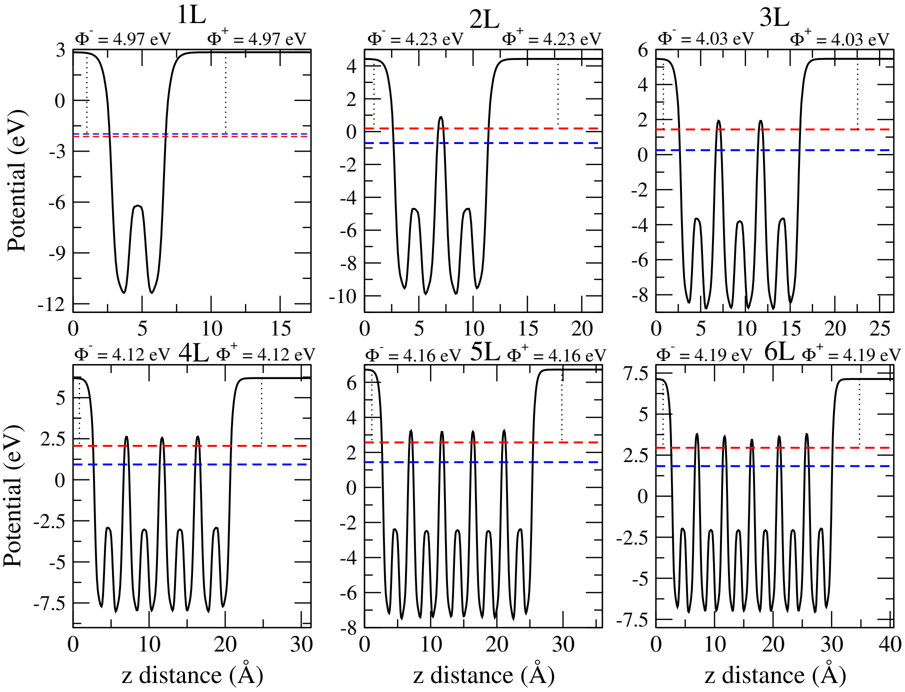

Figure S56: Electrostatic Potential energy as a function of the number of layers for $NiSe_2$. The dashed blue and red lines are respectively the Conduction Band Maximum (CBM) and the Valence Band Maximum (VBM), where, the Work Functions (Φ) are given by $\Phi^+ = E_{vac}{}^{highest} - VBM$ and $\Phi^- = E_{vac}{}^{lowest} - VBM$.

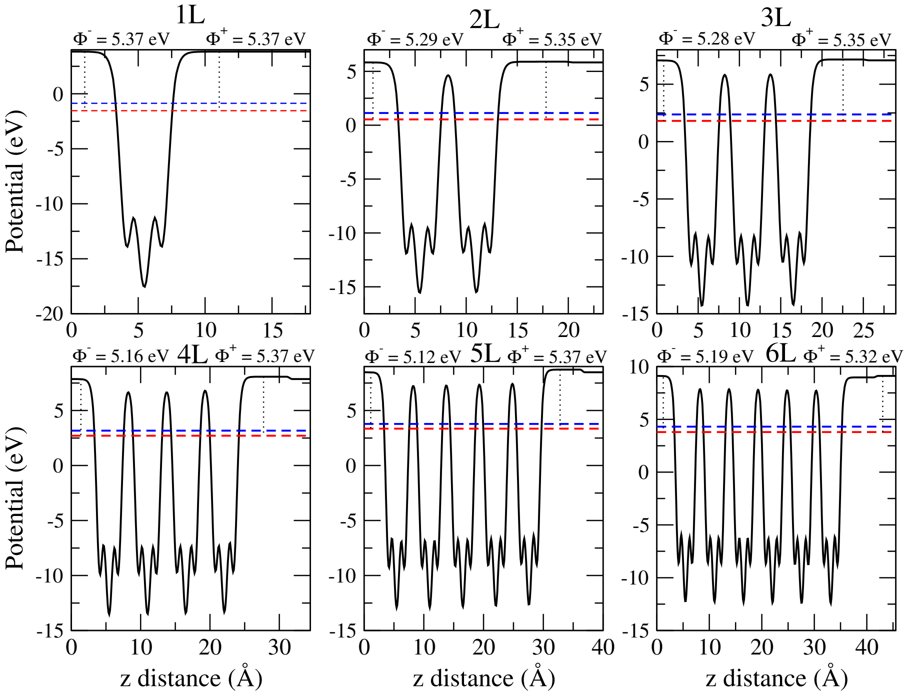

Figure S57: Electrostatic Potential energy as a function of the number of layers for OsS_2. The dashed blue and red lines are respectively the Conduction Band Maximum (CBM) and the Valence Band Maximum (VBM), where, the Work Functions (Φ) are given by $\Phi^+ = E_{vac}{}^{highest} - VBM$ and $\Phi^- = E_{vac}{}^{lowest} - VBM$.

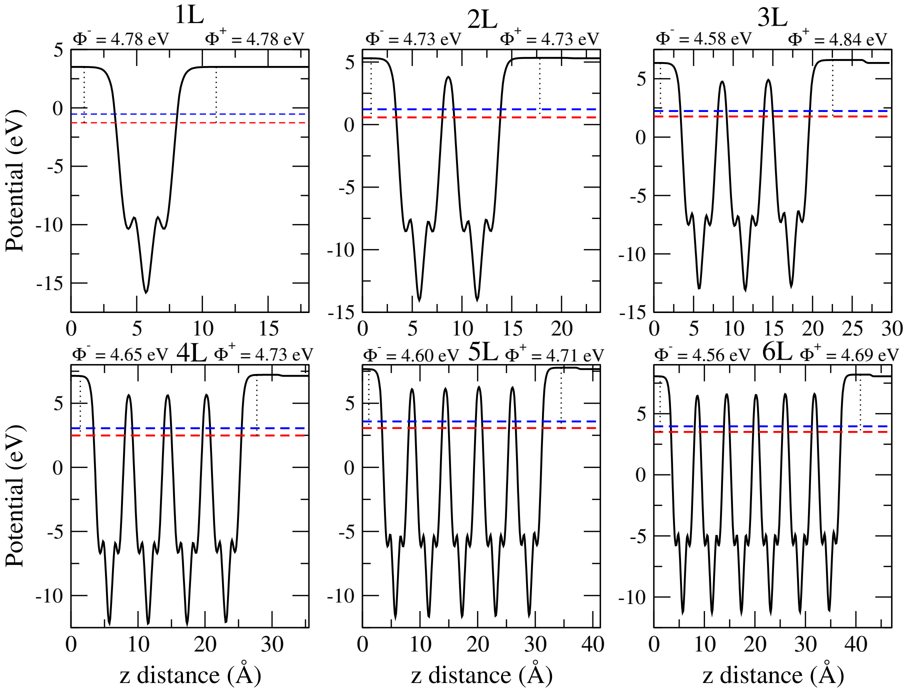

Figure S58: Electrostatic Potential energy as a function of the number of layers for $OsSe_2$. The dashed blue and red lines are respectively the Conduction Band Maximum (CBM) and the Valence Band Maximum (VBM), where, the Work Functions (Φ) are given by $\Phi^+ = E_{vac}{}^{highest} - VBM$ and $\Phi^- = E_{vac}{}^{lowest} - VBM$.

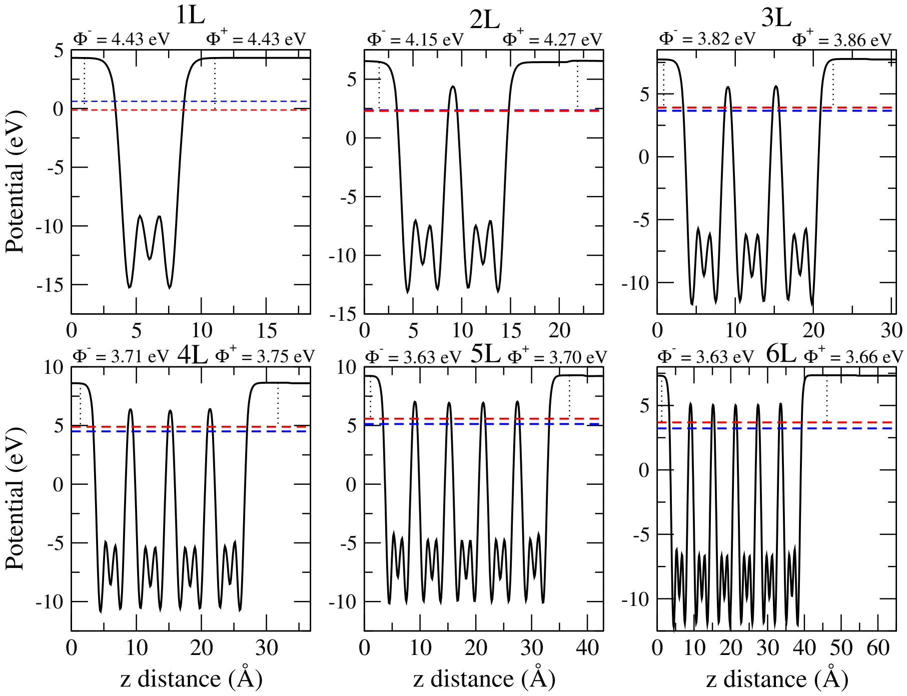

Figure S59: Electrostatic Potential energy as a function of the number of layers for $OsTe_2$. The dashed blue and red lines are respectively the Conduction Band Maximum (CBM) and the Valence Band Maximum (VBM), where, the Work Functions (Φ) are given by $\Phi^+ = E_{vac}{}^{highest} - VBM$ and $\Phi^- = E_{vac}{}^{lowest} - VBM$.

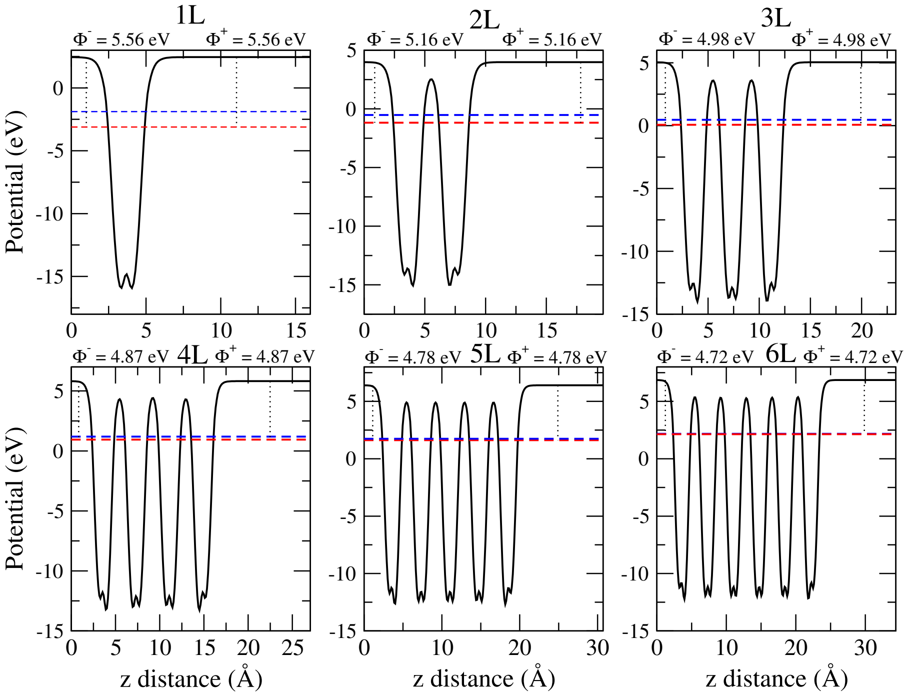

Figure S60: Electrostatic Potential energy as a function of the number of layers for PdS_2. The dashed blue and red lines are respectively the Conduction Band Maximum (CBM) and the Valence Band Maximum (VBM), where, the Work Functions (Φ) are given by $\Phi^+ = E_{vac}{}^{highest} - VBM$ and $\Phi^- = E_{vac}{}^{lowest} - VBM$.

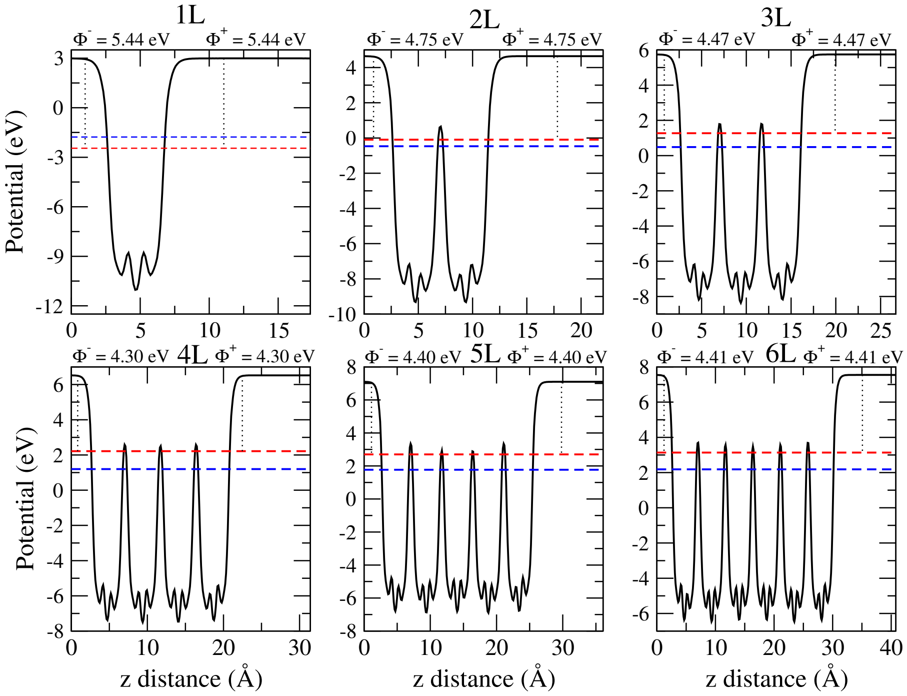

Figure S61: Electrostatic Potential energy as a function of the number of layers for $PdSe_2$. The dashed blue and red lines are respectively the Conduction Band Maximum (CBM) and the Valence Band Maximum (VBM), where, the Work Functions (Φ) are given by $\Phi^+ = E_{vac}{}^{highest} - VBM$ and $\Phi^- = E_{vac}{}^{lowest} - VBM$.

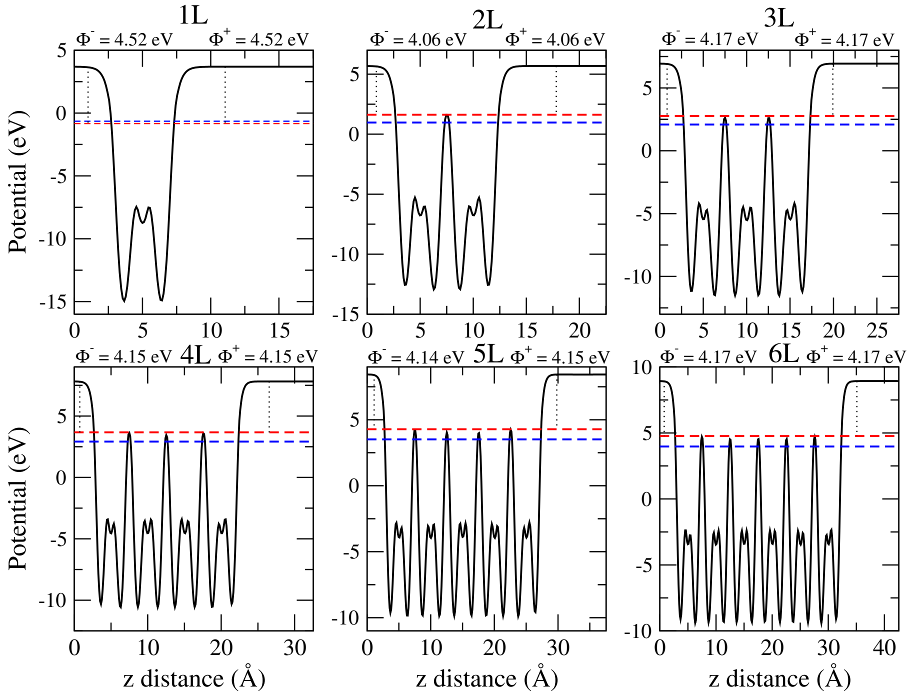

Figure S62: Electrostatic Potential energy as a function of the number of layers for $PdTe_2$. The dashed blue and red lines are respectively the Conduction Band Maximum (CBM) and the Valence Band Maximum (VBM), where, the Work Functions (Φ) are given by $\Phi^+ = {E_{vac}}^{highest} - VBM$ and $\Phi^- = {E_{vac}}^{lowest} - VBM$.

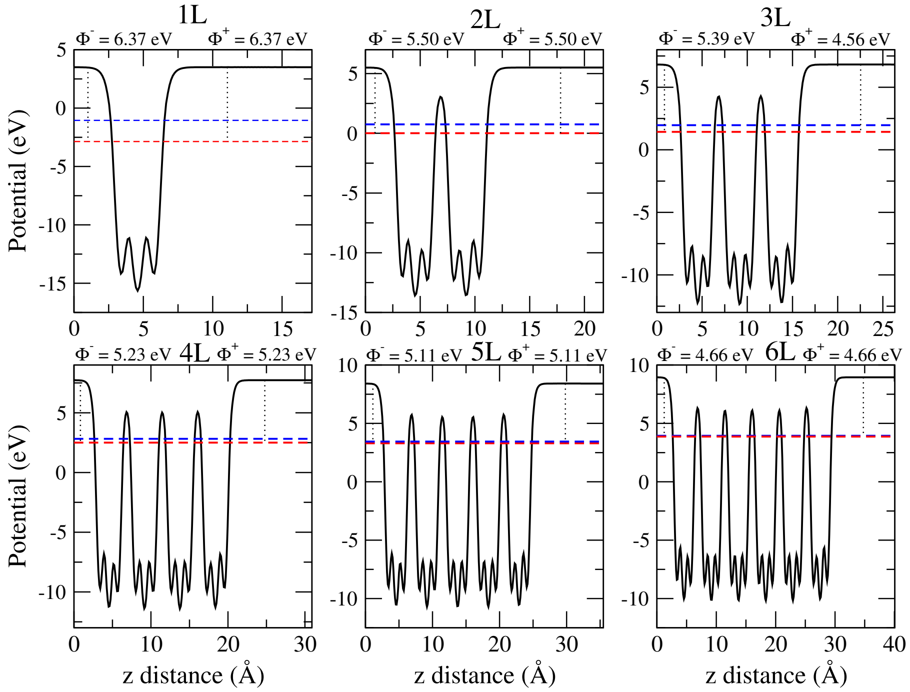

Figure S63: Electrostatic Potential energy as a function of the number of layers for PtS_2. The dashed blue and red lines are respectively the Conduction Band Maximum (CBM) and the Valence Band Maximum (VBM), where, the Work Functions (Φ) are given by $\Phi^+ = {E_{vac}}^{highest} - VBM$ and $\Phi^- = {E_{vac}}^{lowest} - VBM$.

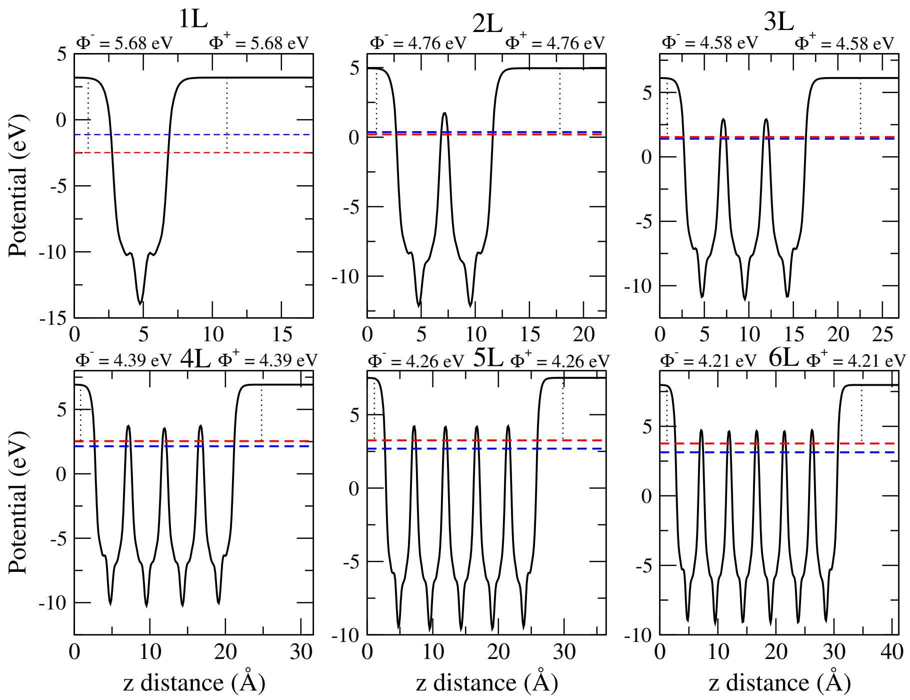

Figure S64: Electrostatic Potential energy as a function of the number of layers for $PtSe_2$. The dashed blue and red lines are respectively the Conduction Band Maximum (CBM) and the Valence Band Maximum (VBM), where, the Work Functions (Φ) are given by $\Phi^+ = {E_{vac}}^{highest} - VBM$ and $\Phi^- = {E_{vac}}^{lowest} - VBM$.

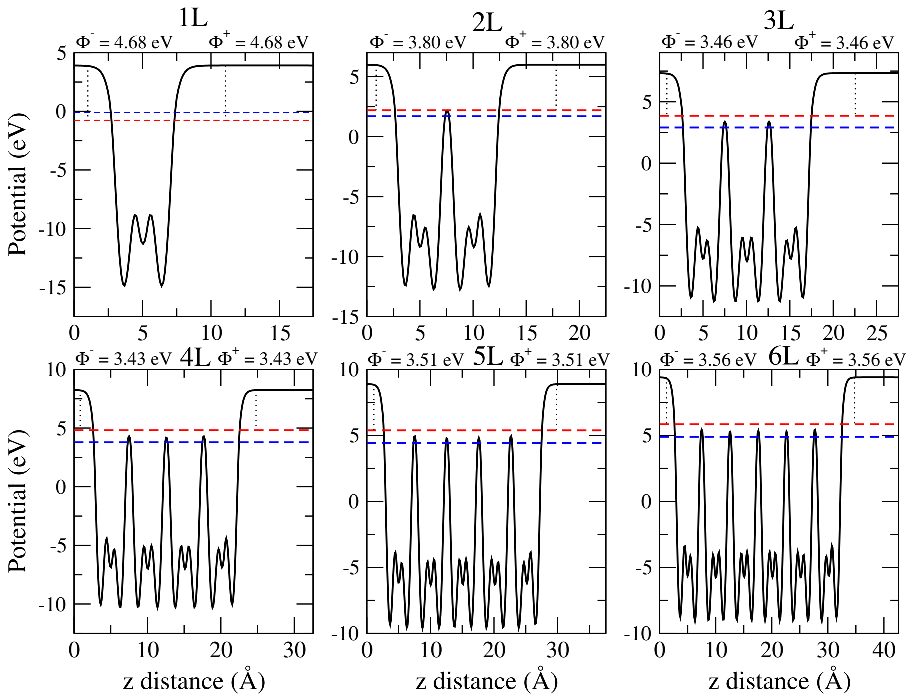

Figure S65: Electrostatic Potential energy as a function of the number of layers for $PtTe_2$. The dashed blue and red lines are respectively the Conduction Band Maximum (CBM) and the Valence Band Maximum (VBM), where, the Work Functions (Φ) are given by $\Phi^+ = {E_{vac}}^{highest} - VBM$ and $\Phi^- = {E_{vac}}^{lowest} - VBM$.

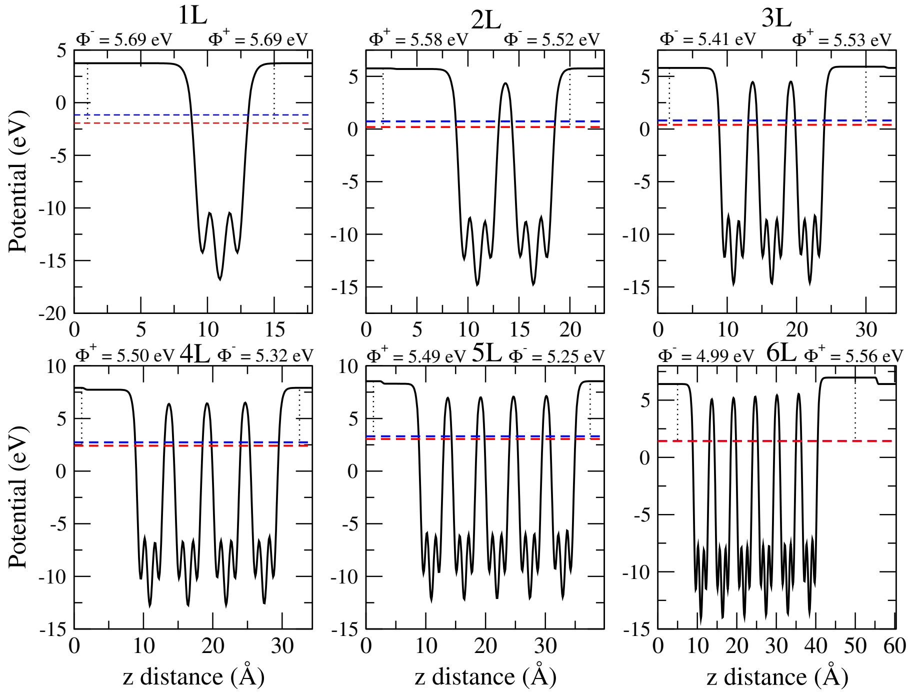

Figure S66: Electrostatic Potential energy as a function of the number of layers for RuS_2. The dashed blue and red lines are respectively the Conduction Band Maximum (CBM) and the Valence Band Maximum (VBM), where, the Work Functions (Φ) are given by $\Phi^+ = {E_{vac}}^{highest} - VBM$ and $\Phi^- = {E_{vac}}^{lowest} - VBM$.

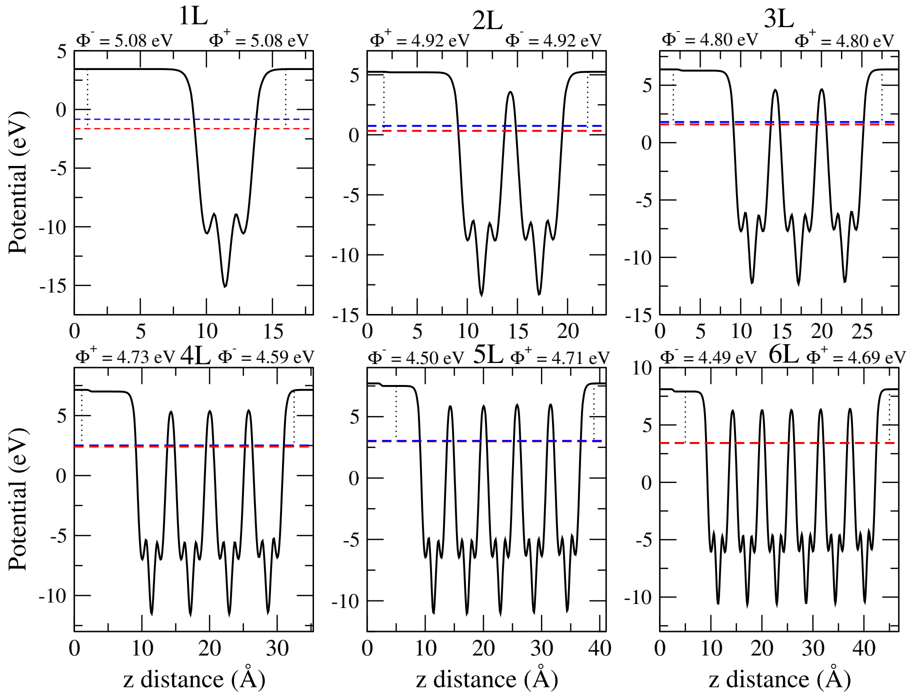

Figure S67: Electrostatic Potential energy as a function of the number of layers for $RuSe_2$. The dashed blue and red lines are respectively the Conduction Band Maximum (CBM) and the Valence Band Maximum (VBM), where, the Work Functions (Φ) are given by $\Phi^+ = {E_{vac}}^{highest} - VBM$ and $\Phi^- = {E_{vac}}^{lowest} - VBM$.

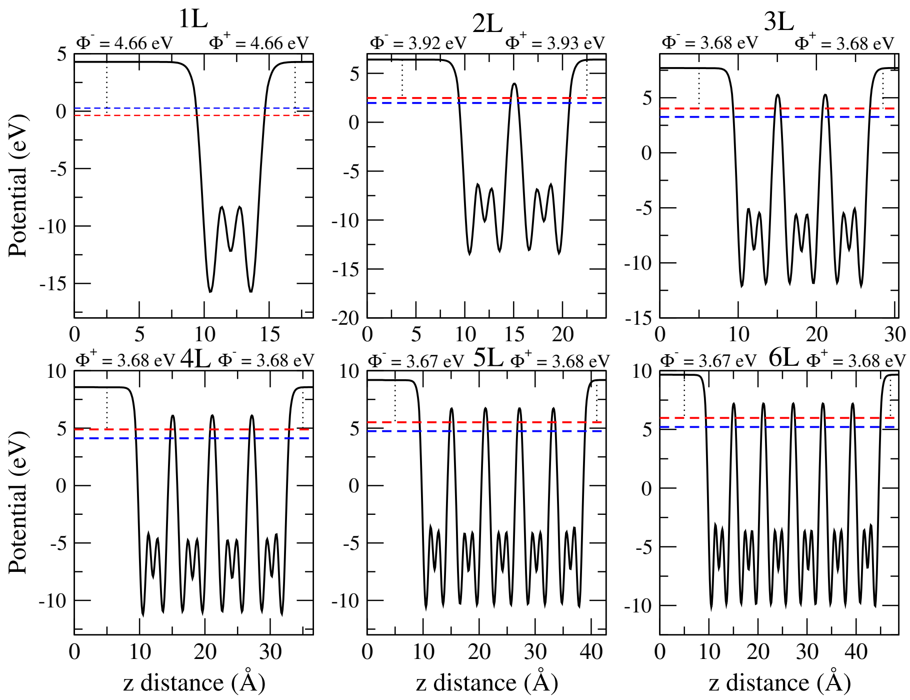

Figure S68: Electrostatic Potential energy as a function of the number of layers for $RuTe_2$. The dashed blue and red lines are respectively the Conduction Band Maximum (CBM) and the Valence Band Maximum (VBM), where, the Work Functions (Φ) are given by $\Phi^+ = E_{vac}{}^{highest} - VBM$ and $\Phi^- = E_{vac}{}^{lowest} - VBM$.

4.8 Band Offset

Table S14: Electronic levels obtained with PBE+D3 functional. The Vacuum Level for the Right (V^+) and for the Left (V^-), the Valence Band Maximum (VBM) and the Conduction Band Minimum (CBM) are shown for each Composition (MQ_2) and Number of Layers (N).

MQ_2	N	V^+	V^-	VBM	CBM
FeS_2	1L	3.476	3.476	−2.233	−1.558
FeS_2	2L	5.306	5.272	−0.222	0.192
FeS_2	3L	6.449	6.382	1.059	1.283
FeS_2	4L	7.190	7.163	1.904	2.021
FeS_2	5L	7.775	7.688	2.493	2.565
FeS_2	6L	8.212	8.084	2.947	2.957
$FeSe_2$	1L	3.192	3.192	−1.902	−1.346
$FeSe_2$	2L	4.859	4.859	−0.018	0.141

Continued on next page

Table S14 – continued from previous page

MQ_2	N	V^+	V^-	VBM	CBM
$FeTe_2$	1L	4.146	4.146	−0.444	−0.110
NiS_2	1L	3.136	3.136	−2.635	−2.088
$NiSe_2$	1L	2.840	2.840	−2.137	−2.006
OsS_2	1L	3.829	3.829	−1.527	−0.865
OsS_2	2L	5.907	5.811	0.559	1.119
OsS_2	3L	7.191	7.047	1.827	2.354
OsS_2	4L	8.085	7.869	2.721	3.179
OsS_2	5L	8.915	8.279	3.537	3.590
OsS_2	6L	7.977	7.620	2.609	2.921
$OsSe_2$	1L	3.512	3.512	−1.265	−0.533
$OsSe_2$	2L	5.334	5.304	0.581	1.220
$OsSe_2$	3L	6.463	6.388	1.703	2.319
$OsSe_2$	4L	7.196	7.156	2.486	3.075
$OsSe_2$	5L	6.242	6.190	1.562	2.112
$OsSe_2$	6L	6.777	6.637	2.088	2.562
$OsTe_2$	1L	4.307	4.307	−0.132	0.642
$OsTe_2$	2L	6.479	6.442	2.334	2.462
$OsTe_2$	3L	7.805	7.742	3.898	3.730
PdS_2	1L	2.449	2.449	−3.113	−1.918
PdS_2	2L	3.982	3.982	−1.182	−0.552
PdS_2	3L	5.044	5.044	0.060	0.430
PdS_2	4L	5.816	5.816	0.942	1.162
PdS_2	5L	6.402	6.402	1.613	1.712
PdS_2	6L	6.859	6.859	2.136	2.146
$PdSe_2$	1L	2.993	2.993	−2.463	−1.804
$PdTe_2$	1L	3.691	3.691	−0.842	−0.671

Continued on next page

Table S14 – continued from previous page

MQ_2	N	V^+	V^-	VBM	CBM
PtS_2	1L	3.509	3.509	−2.860	−1.080
PtS_2	2L	5.506	5.506	0.010	0.721
PtS_2	3L	6.815	6.815	1.435	1.944
PtS_2	4L	7.726	7.726	2.505	2.795
PtS_2	5L	8.409	8.409	3.303	3.419
PtS_2	6L	8.940	8.940	3.879	3.919
$PtSe_2$	1L	3.194	3.194	−2.483	−1.139
$PtSe_2$	2L	4.969	4.969	0.180	0.336
$PtSe_2$	3L	6.127	6.127	1.524	1.379
$PtTe_2$	1L	3.914	3.914	−0.778	−0.119
RuS_2	1L	3.750	3.750	−1.928	−1.161
RuS_2	2L	5.762	5.707	0.187	0.723
RuS_2	3L	5.923	5.806	0.389	0.806
RuS_2	4L	7.904	7.724	2.402	2.722
RuS_2	5L	8.546	8.310	3.050	3.306
RuS_2	6L	6.846	6.545	1.349	1.542
$RuSe_2$	1L	3.449	3.449	−1.629	−0.835
$RuSe_2$	2L	5.254	5.209	0.334	0.745
$RuSe_2$	3L	6.377	6.279	1.594	1.803
$RuSe_2$	4L	7.142	7.001	2.415	2.518
$RuSe_2$	5L	7.655	7.557	2.991	3.071
$RuSe_2$	6L	8.125	7.811	3.466	3.392
$RuTe_2$	1L	4.290	4.290	−0.370	0.262

Table S15: Electronic levels obtained with PBE+D3 functional plus SOC correction. The Vacuum Level for the Right (V^+) and for the Left (V^-), the Valence Band Maximum (VBM) and the Conduction Band Minimum (CBM) are shown for each Composition (MQ_2) and Number of Layers (N).

MQ_2	N	V^+	V^-	VBM	CBM
FeS_2	1L	3.476	3.476	−2.287	−1.542
FeS_2	2L	5.302	5.277	−0.232	0.195
FeS_2	3L	6.450	6.380	1.044	1.288
FeS_2	4L	7.211	7.133	1.888	2.026
FeS_2	5L	7.790	7.670	2.486	2.566
FeS_2	6L	8.231	8.071	2.934	2.967
$FeSe_2$	1L	3.190	3.190	−1.923	−1.323
$FeSe_2$	2L	4.857	4.857	−0.030	0.146
$FeTe_2$	1L	4.137	4.137	−0.454	−0.080
NiS_2	1L	3.136	3.136	−2.594	−2.017
$NiSe_2$	1L	2.839	2.839	−1.964	−1.886
OsS_2	1L	3.833	3.833	−1.637	−1.006
OsS_2	2L	5.889	5.839	0.456	0.996
OsS_2	3L	7.189	7.057	1.758	2.215
OsS_2	4L	8.075	7.882	2.665	3.043
OsS_2	5L	8.717	8.494	3.284	3.651
OsS_2	6L	7.956	7.653	2.519	2.811
$OsSe_2$	1L	3.516	3.516	−1.300	−0.688
$OsSe_2$	2L	5.367	5.313	0.555	1.008
$OsSe_2$	3L	6.462	6.410	1.697	2.140
$OsSe_2$	4L	7.210	7.167	2.444	2.892
$OsSe_2$	5L	6.237	6.316	1.468	1.892
$OsSe_2$	6L	6.777	6.656	2.001	2.388

Continued on next page

Table S15 – continued from previous page

MQ_2	N	V^+	V^-	VBM	CBM
$OsTe_2$	1L	4.309	4.309	−0.113	0.649
$OsTe_2$	2L	6.472	6.440	2.261	2.478
$OsTe_2$	3L	7.758	7.754	3.729	3.749
PdS_2	1L	2.450	2.450	−3.103	−1.934
PdS_2	2L	3.982	3.982	−1.175	−0.537
PdS_2	3L	5.045	5.045	0.066	0.441
PdS_2	4L	5.817	5.817	0.947	1.202
PdS_2	5L	6.407	6.407	1.615	1.754
PdS_2	6L	6.862	6.862	2.139	2.151
$PdSe_2$	1L	2.994	2.994	−2.286	−1.518
$PdTe_2$	1L	3.696	3.696	−0.506	−0.617
PtS_2	1L	3.514	3.514	−2.793	−0.932
PtS_2	2L	5.514	5.514	−0.030	0.872
PtS_2	3L	6.824	6.824	1.411	2.038
PtS_2	4L	7.735	7.735	2.515	2.875
PtS_2	5L	8.421	8.421	3.312	3.516
PtS_2	6L	8.951	8.951	3.892	4.024
$PtSe_2$	1L	3.200	3.200	−2.280	−1.021
$PtSe_2$	2L	4.977	4.977	−0.014	0.483
$PtSe_2$	3L	6.135	6.135	1.545	1.514
$PtTe_2$	1L	3.916	3.916	−0.377	0.162
RuS_2	1L	3.750	3.750	−2.025	−1.199
RuS_2	2L	5.763	5.708	0.190	0.699
RuS_2	3L	5.927	5.809	0.353	0.791
RuS_2	4L	7.904	7.727	2.399	2.706
RuS_2	5L	8.549	8.308	3.049	3.287

Continued on next page

Table S15 – continued from previous page

MQ_2	N	V^+	V^-	VBM	CBM
RuS_2	6L	6.851	6.533	1.348	1.512
$RuSe_2$	1L	3.448	3.448	−1.660	−0.842
$RuSe_2$	2L	5.252	5.208	0.322	0.760
$RuSe_2$	3L	6.372	6.278	1.561	1.853
$RuSe_2$	4L	7.133	7.005	2.324	2.525
$RuSe_2$	5L	7.643	7.555	2.864	3.078
$RuSe_2$	6L	8.066	7.866	3.317	3.528
$RuTe_2$	1L	4.282	4.282	−0.327	0.292

Table S16: Electronic levels obtained with HSE06 functional. The Vacuum Level for the Right (V^+) and for the Left (V^-), the Valence Band Maximum (VBM) and the Conduction Band Minimum (CBM) are shown for each Composition (MQ_2) and Number of Layers (N).

MQ_2	N	V^+	V^-	VBM	CBM
FeS_2	1L	3.483	3.483	−3.055	−1.820
FeS_2	2L	5.326	5.279	−0.757	−0.032
FeS_2	3L	6.479	6.383	0.569	1.066
FeS_2	4L	7.262	7.125	1.448	1.805
FeS_2	5L	7.841	7.656	2.061	2.336
FeS_2	6L	8.284	8.058	2.515	2.515
$FeSe_2$	1L	3.195	3.195	−2.589	−1.627
$FeSe_2$	2L	4.875	4.875	−0.488	−0.114
$FeTe_2$	1L	4.150	4.150	−0.853	−0.300
NiS_2	1L	3.147	3.147	−3.093	−1.873
$NiSe_2$	1L	2.841	2.841	−2.532	−1.998
OsS_2	1L	3.832	3.832	−2.426	−0.794

Continued on next page

Table S16 – continued from previous page

MQ_2	N	V^+	V^-	VBM	CBM
OsS_2	2L	5.896	5.827	−0.255	1.182
OsS_2	3L	7.194	7.046	1.074	2.397
OsS_2	4L	8.081	7.872	1.987	3.224
OsS_2	5L	8.745	8.456	2.652	3.807
OsS_2	6L	7.990	7.614	1.894	2.962
$OsSe_2$	1L	3.508	3.508	−2.119	−0.481
$OsSe_2$	2L	5.329	5.296	−0.113	1.254
$OsSe_2$	3L	6.449	6.392	1.168	2.331
$OsSe_2$	4L	7.212	7.127	2.055	3.067
$OsSe_2$	5L	6.272	6.162	1.140	2.100
$OsSe_2$	6L	6.773	6.627	1.665	2.567
$OsTe_2$	1L	4.302	4.302	−0.837	0.740
$OsTe_2$	2L	6.474	6.425	2.053	2.387
$OsTe_2$	3L	7.749	7.742	3.744	3.617
PdS_2	1L	2.435	2.435	−3.851	−1.644
PdS_2	2L	3.958	3.958	−1.905	−0.302
PdS_2	3L	5.014	5.014	−0.650	0.663
PdS_2	4L	5.783	5.783	0.239	1.386
PdS_2	5L	6.366	6.366	0.916	1.928
PdS_2	6L	6.824	6.824	1.442	2.352
$PdSe_2$	1L	2.988	2.988	−2.887	−1.803
$PdTe_2$	1L	3.683	3.683	−1.125	−0.682
PtS_2	1L	3.510	3.510	−3.542	−0.925
PtS_2	2L	5.507	5.507	−0.474	0.855
PtS_2	3L	6.815	6.815	1.046	2.069
PtS_2	4L	7.726	7.726	2.166	2.924

Continued on next page

Table S16 – continued from previous page

MQ_2	N	V^+	V^-	VBM	CBM
PtS_2	5L	8.409	8.409	2.979	3.541
PtS_2	6L	8.938	8.938	3.562	4.040
$PtSe_2$	1L	3.191	3.191	−2.957	−1.057
$PtSe_2$	2L	4.959	4.959	−0.166	0.375
$PtSe_2$	3L	6.113	6.113	1.280	1.398
$PtTe_2$	1L	3.905	3.905	−1.118	−0.060
RuS_2	1L	3.754	3.754	−2.791	−1.134
RuS_2	2L	5.777	5.708	−0.296	0.733
RuS_2	3L	5.942	5.800	−0.075	0.809
RuS_2	4L	7.930	7.714	1.943	2.720
RuS_2	5L	8.570	8.261	2.618	3.339
RuS_2	6L	6.879	6.492	0.928	1.570
$RuSe_2$	1L	3.447	3.447	−2.506	−0.822
$RuSe_2$	2L	5.260	5.199	−0.072	0.725
$RuSe_2$	3L	6.391	6.261	1.252	1.775
$RuSe_2$	4L	7.164	6.973	2.077	2.478
$RuSe_2$	5L	7.733	7.473	2.664	2.986
$RuSe_2$	6L	8.172	7.761	3.129	3.340
$RuTe_2$	1L	4.284	4.284	−0.967	0.298

Table S17: Electronic energies obtained with HSE06 functional plus SOC correction. The Ionization Energy for the Right (EI^{+}) and for the Left (EI^{-}), The Electron Affinity for the Right (EA^{+}) and for the Left (EA^{-}) are shown for each Composition (MQ_2) and Number of Layers (N).

MQ_2	N	EI^{+}	EI^{-}	EA^{+}	EA^{-}
FeS_2	1L	6.592	6.592	5.287	5.287
FeS_2	2L	6.089	6.050	5.351	5.312
FeS_2	3L	5.925	5.827	5.408	5.310
FeS_2	4L	5.851	5.664	5.474	5.287
FeS_2	5L	5.801	5.584	5.518	5.300
FeS_2	6L	5.801	5.543	5.778	5.520
$FeSe_2$	1L	5.803	5.803	4.796	4.796
$FeSe_2$	2L	5.372	5.372	4.981	4.981
$FeTe_2$	1L	5.003	5.003	4.410	4.410
NiS_2	1L	6.199	6.199	4.949	4.949
$NiSe_2$	1L	5.200	5.200	4.718	4.718
OsS_2	1L	6.371	6.371	4.772	4.772
OsS_2	2L	6.237	6.213	4.820	4.796
OsS_2	3L	6.186	6.051	4.934	4.798
OsS_2	4L	6.140	5.954	4.983	4.797
OsS_2	5L	6.148	6.273	4.679	4.804
OsS_2	6L	6.165	5.843	5.116	4.794
$OsSe_2$	1L	5.668	5.668	4.149	4.149
$OsSe_2$	2L	5.501	5.445	4.320	4.264
$OsSe_2$	3L	5.285	5.252	4.296	4.263
$OsSe_2$	4L	5.213	5.124	4.343	4.254
$OsSe_2$	5L	5.222	5.242	4.388	4.407
$OsSe_2$	6L	5.195	5.068	4.379	4.252

Continued on next page

Table S17 – continued from previous page

MQ_2	N	V^+	V^-	VBM	CBM
$OsTe_2$	1L	5.121	5.121	3.557	3.557
$OsTe_2$	2L	4.487	4.443	4.064	4.020
$OsTe_2$	3L	4.126	4.178	4.066	4.118
PdS_2	1L	6.277	6.277	4.095	4.095
PdS_2	2L	5.857	5.857	4.246	4.246
PdS_2	3L	5.660	5.660	4.341	4.341
PdS_2	4L	5.540	5.540	4.358	4.358
PdS_2	5L	5.453	5.453	4.401	4.401
PdS_2	6L	5.382	5.382	4.470	4.470
$PdSe_2$	1L	5.698	5.698	4.505	4.505
$PdTe_2$	1L	4.477	4.477	4.316	4.316
PtS_2	1L	6.991	6.991	4.293	4.293
PtS_2	2L	6.028	6.028	4.509	4.509
PtS_2	3L	5.801	5.801	4.661	4.661
PtS_2	4L	5.559	5.559	4.731	4.731
PtS_2	5L	5.432	5.432	4.782	4.782
PtS_2	6L	5.374	5.374	4.804	4.804
$PtSe_2$	1L	5.950	5.950	4.136	4.136
$PtSe_2$	2L	5.327	5.327	4.445	4.445
$PtSe_2$	3L	4.821	4.821	4.589	4.589
$PtTe_2$	1L	4.625	4.625	3.687	3.687
RuS_2	1L	6.643	6.643	4.926	4.926
RuS_2	2L	6.070	6.002	5.069	5.001
RuS_2	3L	6.059	5.915	5.154	5.010
RuS_2	4L	5.990	5.776	5.226	5.012
RuS_2	5L	5.956	5.642	5.253	4.939

Continued on next page

Table S17 – continued from previous page

MQ_2	N	V^+	V^-	VBM	CBM
RuS_2	6L	5.957	5.552	5.345	4.940
$RuSe_2$	1L	5.983	5.983	4.275	4.275
$RuSe_2$	2L	5.342	5.282	4.517	4.457
$RuSe_2$	3L	5.168	5.042	4.560	4.434
$RuSe_2$	4L	5.169	4.990	4.670	4.491
$RuSe_2$	5L	5.185	4.935	4.729	4.479
$RuSe_2$	6L	5.133	4.836	4.637	4.340
$RuTe_2$	1L	5.200	5.200	3.948	3.948

References

1 Besse, R.; Lima, M. P.; Da Silva, J. L. F. First-Principles Exploration of Two-Dimensional Transition Metal Dichalcogenides Based on Fe, Co, Ni, and Cu Groups and Their van der Waals Heterostructures. *ACS Appl. Energy Mater.* **2019**, *2*, 8491–8501, DOI: 10.1021/acsaem.9b01433.

2 Haastrup, S.; Strange, M.; Pandey, M.; Deilmann, T.; Schmidt, P. S.; Hinsche, N. F.; Gjerding, M. N.; Torelli, D.; Larsen, P. M.; Riis-Jensen, A. C.; Gath, J.; Jacobsen, K. W.; Mortensen, J. J.; Olsen, T.; Thygesen, K. S. The Computational 2D Materials Database: high-throughput modeling and discovery of atomically thin crystals. *2D Mater.* **2018**, *5*, 042002, DOI: 10.1088/2053-1583/aacfc1.

3 Gjerding, M. N.; Taghizadeh, A.; Rasmussen, A.; Ali, S.; Bertoldo, F.; Deilmann, T.; Knøsgaard, N. R.; Kruse, M.; Larsen, A. H.; Manti, S.; Pedersen, T. G.; Petralanda, U.; Skovhus, T.; Svendsen, M. K.; Mortensen, J. J.; Olsen, T.; Thygesen, K. S. Recent progress of the Computational 2D Materials Database (C2DB). *2D Mater.* **2021**, *8*, 044002, DOI: 10.1088/2053-1583/ac1059.
